# Synthesis of Silsesquioxanes with Substituted Triazole Ring Functionalities and Their Coordination Ability †

**DOI:** 10.3390/molecules26020439

**Published:** 2021-01-15

**Authors:** Monika Rzonsowska, Katarzyna Kozakiewicz, Katarzyna Mituła, Julia Duszczak, Maciej Kubicki, Beata Dudziec

**Affiliations:** 1 Department of Organometallic Chemistry, Faculty of Chemistry, Adam Mickiewicz University in Poznań, Uniwersytetu Poznańskiego 8, 61-614 Poznań, Poland; k.kozakiewicz96@gmail.com (K.K.); katarzyna.mitula@gmail.com (K.M.); julia.duszczak@amu.edu.pl (J.D.); 2 Centre for Advanced Technologies, Adam Mickiewicz University in Poznań, Uniwersytetu Poznańskiego 10, 61-614 Poznań, Poland; 3 Faculty of Chemistry, Adam Mickiewicz University in Poznań, Uniwersytetu Poznańskiego 8, 61-614 Poznań, Poland; mkubicki@amu.edu.pl

**Keywords:** polyhedral oligomeric silsesquioxane (SQs), click chemistry, CuAAC, coordination compounds, bidentate ligand

## Abstract

A synthesis of a series of mono-T_8_ and difunctionalized double-decker silsesquioxanes bearing substituted triazole ring(s) has been reported within this work. The catalytic protocol for their formation is based on the copper(I)-catalyzed azide-alkyne cycloaddition (CuAAC) process. Diverse alkynes were in the scope of our interest—i.e., aryl, hetaryl, alkyl, silyl, or germyl—and the latter was shown to be the first example of terminal germane alkyne which is reactive in the applied process’ conditions. From the pallet of 15 compounds, three of them with pyridine-triazole and thiophenyl-triazole moiety attached to T_8_ or DDSQ core were verified in terms of their coordinating properties towards selected transition metals, i.e., Pd(II), Pt(II), and Rh(I). The studies resulted in the formation of four SQs based coordination compounds that were obtained in high yields up to 93% and their thorough spectroscopic characterization is presented. To our knowledge, this is the first example of the DDSQ-based molecular complex possessing bidentate pyridine-triazole ligand binding two Pd(II) ions.

## 1. Introduction

Polyhedral oligomeric silsesquioxanes (SQs) are a large family of compounds that feature diverse structures with Si-O-Si linkages and tetrahedral Si vertices—i.e., random, amorphous, ladder, and cage-like—and the architecture of the latter has attracted considerable scientific interest. It is due to the presence of the inorganic, rigid core (thermal stability, chemical resistance) and organic moieties attached to it (tunable processability) which is the essence of hybrid materials. Functionalized SQs derivatives may be regarded as their nanosized, smallest fragments and precursors that affect and drive the directions of their potential applications [1,2,3,4,5,6]. Significant development of catalytic protocols for effective and selective anchoring of respective organic functionality to the SQs core has been observed during the last years. The crucial aspect of this is the presence of a proper prefunctional moiety at the Si-O-Si framework, enabling its modification, e.g., Si-H, Si-OH, Si–CH=CH_2_ units, etc. This, in turn, influences the selection of a respective catalytic procedure for this purpose, e.g., hydrosilylation, cross-metathesis, *O*-silylation, Friedel-Crafts, silylative, Heck, Suzuki, or Sonogashira coupling reactions [2,7,8,9,10,11,12,13,14,15,16,17,18,19,20]. Among these methods, the copper(I)-catalyzed azide-alkyne cycloaddition (CuAAC) may be an alternative but the only route to yield substituted 1,4-triazole ring functionalities regioselectively [21,22,23]. This is a powerful synthetic tool to build covalent connections between dissimilar units, and since its discovery in 2002 (independently by Sharpless, Fokin, and Meldal groups), it is widely utilized in (bio-)organic, medicinal and for some time now, also in surface/materials chemistry [24,25,26,27]. Despite the simplicity of the reaction, the immense development in the catalyst design, incl. stabilizing ligands, has be observed in the past few years. This may be visible in the aspect of functional group diversity in both reagents and their resilience to the CuAAC reaction conditions, i.e., solvent type, temperature, time, etc. The variety of Cu-based catalysts for this process requires the presence of the Cu(I) species at the highest concentration whether it is introduced in this state or generated in situ [26]. On the other hand, the Cu(I) favors the Glasser coupling of terminal alkynes [28], so the CuAAC conditions should be optimized to avoid the formation of by-products. For this, the in situ creation of Cu(I) ions seems reasonable [24]. As a result of this evolvement, the process gained popularity in the chemistry of silsesquioxanes as well. There are some examples of CuAAC methodology applied for silsesquioxanes to introduce the triazole moiety(-ies) substituted at 1,4 positions with the SQs core and organic group. The process may be applied in the case of mono- and octa-substituted T_8_ SQs, linked with the Si-O-Si core via mainly alkyl [29,30,31,32,33,34,35,36] but there are also reports on a phenyl group [37,38]. There are also few examples of using di- substituted double-decker (DDSQ) [39,40] or ladder [41] silsesquioxanes [42]. 

The application of the resulting products with 1,4-triazole ring(s) depends on the kind of alkyne moiety used as a reagent. Due to the interesting photoelectronic properties of SQs-based systems with aryl-triazole groups, they could be possibly used as luminescent materials with enhanced thermal resistance [29,41,43,44,45]. One of the promising branches of their application is materials chemistry, e.g., as polymers or dendrimers modifiers/synthons [30,39,42,46,47,48,49,50,51,52,53,54]. They may be also found in macromolecular SQs-based surfactants of amphiphilic and self-assembly or encapsulation properties [31,55,56]. Additionally, the CuAAC methodology serves as a tool for the formation of SQs functionalized peptide dendrimers [35] or glycoconjugates and in targeted bioimaging [57,58].

Catalysis has become a very prospective direction of employing the SQs with 1,4-substituted triazole rings, as catalysts by themselves, e.g., in asymmetric Michael or Aldol reactions [32,33]. Even though there have been still very few reports in this area of interest. One of them reported on the potential coordinating character of a pyridine-triazole moiety attached to the SQs core and revealed its use as a ligand for Pd-complexation. Interestingly, this compound was found to be active in Suzuki–Miyaura cross-coupling [59].

**Figure 1:**
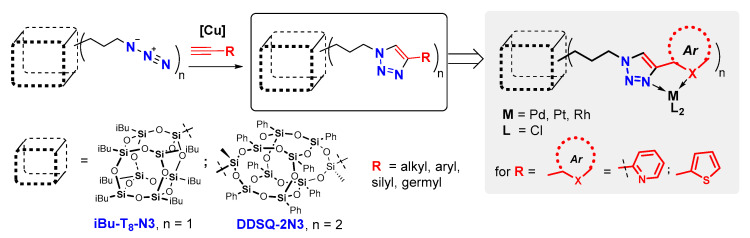
Mono-T_8_ and difunctionalized DDSQ silsesquioxanes with Substituted Triazole Ring and the coordinating ability of the pyridine- and thiophenyl-derivatives towards selected TM ions, presented in this work.

Herein, we present our studies on the copper(I)-catalyzed azide-alkyne cycloaddition process of two different types of silsesquioxanes with the variation of alkynes bearing aryl, hetaryl, alkyl, silyl, siloxyl, or even germyl groups. As a result, we report on the preparation of mono-T_8_ and difunctionalized double-decker silsesquioxanes with substituted triazole ring(s). The second part of the paper is focused on the application of selected SQs with hetaryl substituted triazole moieties as potential ligands in complexing reaction with transition metals (Pd, Rh, Pt), resulting in the formation of respective SQs-based coordination systems (Figure 1).

## 2. Results and Discussion

### 2.1. The Copper(I)-Catalyzed Azide-Alkyne Cycloaddition (CuAAC) Using ***iBuT_8_-N3*** and ***DDSQ-2N3***

**Figure 2:**
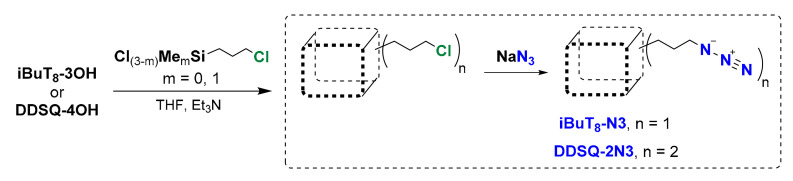
General route for the synthesis of azide-derivatives **iBuT_8_-N3** and **DDSQ-2N3**.

In the first step, the starting precursors, i.e., the azidopropyl-derivative(s) of mono-**iBuT_8_-N3** and di-**DDSQ-2N3** were prepared in a sequence of hydrolytic condensation of respective silanol precursor of SQs and chlorosilane followed by nucleophilic substitution with NaN_3_ [60,61] (Figure 2). The idea of the synthetic path is presented below.

**Figure 3:**
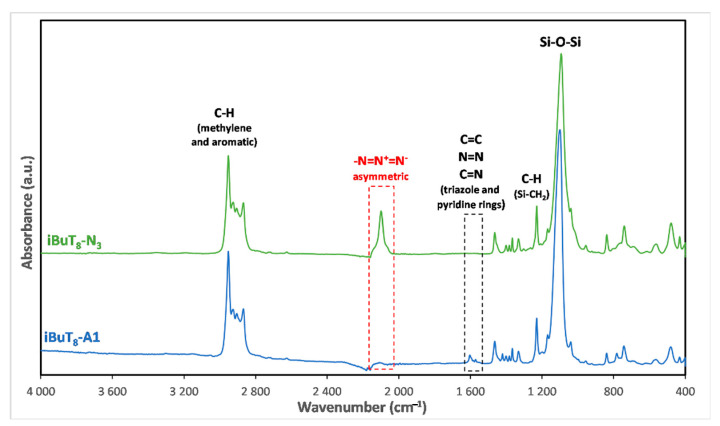
FT-IR spectra of **iBuT_8_-N3** and **iBuT_8_-A1** after completion of CuAAC coupling reaction.

The two SQs-based azides **iBuT_8_-N3** and **DDSQ-2N3** were used as reagents in CuAAC coupling process with a variety of alkynes bearing aryl, alkyl, silyl, and germyl functionalities. The reaction progress was monitored by FT-IR, due to the large mass of the product eliminating the possibility of using GC or GC-MS and confirmed by ^1^H-NMR. The representative FT-IRs are presented in Figure 3. For all alkynes tested nearly complete conversion of SQs azides was observed within up to 3 days which depended on the type of reaction conditions and Cu catalyst. 

The stacked FT-IR spectra of starting material **iBuT_8_-N3** and the selected product with 4-pyridine-triazole group **iBuT_8_-A1** are depicted in Figure 3. The established reaction conditions resulted in the complete conversion of azide (-N=N^+^=N-) group in **iBuT_8_-N3**, confirmed by the disappearance of respective bands attributed to stretching asymmetric vibrations of -N=N- at ca. ῡ = 2098 cm^−1^ (marked in Figure 3). For the CuAAC reaction product, i.e., **iBuT_8_-A1**, there are new bands in the spectrum, characteristics of C=C, C=N, N=N stretching vibrations from triazole as well as pyridine ring at ca. ῡ = 1603 cm^−1^ and ῡ = 1571 cm^−1^ that confirm the formation of the desired product.

We based on two types of Cu sources, i.e., CuSO_4_ with sodium ascorbate [40,59] and CuBr with PMDTA [33]. At first, special conditions were created for the reduction of Cu(II) in situ to Cu(I) and then to maintain the introduction of Cu(I) in this oxidation state into the reaction. The main target was to perform the reaction until full conversion of SQ-based azides to avoid the problematic isolation issues of resulting SQ-products with substituted triazole rings(s) from unreacted SQ-based azides. The results of the reactions conducted to obtain products with substituted triazole ring(s) are collected in Table 1 for T_8_-derivatives and in Table 2 for DDSQ-derivatives. 

The results of DDSQ-based systems with substituted triazole rings are collected in Table 2 and involves the selective formation of DDSQ-compounds with the two abovementioned triazole moieties. The spectrum of used alkynes varies as they contain aryl, hetaryl, alkyl, and silyl derivatives of commercial availability. Additionally, we tested ethynyl(triethyl)germane (A9) and ethynylsiloxysubstituted-iBuT_8_ (A8) to verify their potential in the CuAAC process.

The tested reaction conditions based on Cu(II) and Cu(I) catalysts seem to be analogous in the case of less demanding alkynes, i.e., simple aryl or alkyl derivatives. Interestingly, for the 5-hexynenitrile, the applied catalytic conditions did not affect the present -CN moiety that in general may also be reactive and susceptible to alkyne-azide coupling reaction conditions to form respective 5-substituted tetrazoles [62]. For this, the presence of a reactive -CN moiety could be used in further modifications of the obtained products: **iBuT_8_-A4** and **DDSQ-2A4**. The reactivity of ethynylsilane (A9), ethynylgermane (A10) and also ethynylsiloxysubstituted iBuT_8_ (A8) compounds was tested with positive results. However, the use of silyl (A9) or germyl (A10) alkyne proceeded with >99% conversion of SQs-based azides (**iBuT_8_-N3** and **DDSQ-2N3**) only when modified reaction conditions with Cu(I) [33] were applied (heating at 45 °C). Even though, for ethynylsiloxysubstituted-iBuT_8_ (A8) up to 10% of unreacted **iBuT_8_-N3** was observed. It could be separated from the resulting product **iBuT_8_-A8** during the purification with the use of chromatography column and proper eluent selection (hexane:DCM 3:1 for separation of **iBuT_8_-N3** from **iBuT_8_-A8**). Lower reactivity of A8 may derive from the presence of oxygen as the silicon atom in the vicinity of ethynyl-moiety and its electron-withdrawing impact. It should be noted that ethynylsilanes exhibit reactivity in this process, but conditions created by us seem to be milder for lower reaction temperature [63]. On the other hand, it would be the first example for ethynylgermane (A10) to exhibit high reactivity in the CuAAC reaction. One report on the formation of 4-germyl-substituted triazole ring derivative concerns using internal alkyne, i.e., 3-(trimethylgermyl)-2-propynal [64]. Additionally, the reports on the reactivity of the ethynylsiloxy-moiety (meaning A8) in the CuAAC process are very scarce [65]. 

**Figure 4:**
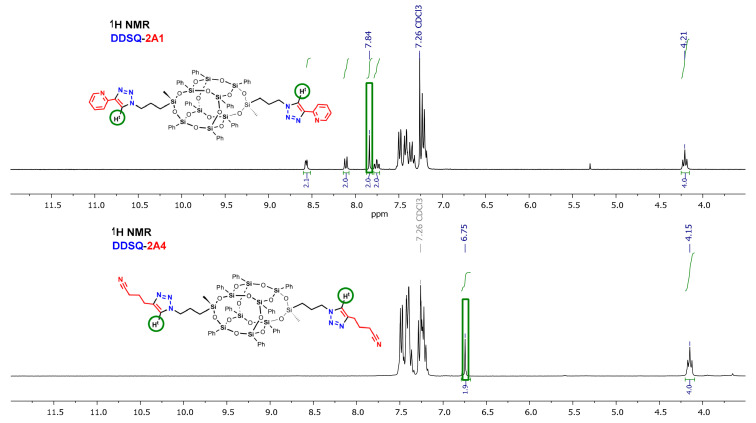
Selected range of stacked ^1^H-NMR spectra of **DDSQ-2A1** and **DDSQ-2A4**.

**Figure 5:**
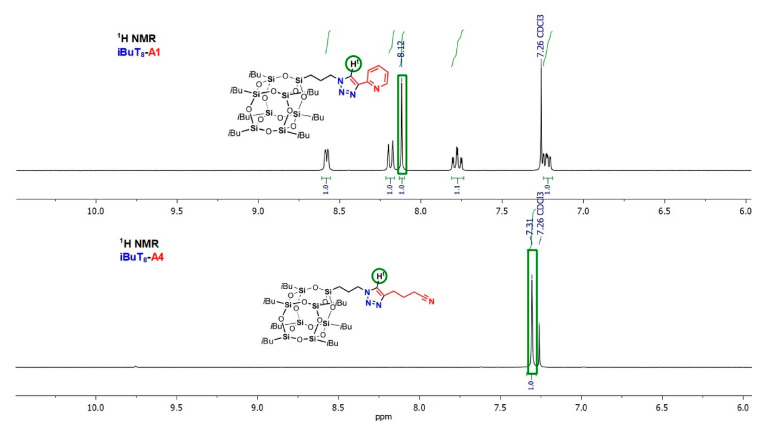
Selected range of stacked ^1^H-NMR spectra of **iBuT_8_-A1** and **iBuT_8_-A4**.

An interesting relationship was found for ^1^H-NMR analyses of DDSQs bearing triazole ring substituted at 4-positition with an aryl (**DDSQ-2A1**) and alkyl (**DDSQ-2A4**) group. The resonance line of a very significant triazole proton N=C-H^t^ at 5*H*-position of triazole ring depends on the type of the moiety at 4-position of the latter. The crucial aspect may be its electronic property and the respective shielding effect of alkyl and deshielding effect characteristic for the aryl moiety presence. It affects the N=C-H^t^ signal shift and it is upfield for **DDSQ-2A1** to be present at 6.75 ppm and downfield for **DDSQ-2A4**, to appear at 7.84 ppm, which gives a total change in resonance lines of 1.09 ppm (Figure 4). Due to the presence of a triazole, aromatic ring, this effect is also insensibly perceptible for -CH_2_- group at 1*N*-position of this ring (for **DDSQ-2A1** δ = 4.15 ppm and **DDSQ-2A4** δ = 4.21 ppm) (Figure 4). This is a notable difference in chemical shifts of N=C-H^t^ at triazole ring for its alkyl and aryl derivatives when compared with analogous compounds of iBu-SQs, i.e., **iBuT_8_-A4** (alkyl δ = 7.31 ppm) and **iBuT_8_-A1** (aryl δ = 8.12 ppm) that equals 0.82 ppm (Figure 5). It is even more significant when comparing analogous products with alkyl groups at triazole ring but with diverse Si-O-Si cores, i.e., **DDSQ-2A4**, N=C-H^t^ proton present at 6.75 ppm with **iBuT_8_-A4**, =C-H^t^ at 7.31 ppm. These differences in result may be explained by the presence and electronic effect of the DDSQ core with phenyl substituents. 

### 2.2. X-ray Analysis of ***DDSQ-2A1***

**Figure 6:**
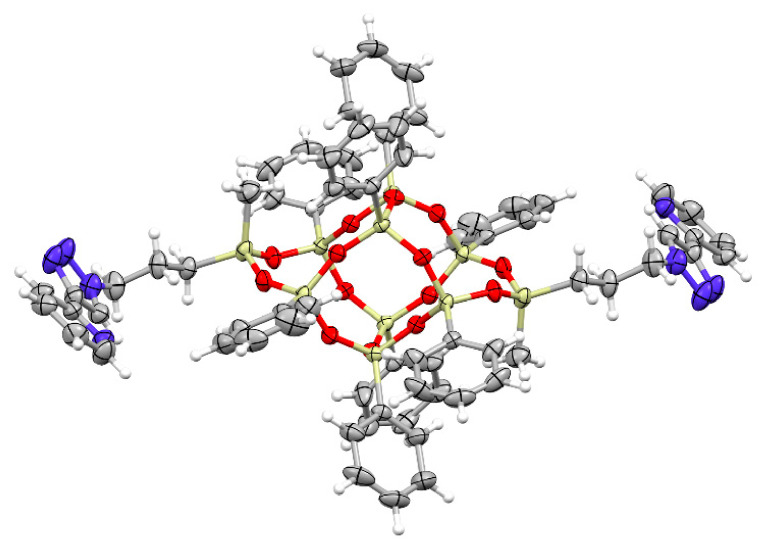
A perspective view of the molecule. Ellipsoids are drawn at the 50% probability level, hydrogen atoms are shown as spheres of arbitrary radii (grey-C, white-H, blue-N, red-O, yellow-Si).

A DDSQ-based pyridine-triazole derivative, i.e., **DDSQ-2A1** proved to be a solid and acquired the form of crystals amenable to X-ray crystal structure determination (Figure 6). The molecule is *C_i_*-symmetrical, as it lies across the center of inversion in the space group *P2_1_/c*. The structure of the core may be described as built of four rings, two 8-membered (four Si, four O), and two 10-membered (five Si, five O), which can be noted as 8^2^10^2^. The geometry of the core of the molecule is determined by two factors: one rigid—Si-O distance, which has a very narrow spread (mean value 1.615(8) Å), and one flexible Si-O-Si angles (140.77(16)°–162.43(16)°). Similar tendencies were noted in similar molecules [16,66]. The architecture of the crystal is determined by weak but numerous interactions (C-H···O, C-H···π, π···π etc.). These multiple interactions give rise to quite significant interaction energies. Calculations with PIXEL method give results as high as −160.5, −95.7, and −85.4 kJ/mol for the three highest interaction energies between molecules, and −555.5 kJ/mol as total packing energy [67,68].

All of the T_8_ and DDSQ-based compounds with substituted triazole ring(s) were isolated in high, up to 90% yields. They are air-stable white or light-yellow solids with good solubility in DCM, CHCl_3_, THF, toluene. The solubility in MeOH, MeCN and for hexane depends on the type of SQ’s core, i.e., **iBuT_8_** derivatives are more soluble than **DDSQ**s.

### 2.3. SQs-Based Pyridyl- and Thiophenyl-Triazole Derivatives (***iBuT_8_-A1**, **DDSQ-2A1**, **iBuT_8_-A7***) as Bidentate Ligands in the Formation of Coordination Complexes with Selected Transition Metals (TM = Pd, Pt, Rh)

**Figure 7:**
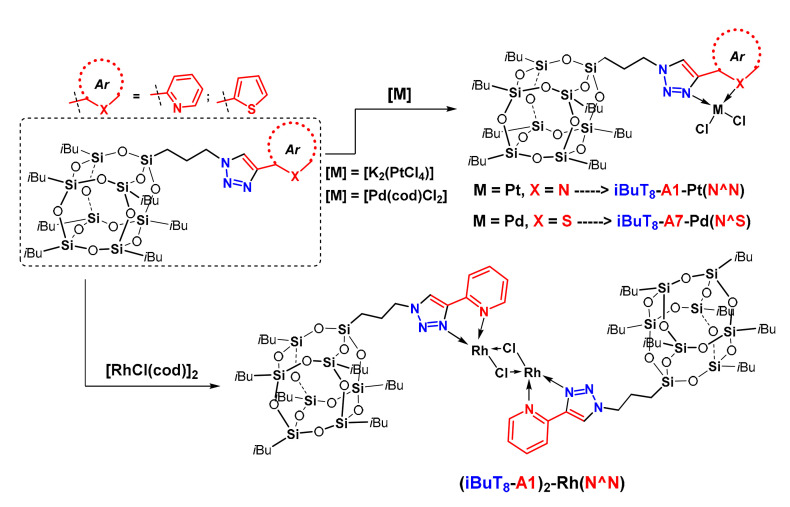
General procedure for the synthesis of T_8_-based N^N and N^S type mononuclear coordination compounds with Pd(II) (**iBuT_8_-A7-Pt(N^S)**), Pt(II) (**iBuT_8_-A1-Pt(N^N)**), and binuclear with Rh(I) ((**iBuT_8_-A1)_2_-Rh(N^N)**).

**Figure 8:**
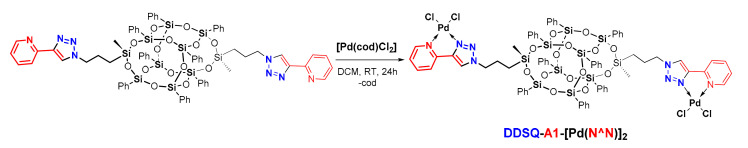
General procedure for the synthesis of DDSQ-based N^N coordination compound with Pd(II) (**DDSQ-A1-[Pd(N^N)]_2_**).

The next step was to verify the coordination properties of selected T_8_ and DDSQ products type possessing heteroatom at 4*C* triazole ring, i.e., N (**iBuT_8_-A1**, **DDSQ-2A1**) and S (**iBuT_8_-A7**). Using 2-ethynylpyridine and 2-ethynylthiophene derivatives created the possibility to form bidentate ligands of N^N and N^S kind donation. For this purpose, we chose TM metals that are known to form coordination compounds with SQs-based ligands, i.e., Pd(II) [59,69], Rh(I) [70], and Pt(II) [71].The general scheme for using T_8_-type ligands, i.e., **iBuT_8_-A1** and **iBuT_8_-A7** is disclosed in Figure 7 for Pd(II), Pt(II), and Rh(I) and DDSQ-based ligand with Pd(II) in Figure 8. 

The analogous verification was performed in terms of **DDSQ-2A1** possessing bidentate N^N ligand Pd(II). The 1:2 (ligand: metal) stoichiometry of the reaction enabled the formation of a molecular system with two Pd(II) ions captured to the opposite parts of the DDSQ core (Figure 8). 

To our knowledge, this is the first example of the DDSQ-based molecular complex possessing a bidentate pyridine-triazole ligand with coordination TM Pd(II) ion. Furthermore, it is an interesting example of using difunctionalized DDSQ compounds to anchor metal ions and the reports on these systems have been still profoundly limited [71,72].

**Figure 9:**
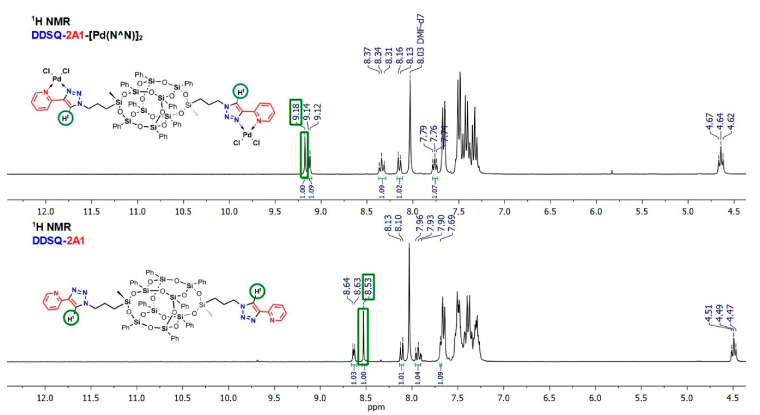
Selected range of stacked ^1^H-NMR spectra of **DDSQ-A1** and **DDSQ-A1-[Pd(N^N)]_2_**.

For the reaction aiming at palladium and rhodium complexes, their cyclooctadiene precursors were used and for platinum, the tetrachloroplatinate(II) was applied. The mononuclear compounds **iBuT_8_-A7-Pt(N^S)** and **iBuT_8_-A1-Pt(N^N)** are air-stable, pale yellow solids. The dinuclear Rh(I) based complex ((**iBuT_8_-A1)_2_-Rh(N^N)**) is rather an air- and moisture sensitive orange solid and its synthesis was performed with the use of the Schlenk technique. The iBuT_8_-derivatives are soluble in DCM, CHCl_3_, THF, toluene, and of very low solubility in methanol. The DDSQ-based Pd(II) complex **DDSQ-A1-[Pd(N^N)]_2_** is an air-stable pale yellow solid with very limited solubility in DCM, CHCl_3_, and THF and soluble in DMF and DMSO. The four coordination SQ-based compounds were isolated in yields 55%–93% and characterized using spectroscopic analysis proving their formation (for details see ESI). The respective comparison of the ^1^H-NMR stacked spectra of ligand **DDSQ-A1** and respective complex **DDSQ-A1-[Pd(N^N)]_2_** are presented below (Figure 9). 

The presence of Pd with its chloro-ligands in **DDSQ-A1-[Pd(N^N)]_2_** affects the polarity of the complex and restricts its solubility in a common, less polar solvent and for this reason DMF-*d*_7_ was selected in order to compare ^1^H-NMR spectra. As expected from the results obtained for the iBuT_8_-based Pd, Pt, and Rh complexes and from the literature reports [59], the placement of resonance line of the triazole proton N=C-H^t^ is susceptible to the chemical surrounding and presence of a different type of TM ion. However, in general, in each complex its shift is downfield significantly. For **DDSQ-A1-[Pd(N^N)]_2_** N=C-H^t^ there is a notable difference in its chemical shift to appear at δ = 9.18 ppm when compared with a bare ligand, i.e., **DDSQ-A1**: N=C-H^t^ at δ = 8.53 ppm (Figure 9). Additionally, the resonance lines derived from the pyridine ring are also shifted downfield due to the changes in the electron density on the hetaryl moiety while coordinating to Pd ion, especially for the =C-H^5^.

## 3. Materials and Methods 

### 3.1. Materials

The chemicals were purchased from the following sources: Hybrid Plastics (Hybrid Plastics, Hattiesburg, MS, USA) for DDSQ tetrasilanol form (C_48_H_44_O_14_Si_8_) (DDSQ-4OH), trisilanol (C_28_H_66_O_12_Si_7_)(iBuT_8_-3OH); Sigma-Aldrich (Saint Louis, MO, USA) for: dichloromethane (DCM), tetrahydrofuran (THF), dimethylformamide (DMF), toluene, methanol, acetonitrile (MeCN), chloroform (CHCl_3_), hexane, chloroform-*d*, dimethyl sulfoxide-*d*_6_ (DMSO-*d*_6_), dichloromethane-*d*_2_ (DCM-*d*_2_), (dimethylphenylsilyl)acetylene, 2-ethynylpyridine, *N*-methyl-*N*-propargylbenzylamine, 3-butynylbenzene, phenylacetylene, n-heptyne, 1,4-diethynylbenzene, 5-hexynenitrile, 2-ethynylthiophene; ABCR (ABCR, Karlsruhe, Germany) for dichloro(3-chloropropyl)methylsilane, molecular sieves, triethylamine, and silica gel 60. Chemat (Gdansk, Poland) for: sodium L-ascorbate crystalline, ammonium chloride, copper(II) sulfate pentahydrate, copper(I) bromide, *N*,*N*,*N*′,*N*′,*N*′′-pentamethyldiethylenetriamine (PMDTA), sodium azide, sodium sulfate anhydrous, dichloro(1,5-cyclooctadiene)palladium(II), chloro(1,5-cyclooctadiene)rhodium(I) dimer, potassium tetrachloroplatinate(II). Tetrahydrofuran (THF) was refluxed over sodium/benzophenone and distilled. Triethylamine (Et_3_N) was distilled over calcium hydride before use. DMF was stored under argon. Ethynyl(triethyl)germane (A10) and ethynyl(dimethylsiloxy)hepta(i-butyl)octasilsesquioxane (A8) was synthesized according to the literature procedures [73,74]. (3-chloropropyl)hepta(i-butyl)octasilsesquioxane (iBuT_8_-Cl) and DDSQ-2Cl were obtained via corner- and side-capping hydrolytic condensation procedure, and mono- and diazido-functionalized silsesquioxanes (**iBuT_8_-N3**, **DDSQ-2N3**) were synthesized via nucleophilic substitution according to the literature procedures [60,61]. All syntheses were conducted under argon atmosphere using standard Schlenk-line and vacuum techniques.

### 3.2. Methods

Nuclear magnetic resonance spectroscopy (NMR) measurements (^1^H, ^13^C, and ^29^Si NMR) were conducted using spectrometers: Bruker Ultrashield 300 MHz and 400 MHz respectively (Bruker, Faellanden, Switzerland) with CDCl_3_, CD_2_Cl_2_, DMF-*d*_7_, and DMSO-*d*_6_ as a solvent. Chemical shifts are reported in ppm with reference to the residual solvent signal peaks for ^1^H and ^13^C and to TMS for ^29^Si.

Fourier transform-infrared (FT-IR) spectra were recorded on a Nicolet iS5 (Thermo Scientific, Waltham, MA, USA) spectrophotometer equipped with a SPECAC Golden Gate, diamond ATR unit with a resolution of 2 cm^−1^. In all cases, 16 scans were collected to record the spectra in a range of 4000–430 cm^−1^.

Elemental analyses (EA) were performed using a Vario EL III instrument (Elementar Analysensysteme GmbH, Langenselbold, Germany).

High-resolution mass spectra (HRMS) were obtained using Impact HD mass spectrometerQ-TOF type instrument equipped with electrospray ion source (Bruker Daltonics, GmbH, Bremen, Germany). The sample solutions (DCM:MeOH) were infused into the ESI source by a syringe pump (direct inlet) at the flow rate of 3 µL/min. The instrument was operated under the following optimized settings: endplate voltage 500 V; capillary voltage 4.2 kV; nebulizer pressure 0.3 bar; dry gas (nitrogen) temperature 200 °C; dry gas flow rate 4 L/min. The spectrometer was previously calibrated with the standard tune mixture.

X-ray crystallography. Diffraction data were collected by the ω-scan technique, using graphite-monochromated MoKα radiation (λ = 0.71073 Å), at 100(1) on Rigaku XCalibur (Rigaku OD, Neu-Isenburg, Germany) four-circle diffractometer with EOS CCD detector. The data were corrected for Lorentz-polarization as well as for absorption effects [75]. Precise unit-cell parameters were determined by a least-squares fit of the 6861 reflections of the highest intensity, chosen from the whole experiment. The structures were solved with SHELXT [76] and refined with the full-matrix least-squares procedure on F2 by SHELXL [77]. All non-hydrogen atoms were refined anisotropically. Hydrogen atoms were placed in idealized positions and refined as ‘riding model’ with isotropic displacement parameters set at 1.2 (1.5 for CH_3_) times Ueq of appropriate carrier atoms. 

Crystallographic data for the structural analysis has been deposited with the Cambridge Crystallographic Data Centre, no. CCDC–2045899. Copies of this information may be obtained free 589 of charge from: The Director, CCDC, 12 Union Road, Cambridge, CB2 1EZ, UK; e-mail: deposit@ccdc.cam.ac.uk, or www.ccdc.cam.ac.uk.

Crystal data. C_70_H_68_N_8_O_14_Si_10_, M_r_ = 1526.22, monoclinic, P2_1/c_, a = 14.8188(5) Å, b = 22.6566(9) Å, c = 11.1567(3) Å, β = 101.194(3)°, V = 3674.5(2) Å^3^, Z = 2, dx = 1.379 g·cm^−3^, F(000) = 1592, μ = 0.248 mm^−1^, 16,862 reflections collected, 6455 symmetry-independent (Rint = 2.40%), 5402 with I > 2σ(I). Final R(F) [I > 2σ(I)] = 0.0580, wR2 [I > 2σ(I) = = 0.1257, R(F) [all data] = 0.0709, wR2 [all data] = 0.1298, Δρmax/min = 1.398/−0.627 e/Å^−3^.

### 3.3. General Procedure for Copper(I)-Catalyzed Azide-Alkyne Cycloaddition (CuAAC)

#### 3.3.1. Synthetic Procedure with the Use of CuSO_4_ as Cu(II) Ion Source

The exemplary synthetic procedure is presented for **iBuT_8_-A1**. To a solution of **iBuT_8_-N3** (300 mg, 0.33 mmol) in THF (15 mL), sodium L-ascorbate crystalline (in general 0.3–5 eq., herein 5 eq.), **A1** (in general 1.4–8 eq., herein 7.85 eq.) and copper(II) sulfate pentahydrate (in general 0.025–0.25eq., herein 0.25 eq.) diluted in water, were added respectively. The reaction was conducted in a closed system until full conversion of **iBuT_8_-N3**, confirmed by FT-IR analysis (typically 72–96 h depending on alkyne used). The crude product was filtered off by column chromatography (silica gel 60, THF) to remove solid impurities and the solvent was evaporated. It was extracted with DCM and water. Organic layer was dried with anhydrous sodium sulfate. Then the solvent was removed under reduced pressure, and the product was precipitated in methanol as white solid. The product (83 mg, i.e., 78% isolated yield) was analyzed by ^1^H, ^13^C, and ^29^Si NMR and EA to confirm its structure. For the spectroscopic analysis please see Supplementary Materials.

#### 3.3.2. Synthetic Procedure with Use of CuBr as Cu(I) Ion Source

The exemplary synthetic procedure is presented for **iBuT_8_-A7** [33]. To a solution of **iBuT_8_-N3** (200 mg, 0,22 mmol) and **A7** (35 μL, 0.33 mmol) in THF (3 mL) stirring under argon, CuBr (3.2 mg, 0.02 mmol) and PMDTA (4.6 μL, 0.02 mmol) were added. The reaction mixture was stirred at room temperature for 30 h. After the reaction was completed (FT-IR analysis), the crude product was filtered off by column chromatography (silica gel 60, THF) to remove solid impurities and solvent was evaporated. The resulted solid was washed with methanol and dried in vacuo. Second option for isolation is the extraction in DCM and water. Organic layer was dried with sodium anhydrous sulfate, solvent was removed under reduced pressure, and product was precipitated in methanol as a pale yellow solid in 81% yield (181 mg). Product was analyzed by ^1^H, ^13^C, and ^29^Si NMR and EA to confirm its structure. 

### 3.4. General Procedure for Using SQs-Based Pyridyl-Triazole- and Thiophenyl-Triazole Derivatives as Ligands in the Formation of Coordination Complexes with Selected Transition Metals (Pd, Pt, Rh)

#### 3.4.1. Procedure for the Synthesis of **iBuT_8_-A7-Pd(N^S)**, **DDSQ-A1-[Pd(N^N)]_2_**, and **(iBuT_8_-A1)_2_-Rh(N^N)**

The procedure for the synthesis of **iBuT_8_-A7-Pd(N^S)** is described as an example.

A mixture of 1 equiv. of **iBuT8-A7** ligand and a stoichiometric amount of Pd(cod)Cl_2_ was dissolved in dichloromethane and stirred at room temperature for 24 h. After this time, a solvent was evaporated. The crude product was dissolved in hexane and filtrated off via cannula. The solvent was evaporated and afforded in pure **iBuT_8_-A7-Pd(N^S)** as yellow solid in 60% yield (91 mg). It was dried in vacuo. Complex **DDSQ-A1-[Pd(N^N)]_2_** was obtained analogously, however, it precipitated from the DCM solution. After 24 h, the solvent was evaporated and washed with hexane and dried in vacuo. Obtained products were yellow **DDSQ-A1-[Pd(N^N)]_2_** (93%, 228 mg) and orange **(iBuT_8_-A1)_2_-Rh(N^N)** (for Rh, the complexation was performed within 96 h) (55%, 70 mg) solids. **DDSQ-A1-[Pd(N^N)]_2_** exhibits very restricted solubility in DCM, chloroform, THF or hexane and is soluble in DMF and DMSO.

#### 3.4.2. Procedure for Synthesis of **iBuT_8_-A1-Pt(N^N)**

The complex was synthesized as described by Galanski and Keppler et al. with slight modifications [78]. To a solution of ligand **iBuT_8_-A1** (0.051 g, 0.05 mmol, 1.005 eq.) in THF, a solution of K_2_PtCl_4_ in water-MeOH (1:1) was added. The mixture was stirred overnight in a light-protected flask at 40 °C. After this time, the mixture was in a form of suspension and the addition of MeOH resulted in crude product precipitation. It was washed with methanol and afforded in pure **iBuT_8_-A1-Pt(N^N)** as pale pale-yellow solid in 87% yield (55 mg) and then dried in vacuo.

## 4. Conclusions

In conclusion, we reported on the synthesis and characterization of a series of T_8_- and DDSQ-based double-decker silsesquioxanes bearing 4-substituted triazole ring with aryl, hetaryl, alkyl, silyl, and germyl groups via copper(I)-catalyzed azide-alkyne cycloaddition (CuAAC). From this group of compounds hetaryl-triazole i.e., pyridine- and thiophenyl-derivatives were selected and verified in terms of their coordinating properties towards Pd(II), Pt(II), and Rh(I) ions. As a result of performed tests, four types of complexes, i.e., two mononuclear iBuT_8_-based Pt- and Pd- with N^N and N^S ligands, dinuclear iBuT_8_-based Rh with N^N ligand as well as DDSQ-based with two Pd ions coordinated with N^N bidentate ligand were obtained and fully characterized. For the **DDSQ-2A1** ligand, this is the first example of using the pyridine-triazole moiety to anchor TM ion in the chemistry of DDSQ-compounds. These may be potentially valuable systems of catalytic activity that will be tested in our future studies. 

## Figures and Tables

**Table 1:** Copper-catalyzed azide-alkyne cycloaddition using **iBuT_8_-N3** and alkynes.

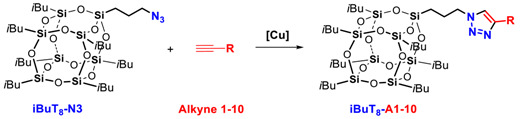					
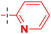 **iBuT_8_-A1** (78%) ^a^		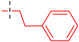 **iBuT_8_-A2** (90%) ^a^		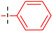 **iBuT_8_-A3** (83%) ^a^	
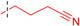 **iBuT_8_-A4** (82%) ^a^		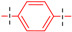 **iBuT_8_-A5** (90%) ^a^		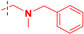 **iBuT_8_-A6** (89%) ^a^	
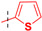 **iBuT_8_-A7** (81%) ^a^	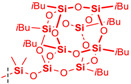 **iBuT_8_-A8** (84%) ^b^		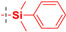 **iBuT_8_-A9** (70%) ^b^		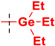 **iBuT_8_-A10** (85%) ^c^

^a^ Reaction conditions for Cu(II) source: [azide]:[alkyne]:[CuSO_4_][sodium ascorbate] = 1:1.4–8:0.025–0.25:0.3–5; 25–60 °C; 72–96 h. ^b^ Reaction conditions for Cu(I) source: [azide]:[alkyne]:[CuBr][PMDTA] = 1:1.45:0.1:0.1; 25 °C; 24 h. ^c^ additional 12 h at 45 °C. > 99% conversion of **iBuT_8_-N3** was confirmed by FT-IR in situ and ^1^H-NMR analyses. Value in parenthesis is given for isolation yield (%).

**Table 2:** Copper-catalyzed azide-alkyne cycloaddition using **DDSQ-2N3** ^a^.

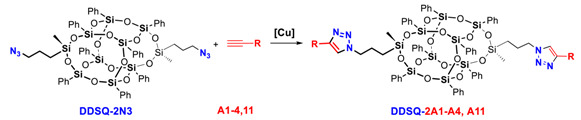			
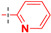 **DDSQ-2A1** (85%) ^a^	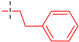 **DDSQ-2A2** (60%) ^a^		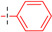 **DDSQ-2A3** (82%) ^a^
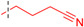 **DDSQ-2A4** (78%) ^a^		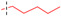 **DDSQ-2A11** (84%) ^a^	

^a^ Reaction conditions for Cu(II) source: [azide]:[alkyne]:[CuSO_4_][sodium ascorbate] = 1:1.4–8:0.025–0.25:0.3–5; 25–60 °C; 72–96 h. > 99% conversion of **DDSQ-2N3** was confirmed by FT-IR in situ and ^1^H-NMR analyses. Value in parenthesis is given for isolation yield (%).

## Data Availability

The data presented in this study are available in this article or in a Supplementary Materials.

## Supplementary Materials

The following are available online, ^1^H, ^13^C, ^29^Si NMR spectra of all obtained compounds (Figures S1–S53).

## Sample Availability

Not available.

## References

[1] Hartmann-Thompson C. (2011). Applications of Polyhedral Oligomeric Silsesquioxanes.

[2] Du Y., Liu H. (2020). Cage-like Silsesquioxanes-based Hybrid Materials. Dalt. Trans..

[3] Dong F., Lu L., Ha C.S. (2019). Silsesquioxane-Containing Hybrid Nanomaterials: Fascinating Platforms for Advanced Applications. Macromol. Chem. Phys..

[4] John Ł. (2018). Selected developments and medical applications of organic–inorganic hybrid biomaterials based on functionalized spherosilicates. Mater. Sci. Eng. C.

[5] Cordes D.B., Lickiss P.D., Rataboul F. (2010). Recent developments in the chemistry of cubic polyhedral oligosilsesquioxanes. Chem. Rev..

[6] Ahmed N., Fan H., Dubois P., Zhang X., Fahad S., Aziz T., Wan J. (2019). Nano-engineering and micromolecular science of polysilsesquioxane materials and their emerging applications. J. Mater. Chem. A.

[7] Kaźmierczak J., Kuciński K., Hreczycho G. (2017). Highly Efficient Catalytic Route for the Synthesis of Functionalized Silsesquioxanes. Inorg. Chem..

[8] Dudziec B., Zak P., Marciniec B. (2019). Synthetic routes to silsesquioxane-based systems as photoactive materials and their precursors. Polymers.

[9] Brick C.M., Ouchi Y., Chujo Y., Laine R.M. (2005). Robust Polyaromatic Octasilsesquioxanes from Polybromophenylsilsesquioxanes, Br x OPS, via Suzuki Coupling. Macromolecules.

[10] Walczak M., Januszewski R., Dutkiewicz M., Franczyk A., Marciniec B. (2019). A facile approach for the synthesis of novel silsesquioxanes with mixed functional groups. New J. Chem..

[11] Żak P., Bołt M., Grzelak M., Rachuta K., Dudziec B., Januszewski R., Marciniec B., Marciniak B. (2020). Synthesis and properties of chromophore-functionalized monovinylsilsesquioxane derivatives. New J. Chem..

[12] Grzelak M., Frąckowiak D., Januszewski R., Marciniec B. (2020). Introduction of organogermyl functionalities to cage silsesquioxanes. Dalt. Trans..

[13] Kaźmierczak J., Hreczycho G. (2019). Copper(II) triflate-mediated synthesis of functionalized silsesquioxanes via dehydrogenative coupling of POSS silanols with hydrosilanes. Dalt. Trans..

[14] Jung J.H., Furgal J.C., Goodson T., Mizumo T., Schwartz M., Chou K., Laine R.M. (2012). 3-D Molecular Mixtures of Catalytically Functionalized [vinylSiO1.5]10/[vinylSiO1.5]12. Photophysical Characterization of Second Generation Derivatives. Chem. Mater..

[15] Vautravers N.R., André P., Slawin A.M.Z., Cole-Hamilton D.J. (2009). Synthesis and characterization of photoluminescent vinylbiphenyl decorated polyhedral oligomeric silsesquioxanes. Org. Biomol. Chem..

[16] Żak P., Dudziec B., Kubicki M., Marciniec B. (2014). Silylative Coupling versus Metathesis-Efficient Methods for the Synthesis of Difunctionalized Double-Decker Silsesquioxane Derivatives. Chem. A Eur. J..

[17] Asuncion M.Z., Roll M.F., Laine R.M. (2008). Octaalkynylsilsesquioxanes, Nano Sea Urchin Molecular Building Blocks for 3-D-Nanostructures. Macromolecules.

[18] Araki H., Naka K. (2012). Syntheses and properties of dumbbell-shaped POSS derivatives linked by luminescent Pi-conjugated units. J. Polym. Sci. Part A Polym. Chem..

[19] Cho H.J., Hwang D.H., Lee J.I.J., Jung Y.K., Park J.H., Lee J.I.J., Lee S.K., Shim H.K. (2006). Electroluminescent polyhedral oligomeric silsesquioxane-based nanoparticle. Chem. Mater..

[20] Guan J., Arias J.J.R., Tomobe K., Ansari R., Marques M. de F.V., Rebane A., Mahbub S., Furgal J.C., Yodsin N., Jungsuttiwong S. (2020). Unconventional Conjugation via vinylMeSi(O−)2 Siloxane Bridges May Imbue Semiconducting Properties in [vinyl(Me)SiO(PhSiO 1.5 ) 8 OSi(Me)vinyl-Ar] Double-Decker Copolymers. ACS Appl. Polym. Mater..

[21] Hein J.E., Fokin V.V. (2010). Copper-catalyzed azide-alkyne cycloaddition (CuAAC) and beyond: new reactivity of copper(I) acetylides. Chem. Soc. Rev..

[22] Ma J., Ding S. (2002). Transition Metal-Catalyzed Cycloaddition of Azides with Internal Alkynes. Asian J. Org. Chem..

[23] Huo J., Lin C., Liang J. (2020). A brief minireview of poly-triazole: Alkyne and azide substrate selective, metal-catalyst expansion. React. Funct. Polym..

[24] Rostovtsev V.V., Green L.G., Fokin V.V., Sharpless K.B. (2002). A stepwise huisgen cycloaddition process: Copper(I)-catalyzed regioselective “ligation” of azides and terminal alkynes. Angew. Chem. Int. Ed..

[25] Tornøe C.W., Christensen C., Meldal M. (2002). Peptidotriazoles on solid phase: [1,2,3]-Triazoles by regiospecific copper(I)-catalyzed 1,3-dipolar cycloadditions of terminal alkynes to azides. J. Org. Chem..

[26] Singh M.S., Chowdhury S., Koley S. (2016). Advances of azide-alkyne cycloaddition-click chemistry over the recent decade. Tetrahedron.

[27] Liang L., Astruc D. (2011). The copper(I)-catalyzed alkyne-azide cycloaddition (CuAAC) “click” reaction and its applications. An overview. Coord. Chem. Rev..

[28] Sindhu K.S., Anilkumar G. (2014). Recent advances and applications of Glaser coupling employing greener protocols. RSC Adv..

[29] Ervithayasuporn V., Abe J., Wang X., Matsushima T., Murata H., Kawakami Y. (2010). Synthesis, characterization, and OLED application of oligo(p-phenylene ethynylene)s with polyhedral oligomeric silsesquioxanes (POSS) as pendant groups. Tetrahedron.

[30] Wang X., Ervithayasuporn V., Zhang Y., Kawakami Y. (2011). Reversible self-assembly of dendrimer based on polyhedral oligomeric silsesquioxanes (POSS). Chem. Commun..

[31] Han J., Zheng Y., Zheng S., Li S., Hu T., Tang A., Gao C. (2014). Water soluble octa-functionalized POSS: All-click chemistry synthesis and efficient host–guest encapsulation. Chem. Commun..

[32] Zhou Y., Yang G., Lu C., Nie J., Chen Z., Ren J. (2016). POSS supported C2-symmetric bisprolinamide as a recyclable chiral catalyst for asymmetric Aldol reaction. Catal. Commun..

[33] Zheng W., Lu C., Yang G., Chen Z., Nie J. (2015). POSS supported diarylprolinol silyl ether as an efficient and recyclable organocatalyst for asymmetric Michael addition reactions. Catal. Commun..

[34] Zhu Y.K., Guang S.Y., Xu H.Y. (2012). A versatile nanobuilding precursor for the effective architecture of well-defined organic/inorganic hybrid via click chemistry. Chin. Chem. Lett..

[35] Pu Y.J., Yuan H., Yang M., He B., Gu Z.W. (2013). Synthesis of peptide dendrimers with polyhedral oligomeric silsesquioxane cores via click chemistry. Chin. Chem. Lett..

[36] Schäfer S., Kickelbick G. (2017). Simple and high yield access to octafunctional azido, amine and urea group bearing cubic spherosilicates. Dalt. Trans..

[37] Ak M., Gacal B., Kiskan B., Yagci Y., Toppare L. (2008). Enhancing electrochromic properties of polypyrrole by silsesquioxane nanocages. Polymer (Guildf)..

[38] Ervithayasuporn V., Wang X., Gacal B., Gacal B.N., Yagci Y., Kawakami Y. (2011). Formation of trimethylsilylated open-cage oligomeric azidophenylsilsesquioxanes. J. Organomet. Chem..

[39] Wei K., Wang L., Zheng S. (2013). Organic-inorganic copolymers with double-decker silsesquioxane in the main chains by polymerization via click chemistry. J. Polym. Sci. Part A Polym. Chem..

[40] Liu Y., Kigure M., Koizumi K., Takeda N., Unno M., Ouali A. (2020). Synthesis of Tetrachloro, Tetraiodo, and Tetraazido Double-Decker Siloxanes. Inorg. Chem..

[41] Nowacka M., Makowski T., Kowalewska A. (2020). Hybrid fluorescent poly(Silsesquioxanes) with amide-and triazole-containing side groups for light harvesting and cation sensing. Materials.

[42] Li Y., Dong X.H., Zou Y., Wang Z., Yue K., Huang M., Liu H., Feng X., Lin Z., Zhang W. (2017). Polyhedral oligomeric silsesquioxane meets “click” chemistry: Rational design and facile preparation of functional hybrid materials. Polymer (Guildf)..

[43] Pérez-Ojeda M.E., Trastoy B., Lõpez-Arbeloa Í., Bañuelos J., Costela Ú., García-Moreno I., Chiara J.L. (2011). Click Assembly of Dye-Functionalized Octasilsesquioxanes for Highly Efficient and Photostable Photonic Systems. Chem. A Eur. J..

[44] Sekiya R., Uemura Y., Naito H., Naka K., Haino T. (2016). Chemical Functionalisation and Photoluminescence of Graphene Quantum Dots. Chem. A Eur. J..

[45] Zhao G., Zhu Y., Guang S., Ke F., Xu H. (2018). Facile preparation and investigation of the properties of single molecular POSS-based white-light-emitting hybrid materials using click chemistry. New J. Chem..

[46] Namvari M., Du L., Stadler F.J. (2017). Graphene oxide-based silsesquioxane-crosslinked networks-synthesis and rheological behavior. RSC Adv..

[47] Gungor E., Bilir C., Hizal G., Tunca U. (2010). Multiarm Star Polymers with POSS at the Periphery EDA. J. Polym. Sci. Part A Polym. Chem..

[48] Arslan I., Tasdelen M.A. (2016). POSS-based hybrid thermosets via photoinduced copper-catalyzed azide-alkyne cycloaddition click chemistry. Des. Monomers Polym..

[49] Niu M., Li T., Xu R., Gu X., Yu D., Wu Y. (2013). Synthesis of PS-g-POSS hybrid graft copolymer by click coupling via “graft onto” strategy. J. Appl. Polym. Sci..

[50] Uner A., Doganci E., Tasdelen M.A. (2018). Non-covalent interactions of pyrene end-labeled star poly(ɛ-caprolactone)s with fullerene. J. Appl. Polym. Sci..

[51] Bach L.G., Islam M.R., Nga T.T., Binh M.T., Hong S.S., Gal Y.S., Lim K.T. (2013). Chemical modification of polyhedral oligomeric silsesquioxanes by functional polymer via azide-alkyne click reaction. J. Nanosci. Nanotechnol..

[52] Li L., Zhang C., Zheng S. (2017). Synthesis of POSS-terminated polycyclooctadiene telechelics via ring-opening metathesis polymerization. J. Polym. Sci. Part A Polym. Chem..

[53] Gauthier M., Aridi T. (2019). Synthesis of arborescent polystyrene by “click” grafting. J. Polym. Sci. Part A Polym. Chem..

[54] Chang P., Xu S., Zhao B., Zheng S. (2019). A design of shape memory networks of poly(ε-caprolactone)s via POSS-POSS interactions. Polym. Adv. Technol..

[55] Wang Z., Li Y., Dong X.H., Yu X., Guo K., Su H., Yue K., Wesdemiotis C., Cheng S.Z.D., Zhang W. (2013). Bin Giant gemini surfactants based on polystyrene-hydrophilic polyhedral oligomeric silsesquioxane shape amphiphiles: Sequential “click” chemistry and solution self-assembly. Chem. Sci..

[56] Yue K., Liu C., Guo K., Yu X., Huang M., Li Y., Wesdemiotis C. (2012). Sequential “ Click ” Approach to Polyhedral Oligomeric Silsesquioxane-Based Shape Amphiphiles. Macromolecules.

[57] Trastoy B., Eugenia Pérez-Ojeda M., Sastre R., Chiara J.L. (2010). Octakis(3-azidopropyl)octasilsesquioxane: A versatile nanobuilding block for the efficient preparation of highly functionalized cube-octameric polyhedral oligosilsesquioxane frameworks through click assembly. Chem. A Eur. J..

[58] Pérez-Ojeda M.E., Trastoy B., Rol Á., Chiara M.D., García-Moreno I., Chiara J.L. (2013). Controlled click-assembly of well-defined hetero-bifunctional cubic silsesquioxanes and their application in targeted bioimaging. Chem. A Eur. J..

[59] Ervithayasuporn V., Kwanplod K., Boonmak J., Youngme S., Sangtrirutnugul P. (2015). Homogeneous and heterogeneous catalysts of organopalladium functionalized-polyhedral oligomeric silsesquioxanes for Suzuki-Miyaura reaction. J. Catal..

[60] Ervithayasuporn V., Wang X., Kawakami Y. (2009). Synthesis and characterization of highly pure azido-functionalized polyhedral oligomeric silsesquioxanes (POSS). Chem. Commun..

[61] Vogelsang D.F., Dannatt J.E., Maleczka R.E., Lee A. (2018). Separation of asymmetrically capped double-decker silsesquioxanes mixtures. Polyhedron.

[62] Demko Z.P., Sharpless K.B. (2001). Preparation of 5-substituted 1H-tetrazoles from nitriles in water. J. Org. Chem..

[63] Cuevas F., Oliva A.I., Pericàs M.A. (2010). Direct copper(I)-catalyzed cycloaddition of organic azides with TMS-protected alkynes. Synlett.

[64] Demina M.M., Nguyen T.L.H., Shaglaeva N.S., Mareev A.V., Medvedeva A.S. (2012). Highly efficient synthesis of 4-trialkylsilyl(germyl)-1H-1,2,3-triazole-5-carbaldehydes. Russ. J. Org. Chem..

[65] Ziarani G.M., Hassanzadeh Z., Gholamzadeh P., Asadi S., Badiei A. (2016). Advances in Click Chemistry for the Silica based Material Construction. RSC Adv..

[66] Walczak M., Januszewski R., Majchrzak M., Kubicki M., Dudziec B., Marciniec B. (2017). The unusual cis- and trans-architecture of dihydrofunctional double-decker shaped silsesquioxane—Design and construction of its ethyl bridged π-conjugated arene derivatives. New J. Chem..

[67] Gavezzotti A., Filippini G. (1994). Geometry of the intermolecular X-H⋯Y (X, Y = N, O) hydrogen bond and the calibration of empirical hydrogen-bond potentials. J. Phys. Chem..

[68] Gavezzotti A. (1994). Are crystal structures predictable?. Acc. Chem. Res..

[69] Piec K., Kostera S., Jędrzkiewicz D., Ejfler J., John Ł. (2020). Mono-substituted amine-oligosilsesquioxanes as functional tools in Pd(II) coordination chemistry: synthesis and properties. New J. Chem..

[70] Marciniec B., Kownacki I., Franczyk A., Kubicki M. (2011). Silsesquioxyl rhodium(i) complexes—Synthesis, structure and catalytic activity. Dalt. Trans..

[71] Au-Yeung H.-L., Leung S.Y.-L., Yam V.W.-W. (2018). Supramolecular assemblies of dinuclear alkynylplatinum(II) terpyridine complexes with double-decker silsesquioxane nano-cores: the role of isomerism in constructing nano-structures. Chem. Commun..

[72] Kucuk A.C., Matsui J., Miyashita T. (2018). Synthesis and photochemical response of Ru(II)-coordinated double-decker silsesquioxane. RSC Adv..

[73] Mei Y., Loth M.A., Payne M., Zhang W., Smith J., Day C.S., Parkin S.R., Heeney M., McCulloch I., Anthopoulos T.D. (2013). High mobility field-effect transistors with versatile processing from a small-molecule organic semiconductor. Adv. Mater..

[74] Dudziec B., Rzonsowska M., Marciniec B., Brząkalski D., Woźniak B. (2014). New mono- and diethynylsiloxysilsesquioxanes—Efficient procedures for their synthesis. Dalt. Trans..

[75] Rigaku Oxford Diffraction (2020). CrysAlisPro v1.171.40.81a.

[76] Sheldrick G.M. (2015). SHELXT—Integrated space-group and crystal-structure determination. Acta Crystallogr. Sect. A Found. Crystallogr..

[77] Sheldrick G.M. (2015). Crystal structure refinement with SHELXL. Acta Crystallogr. Sect. C Struct. Chem..

[78] Sommerfeld N.S., Gülzow J., Roller A., Cseh K., Jakupec M.A., Grohmann A., Galanski M., Keppler B.K. (2017). Antiproliferative Copper(II) and Platinum(II) Complexes with Bidentate N,N-Donor Ligands. Eur. J. Inorg. Chem..

