# Supplementary material for: Synthesis of Silsesquioxanes with Substituted Triazole Ring Functionalities and Their Coordination Ability
Source: Molecules. 2021 Jan 15;26(2):439. doi: 10.3390/molecules26020439 (PMC7830482; doi:10.3390/molecules26020439)
Supplement: Supplementary file 1 [file molecules-26-00439-s001.pdf]

Supporting information for:

Synthesis of Silsesquioxanes with Substituted Triazole Ring Functionalities and Their Coordination Ability‡

Monika Rzonsowska [1,2,*], Katarzyna Kozakiewicz [1], Katarzyna Mituła [1,2], Julia Duszczak [1,2], Maciej Kubicki [3], Beata Dudziec [1,2,*]

[1] Department of Organometallic Chemistry, Faculty of Chemistry, Adam Mickiewicz University in Poznań, Uniwersytetu Poznańskiego 8, 61-614 Poznań, Poland
[2] Centre for Advanced Technologies, Adam Mickiewicz University in Poznań, Uniwersytetu Poznańskiego 10, 61-614 Poznań, Poland
[3] Faculty of Chemistry, Adam Mickiewicz University in Poznań, Uniwersytetu Poznańskiego 8, 61-614 Poznań, Poland
* Correspondence: mrzons@amu.edu.pl (M. R.); beata.dudziec@gmail.com (B.D.)
‡Dedicated to Professor Julian Chojnowski on the occasion of his 85th birthday.

Table of content

Spectra of obtained products:	S-2
iBuT$_8$-A1	S-2
iBuT$_8$-A2	S-4
iBuT$_8$-A3	S-6
iBuT$_8$-A4	S-8
iBuT$_8$-A5	S-9
iBuT$_8$-A6	S-11
iBuT$_8$-A7	S-13
iBuT$_8$-A8	S-15
iBuT$_8$-A9	S-17
iBuT$_8$-A10	S-19
DDSQ-2A1	S-21
DDSQ-2A2	S-23
DDSQ-2-A3	S-24
DDSQ-2-A4	S-26
DDSQ-2-A11	S-27
Spectra of obtained complexes:	S-29
(iBuT$_8$-A1)$_2$-Rh(N^N)	S-29
iBuT$_8$-A1-Pt(N^N)	S-31
iBuT$_8$-A1-Pd(N^S)	S-33
DDSQ-A1-[Pd(N^N)]$_2$	S-35
Comparison of the ^{1}H NMR spectra of ligand iBuT$_8$-A1 and complex iBuT$_8$-A1-Pt(N^N)	S-37
Comparison of the ^{1}H NMR spectra of ligand iBuT$_8$-A7 and complex iBuT$_8$-A7-Pd(N^S)	S-37
References	S-38

Spectra of obtained products:

iBuT$_8$-A1

White solid, 78%

^{1}H NMR (300 MHz, $CDCl_3$, 25 °C) δ = 8.58 (d, 1H, $J_{H\text{-}H}$ = 4.1 Hz, PyH), 8.19 (d, 1H, $J_{H\text{-}H}$ = 7.9 Hz, PyH), 8.12 (s, 1H, NCH), 7.78 (td, 1H, $J_{H\text{-}H}$ = 7.7, 1.8 Hz, PyH), 7.23-7.20 (m, 1H, PyH), 4.40 (t, 2H, $J_{H\text{-}H}$ = 7.2 Hz, N-CH_2), 2.11-2.01 (m, 2H, $CH_2CH_2CH_2$), 1.92 (sext, 7H, $CH(CH_3)_2$), 0.96-0.93 (m, 42H, $CH(CH_3)_2$), 0.65 (overlapped, 2H, CH_2Si), 0.62-0.59 (m, 14H, $CH_2CH(CH_3)_2$); ^{13}C NMR (101 MHz, $CDCl_3$, 25 °C) δ = 150.59, 149.51, 148.49, 137.03, 122.92, 121.9, 120.35, 52.80, 25.85, 25.81, 24.03, 23.98, 22.68, 22.61, 22.56, 9.41; ^{29}Si NMR (79.5 MHz, $CDCl_3$, 25 °C) δ = -67.52, -67.87, -67.91, -68.62; IR (cm^{-1}): 2952.14, 2868.04, 1463.36, 1229.26, 1092.96, 741.26, 479.68; EA: Anal. calcd for $C_{38}H_{74}N_4O_{12}Si_8$ (%): C, 45.47, H, 7.43; found: C, 45.51; H, 7.49. Data consistent with the literature [1].

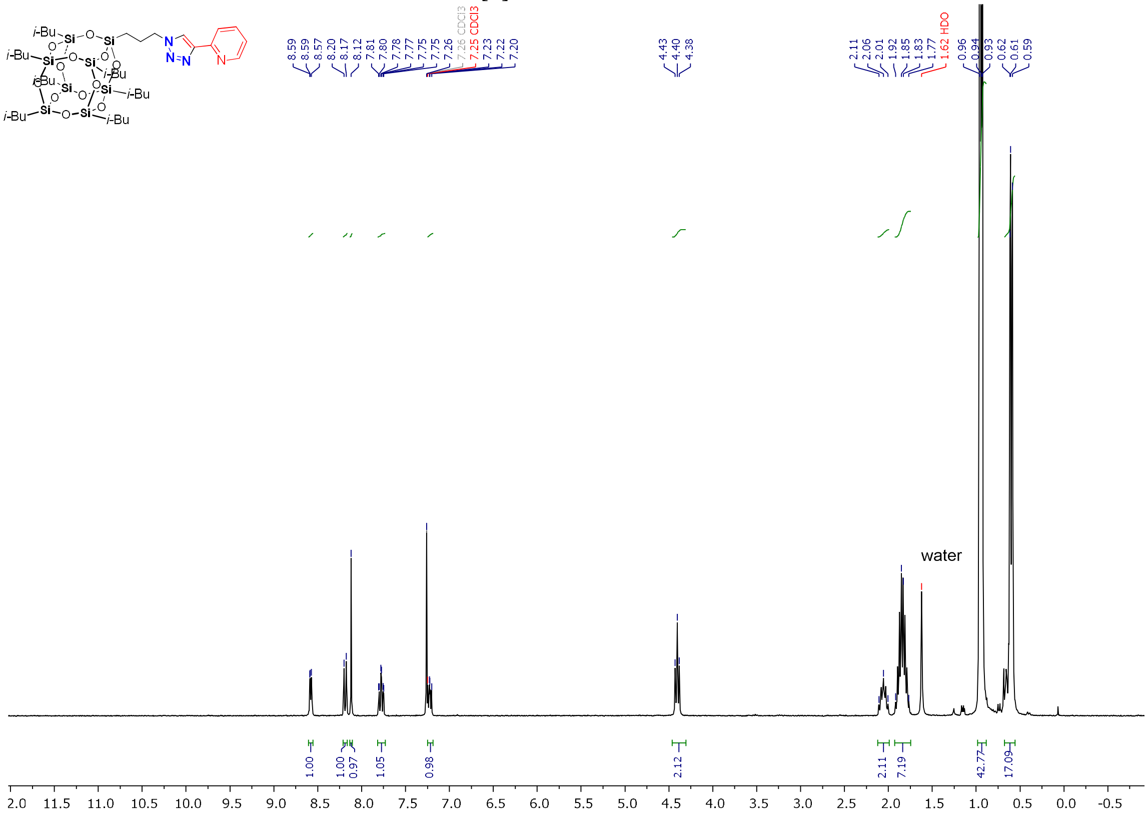

Figure S1 ^{1}H NMR spectra of iBuT$_8$-A1

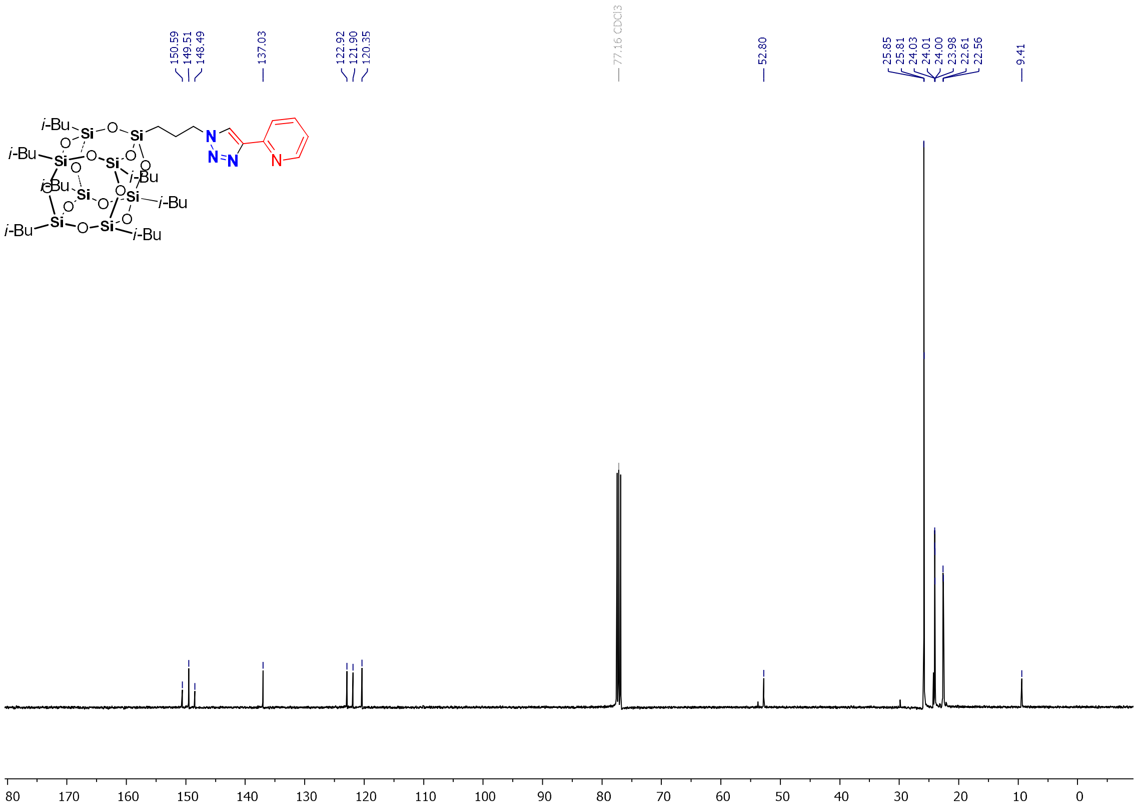

Figure S2 ^{13}C NMR spectra of iBuT$_8$-A1

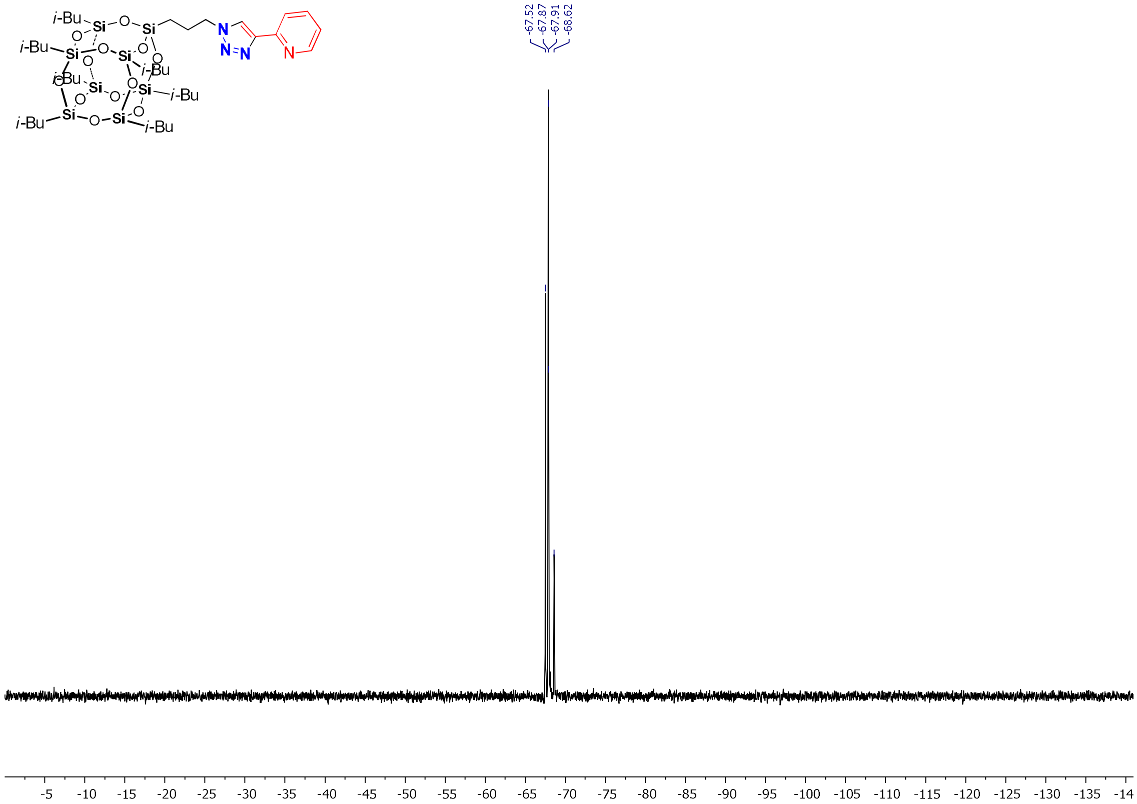

Figure S3 ^{29}Si NMR spectra of iBuT$_8$-A1

iBuT$_8$-A2

White solid, 90%

^{1}H NMR (300 MHz, $CDCl_3$, 25 °C) δ = 7.28-7.18 (m, 5H, Ph), 7.05 (s, 1H, NCH), 4.27 (t, 2H, $J_{H\text{-}H}$ = 7.1 Hz, N-CH_2), 3.02 (s, 2H, CH_2CH_2Ph), 1.97-1.92 (m, 2H, $CH_2CH_2CH_2$), 1.85-1.79 (m, 7H, $CH(CH_3)_2$), 0.95 (d, 42H, $J_{H\text{-}H}$ = 6.6 Hz, $CH(CH_3)_2$), 0.62 (overlapped, 2H, CH_2Si), 0.59 (m, 14H, $CH_2CH(CH_3)_2$); ^{13}C NMR (101 MHz, $CDCl_3$, 25 °C) δ = 141.30, 128.61, 128.52, 128.48, 126.20, 68.08, 52.49, 35.70, 25.81, 24.00, 23.96, 22.55, 22.52, 9.29; ^{29}Si NMR (79.5 MHz, $CDCl_3$, 25 °C) δ = -67.53, -67.81, -67.87, -68.44. IR (cm^{-1}): 2952.21, 2925.99, 2906.04, 2868.59, 1463.65, 1228.67, 1081.50, 739.59, 471.09; EA: Anal. calcd for $C_{41}H_{79}N_3O_{12}Si_8$ (%): C, 47.77, H, 7.73; found: C, 47.79; H, 7.85.

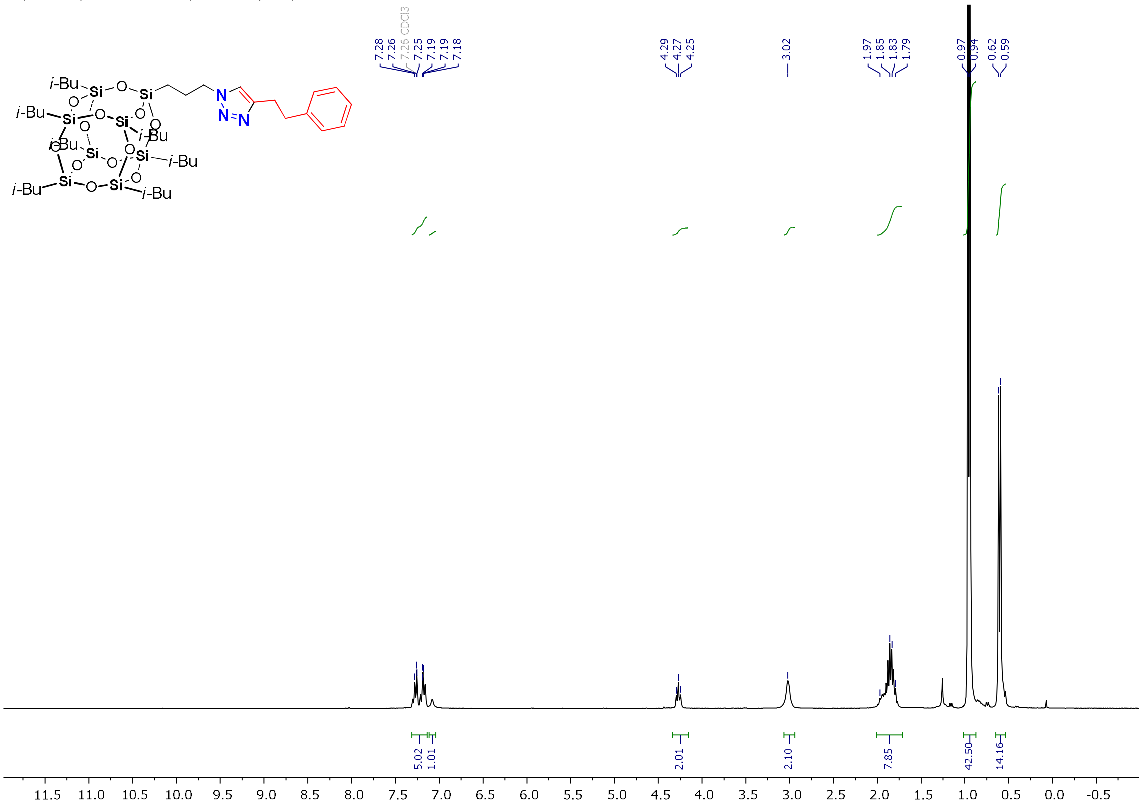

Figure S4 ^{1}H NMR spectra of iBuT$_8$-A2

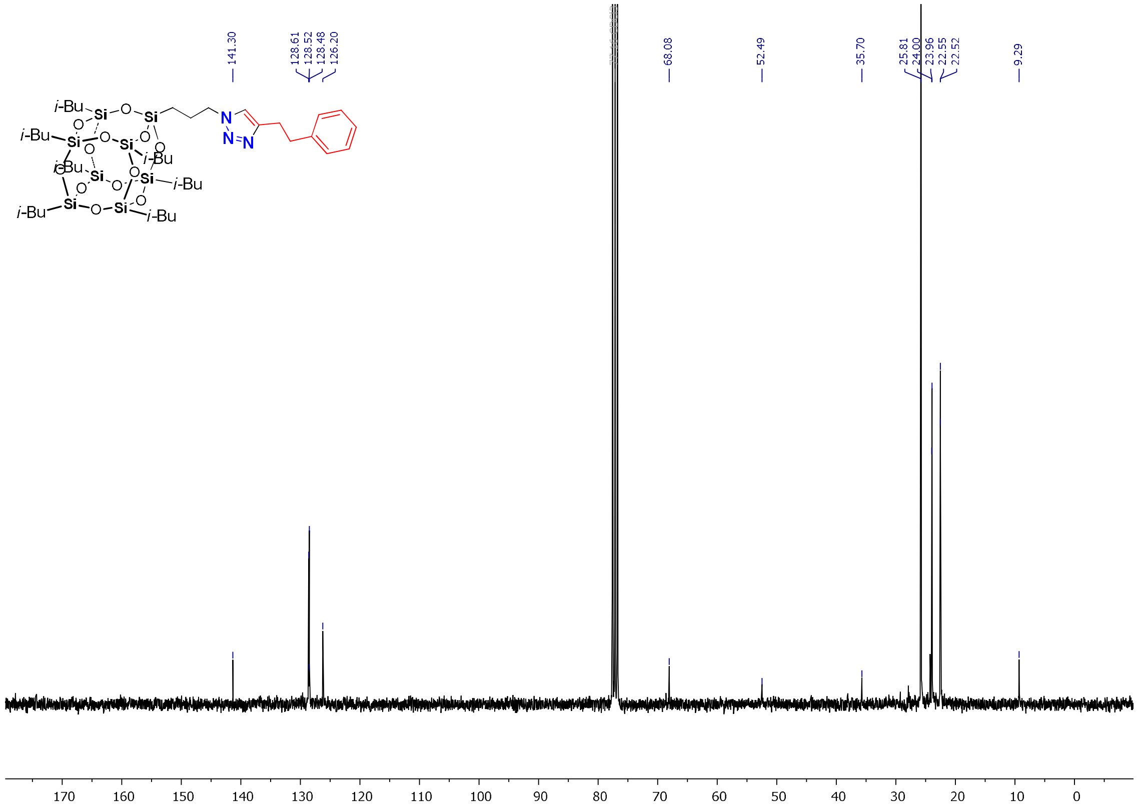

Figure S5 ^{13}C NMR spectra of iBuT$_8$-A2

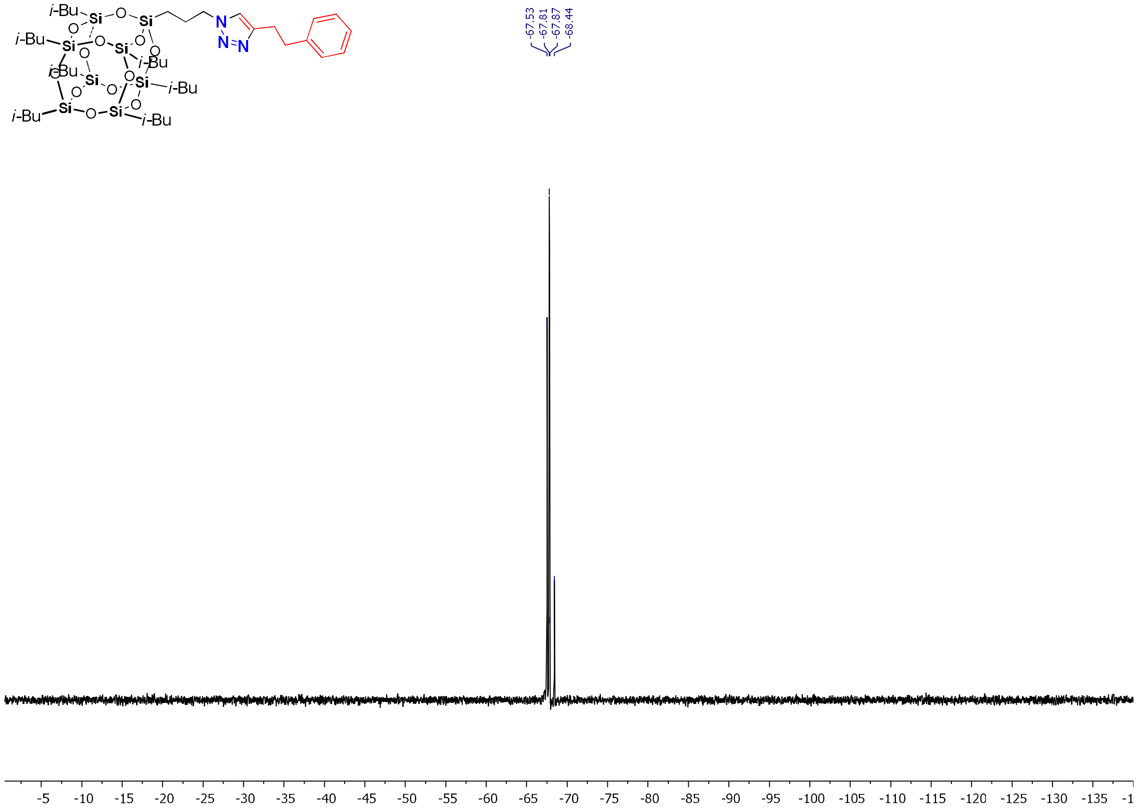

Figure S6 ^{29}Si NMR spectra of iBuT$_8$-A2

iBuT$_8$-A3

White solid, 83%

^{1}H NMR (400 MHz, $CDCl_3$, 25 °C) δ = 7.84 (m, 2H, J_{H-H} = 1.4 Hz, PhH), 7.72 (s, 1H, NCH), 7.45-7.41 (m, 2H, PhH), 7.34 (d, J_{H-H} = 7.4 Hz, 1H, PhH), 4.39 (t, J_{H-H} = 7.2 Hz 2H, N-CH_2), 2.05 (q, 2H, $CH_2CH_2CH_2$), 1.84 (m, 7H, $CH(CH_3)_2$), 0.95 (d, JH-H = 6.6 Hz, 42H, $CH(CH_3)_2$), 0.60 (dd, 14H, J_{H-H} = 7.0, 3.4 Hz, $CH_2CH(CH_3)_2$), 0.57 (overlapped, 2H, CH_2Si); ^{13}C NMR (101 MHz, $CDCl_3$, 25 °C) δ = 147.88, 130.88, 128.97, 128.22, 125.82, 119.38, 52.65, 29.86, 25.83, 24.36, 24.00 22.60, 9.38; ^{29}Si NMR (79.5 MHz, $CDCl_3$, 25 °C) δ = -67.54, -67.58, -67.85, -67.87, -67.91 -68.55; IR (cm^{-1}): 2953.75, 2925.62, 2868.91, 2098.11, 1464.52, 1264.41, 1228.27, 1090.54, 735.82, 476.42; EA: Anal. calcd for $C_{39}H_{75}N_3O_{12}Si_8$ (%): C, 46.72, H, 7.54; found: C, 46.81; H, 7.73.

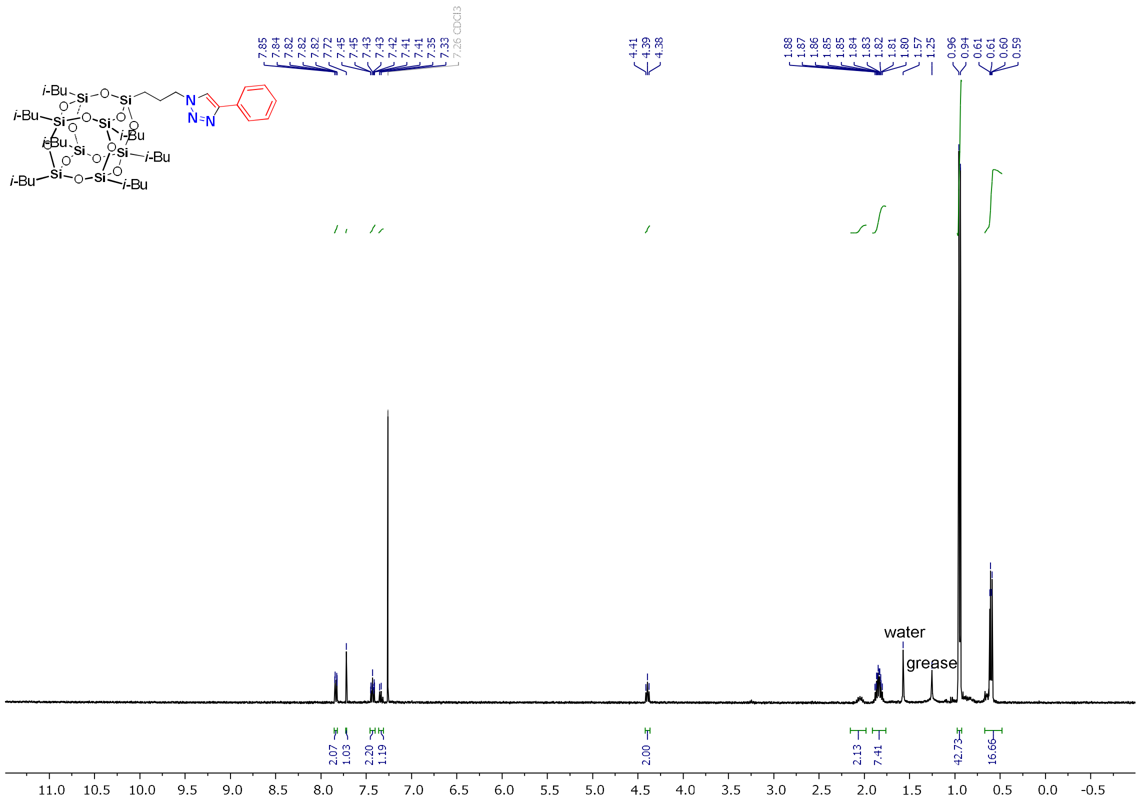

Figure S7 ^{1}H NMR spectra of iBuT$_8$-A3

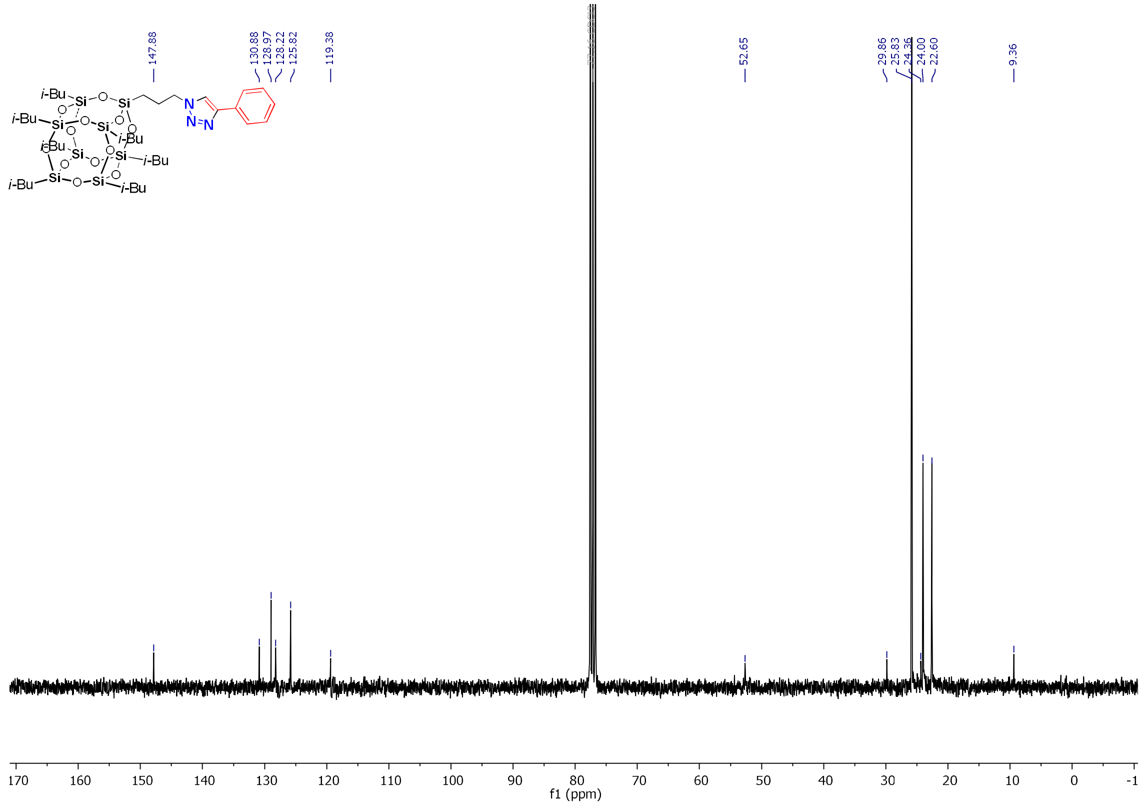

Figure S8 ^{13}C NMR spectra of iBuT$_8$-A3

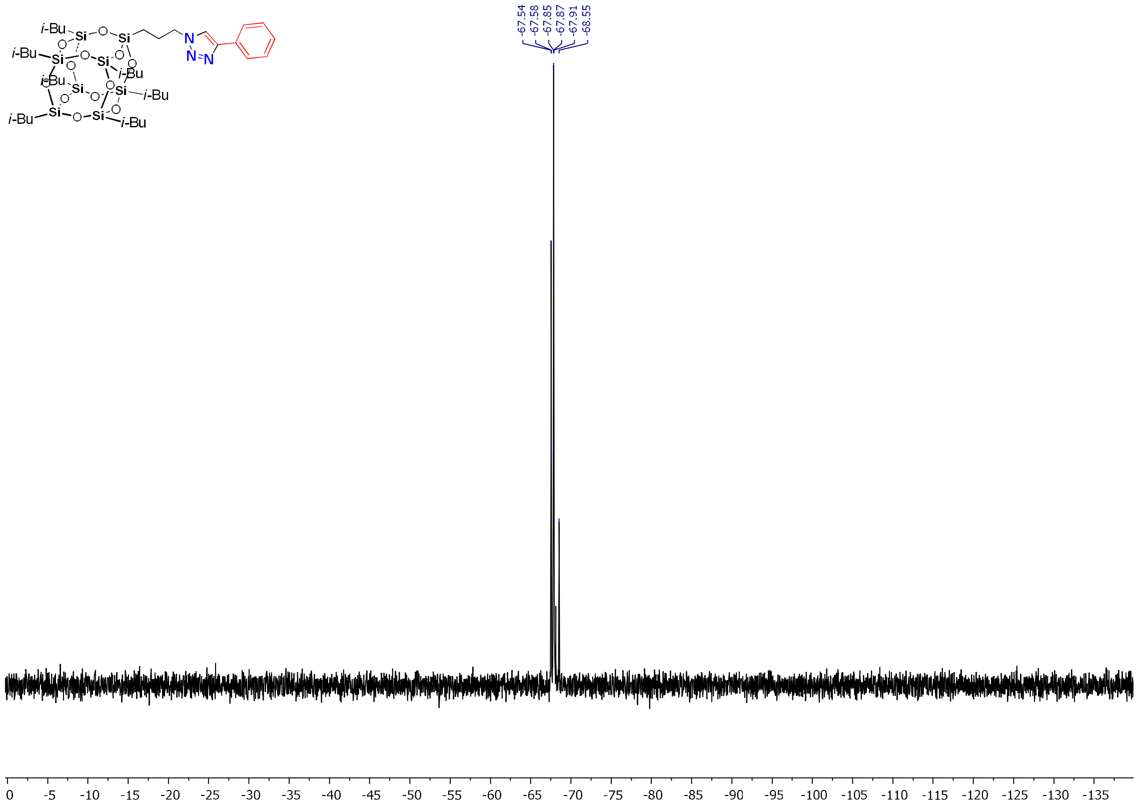

Figure S9 ^{29}Si NMR spectra of iBuT$_8$-A3

iBuT$_8$-A4

White solid, 82%

^{1}H NMR (300 MHz, CDCl3, 25 °C) δ = 7.30 (s, 1H, NCH), 4.30 (t, 2H, J_{H-H} = 7.2Hz, N-CH_2), 2.88 (t, 2H, J_{H-H} = 7.3Hz, $CH_2(CH_2)_2CN$), 2.43 (t, 2H, J_{H-H} = 7.1Hz, CH_2CH_2CN), 2.08 (t, 2H, J_{H-H} = 7.2Hz, CH_2CN), 1.84 (m, 7H, $CH(CH_3)_2$), 0.94 (d, 42H, J_{H-H} = 6.6 Hz, $CH(CH_3)_2$), 0.60 (overlapped, 2H, J_{H-H} = 1.2Hz, CH_2Si), 0.58 (d, 14H, J_{H-H} = 1.2Hz ,$CH_2CH(CH_3)_2$); ^{13}C NMR (101 MHz, $CDCl_3$, 25 °C) δ = 145.55, 121.09, 119.48, 52.55, 25.82, 25.80, 25.03, 24.32, 24.01, 23.97, 22.64, 22.57, 22.53, 16.64, 9.39; IR (cm^{-1}): 2952.09, 2868.29, 1463.91, 1229.24, 1092.4, 740.71, 479.83; EA: Anal. calcd for $C_{37}H_{76}N_4O_{12}Si_8$ (%): C, 44.72, H, 7.71; found: C, 44.85; H, 7.83.

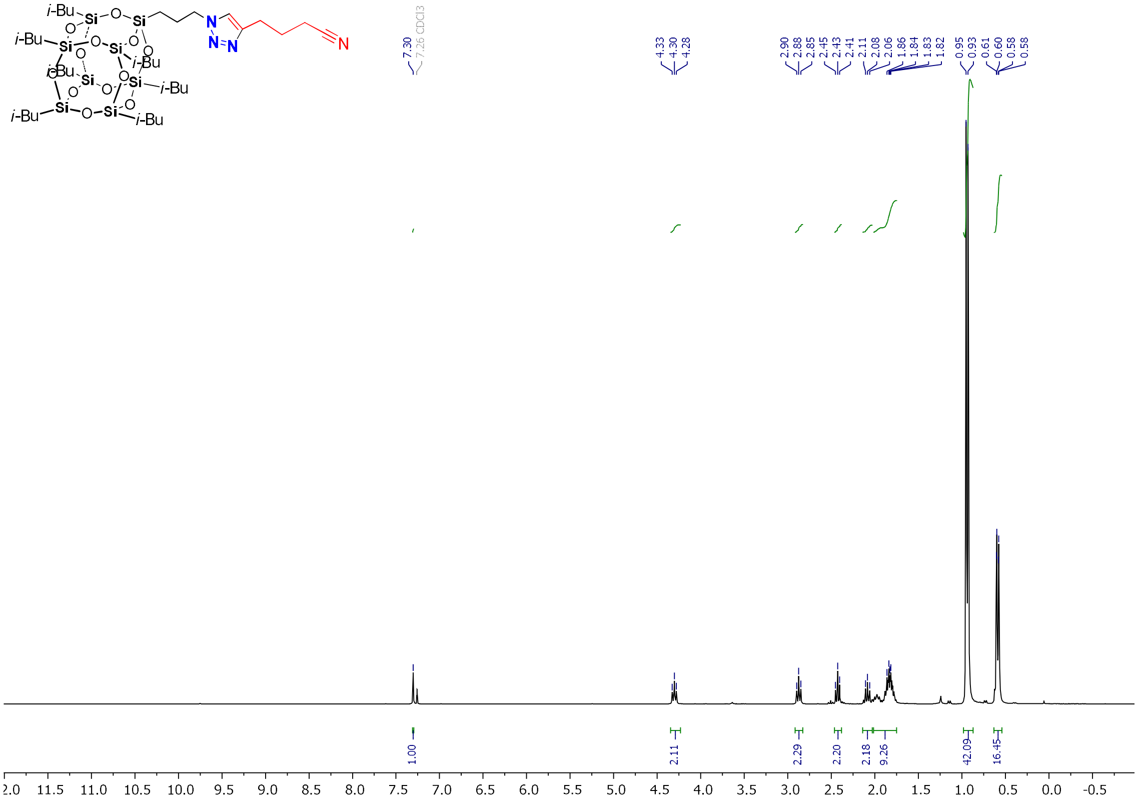

Figure S10 ^{1}H NMR spectra of iBuT$_8$-A4

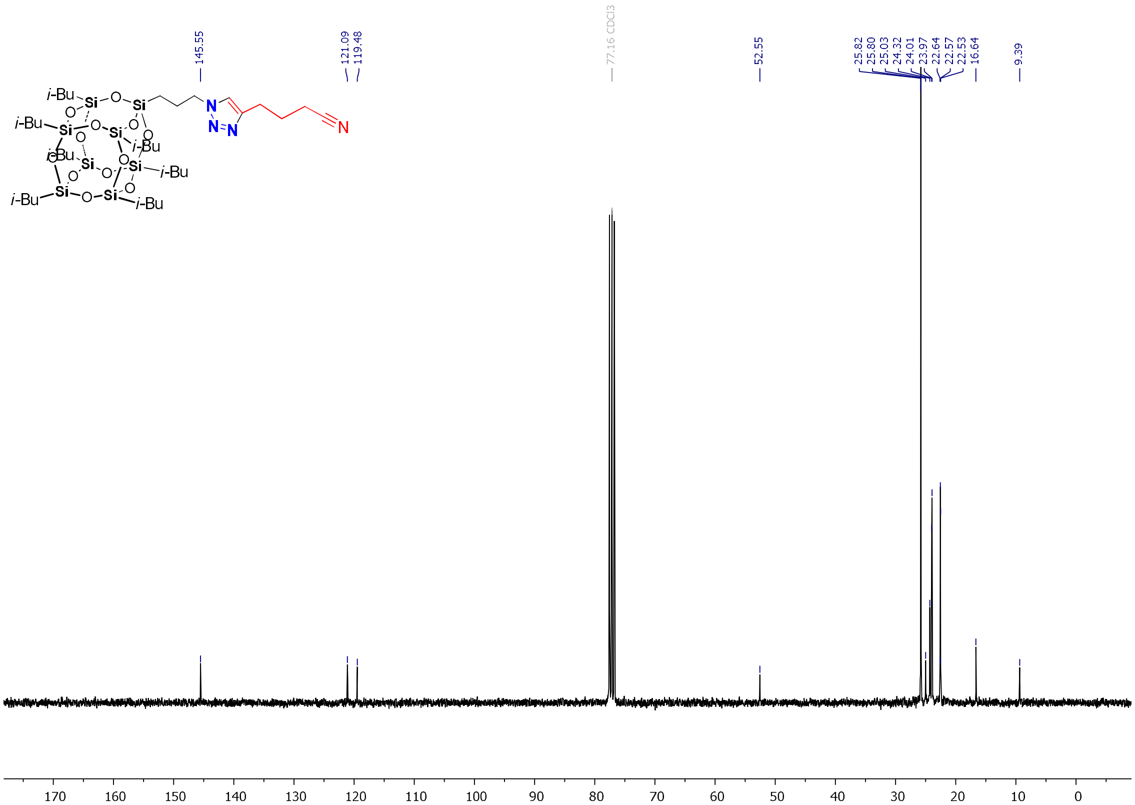

Figure S11 ^{13}C NMR spectra of iBuT$_8$-A4

iBuT$_8$-A5

White solid, 91.8%

^{1}H NMR (300 MHz, CDCL3, 25 °C) δ = 7.55-7.52 (m, 2H, NCH), 7.50-7.33 (m, 4H, PhH), 3.25 (t, 4H, $J_{H\text{-}H}$ = 7.1Hz, N-CH$_2$), 1.84 (hept, 14H, $J_{H\text{-}H}$ = 6.7 Hz, CH(CH$_3$)$_2$), 1.73-1.68 (m, 4H, CH$_2$CH$_2$CH$_2$), 0.96 (dd, 84H, $J_{H\text{-}H}$ = 6.6, 1.2Hz, CH(CH$_3$)$_2$), 0.62 (overlapped, d, 4H, $J_{H\text{-}H}$ = 2.5Hz, CH$_2$Si), 0.60 (d, 28H, $J_{H\text{-}H}$ = 2.6 Hz, CH$_2$CH(CH$_3$)$_2$); ^{13}C NMR (101 MHz, CDCl$_3$, 25 °C) δ = 131.76, 128.49, 128.39, 123.44, 89.52, 53.79, 25.85, 25,82, 24.06, 24.01, 22.65, 9.48; ^{29}Si NMR (79.5 MHz, CDCl$_3$, 25 °C) δ = -67.46, -67.54, -67.80, -67.83, -68.08; IR (cm^{-1}): 2954.61, 2868.95, 2098.35, 1264.18, 1228.74, 1096.79, 733.20, 689.83, 480.52; EA: Anal. calcd for $C_{72}H_{144}N_6O_{24}Si_{16}$ (%): C, 44.87, H, 7.53; found: C, 44.89; H, 7.63.

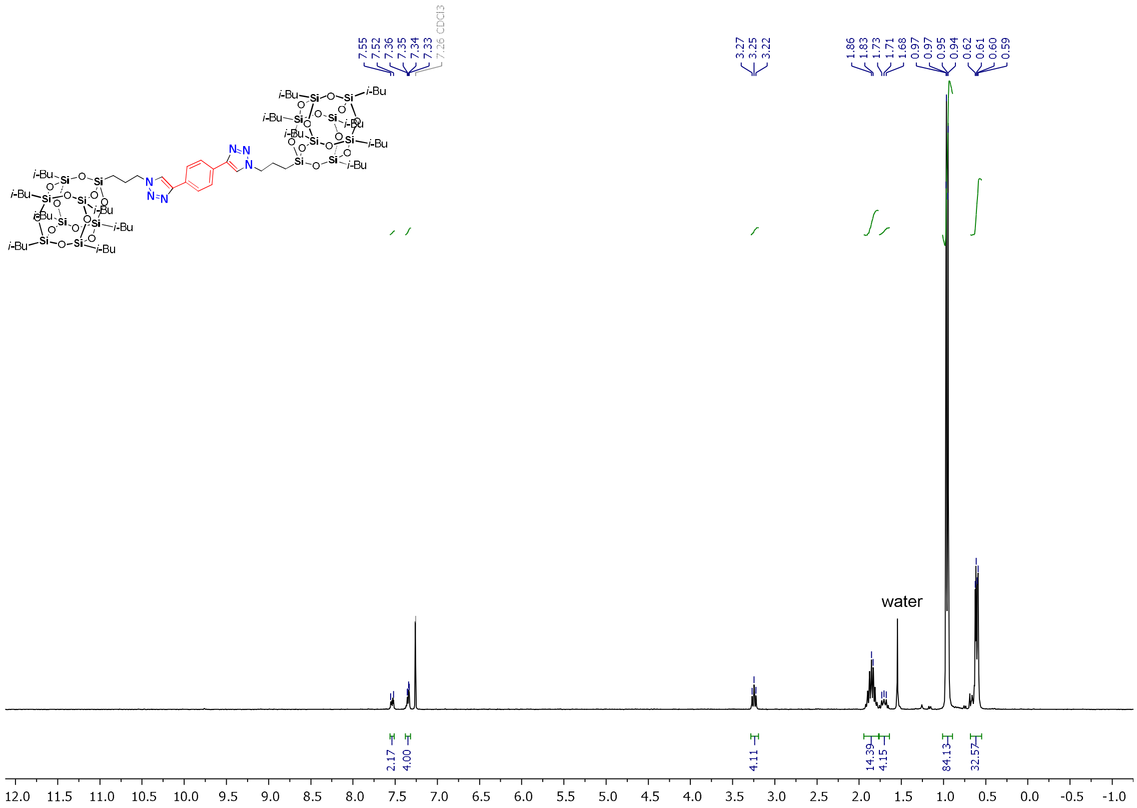

Figure S12 ^{1}H NMR spectra of iBuT$_8$-A5

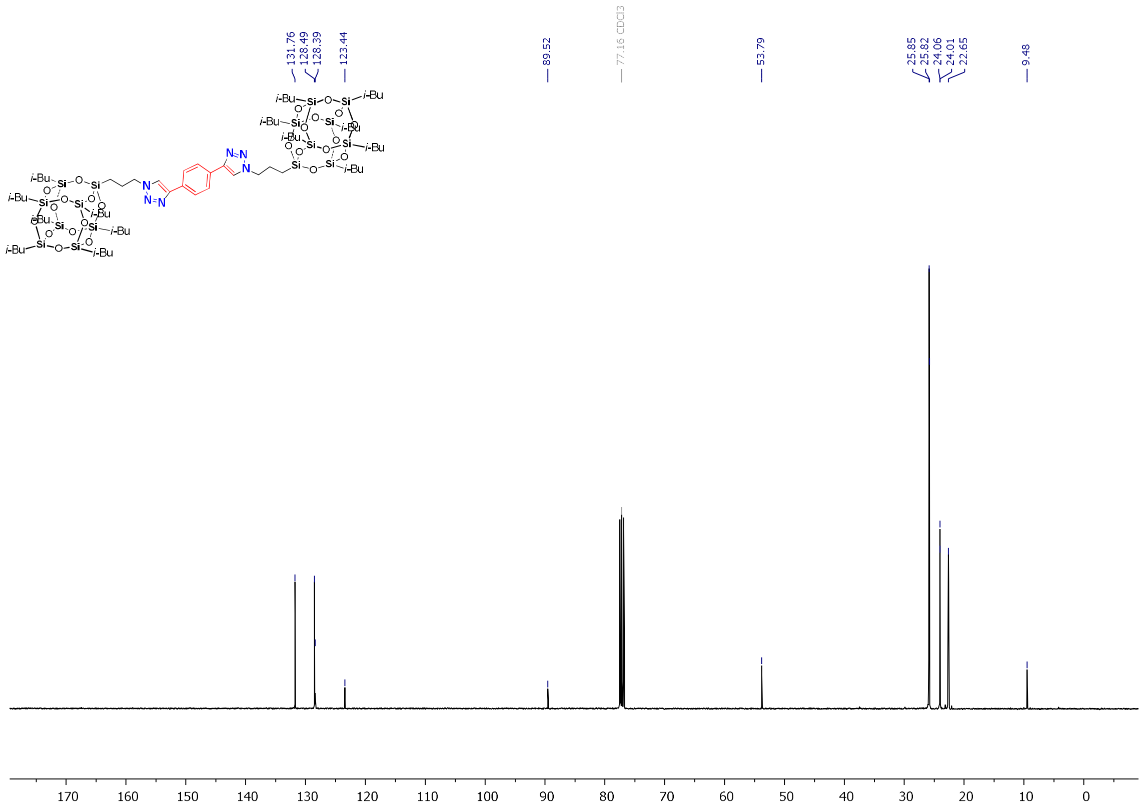

Figure S13 ^{13}C NMR spectra of iBuT$_8$-A5

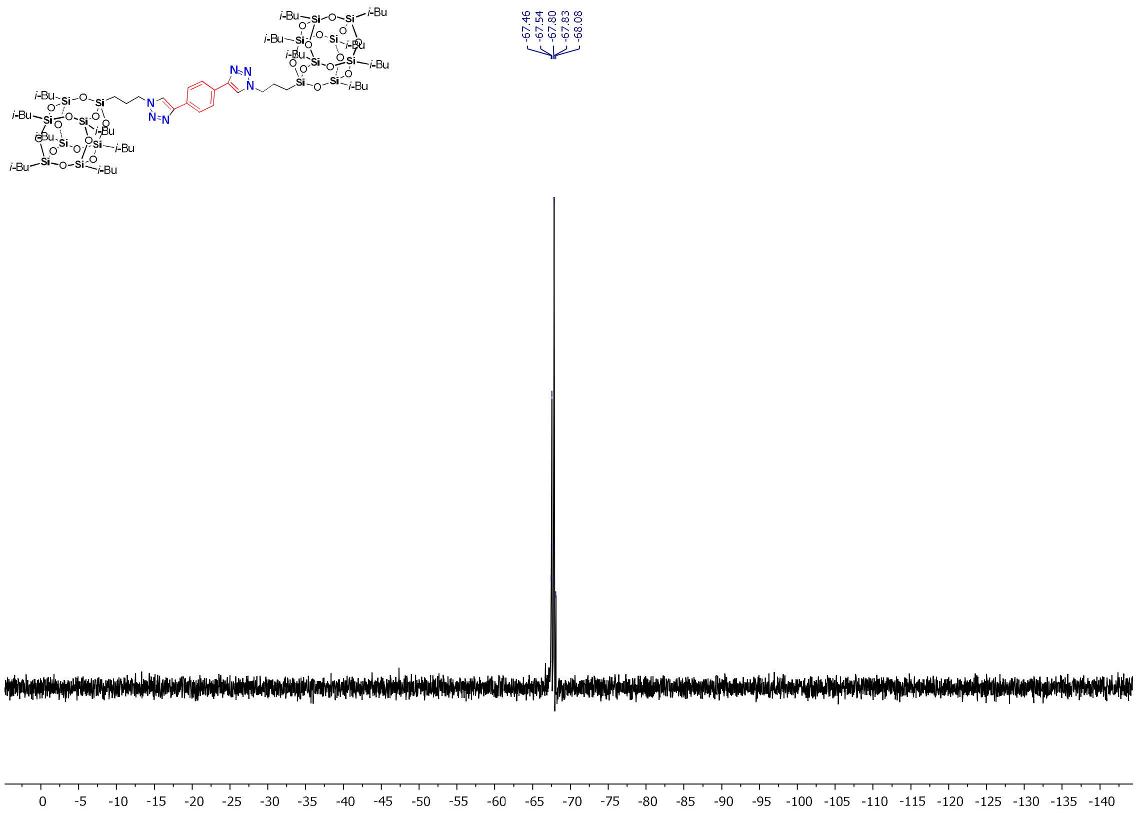

Figure S14 ^{29}Si NMR spectra of iBuT$_8$-A5

iBuT$_8$-A6

White solid,

^{1}H NMR (300 MHz, $CDCl_3$, 25 °C) δ = 7.45 (s, 1H, NCH), 7.34-7.29 (m, 5H, PhH), 4.32 (t, 2H, J_{H-H} = 7.2Hz, N-CH_2), 3.67 (s, 2H, N(CH_3)-CH_2, 3.51 (s, 2H, CH_2-N(CH_3), 2.39 (s, 3H, N(CH_3)), 2.00 (quin, 2H, $CH_2CH_2CH_2$), 1.81-1.87 (m, 7H, CH(CH_3)$_2$), 0.95 (d, 42H, J_{H-H} = 6.6 Hz, CH(CH_3)$_2$), 0.81 (overlapped, 2H, CH_2Si), 0.60 (d, 14H, J_{H-H} = 7.0Hz, CH_2CH(CH_3)$_2$); ^{13}C NMR (101 MHz, $CDCl_3$, 25 °C) δ = 145.31, 129.24, 128.45, 127.29, 122.42, 61.58, 52.99, 52.20, 42.18, 29.86, 25.84, 25.81, 24.03, 23.99, 22.61, 22.61, 22.57, 9.42; IR (cm^{-1}): 2953.23, 2925.61, 2868.89, 1463.78, 1228.18, 1090.22, 734.93, 475.90; EA: Anal. calcd for $C_{42}H_{82}N_4O_{12}Si_8$ (%): C, 47.60, H, 7.80; found: C, 47.79; H, 7.93.

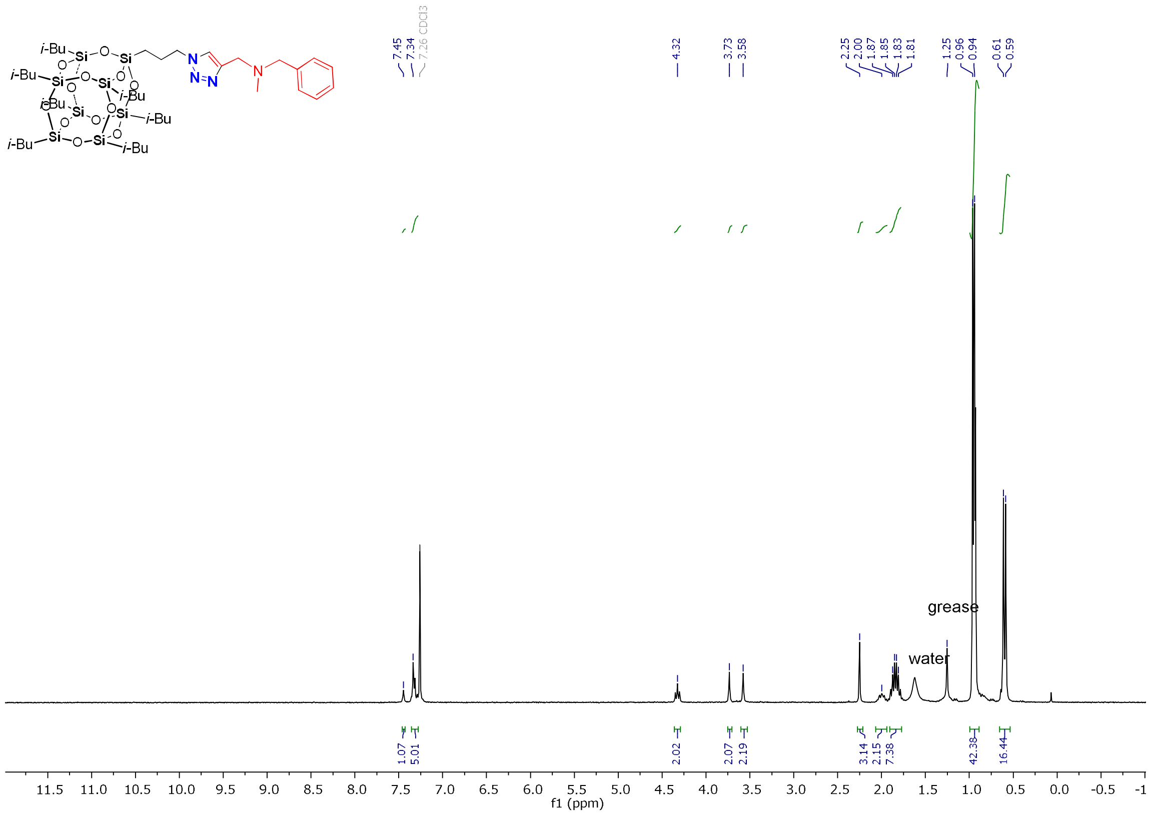

Figure S15 ^{1}H NMR spectra of iBuT$_8$-A6

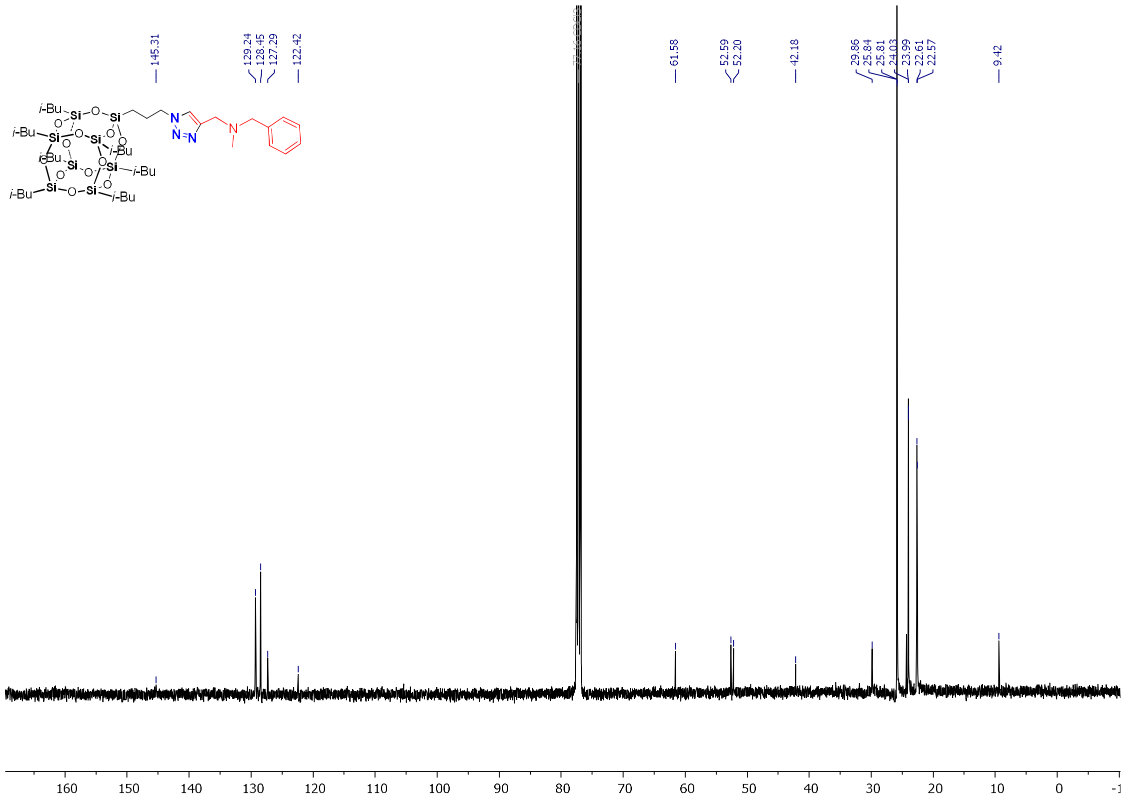

Figure S16 ^{13}C NMR spectra of iBuT$_8$-A6

iBuT$_8$-A7

Pale yellow solid, 81%

^{1}H NMR (300 MHz, $CDCl_3$, 25 °C) δ = 7.63 (s, 1H, NCH), 7.38 (dd, 1H, J_{H-H} = 3.6, 1.2Hz, tiophene-H), 7.30 (dd, 1H, J_{H-H} = 5.1, 1.2Hz, tiophene-H), 7.08 (dd, 1H, J_{H-H} = 5.1, 3.5Hz, tiophene-H), 4.37 (t, 2H, J_{H-H} = 7.2Hz, N-CH_2), 2.09-1.98 (m, 2H, $CH_2CH_2CH_2$), 1.85-1.83 (m, 7H, $CH(CH_3)_2$), 0.95 (dd, 42H, J_{H-H} = 6.0, 1.1Hz, $CH(CH_3)_2$), 0.60 (dd, 14H, J_{H-H} = 7.1, 2.6Hz, $CH_2CH(CH_3)_2$). ^{13}C NMR (101 MHz, $CDCl_3$, 25 °C) δ = 142.95, 133.24, 127.73, 125.07, 124.14, 118.94, 52.69, 25.84, 25.82, 24.34, 24.05, 22.68, 22.61, 9.36; ^{29}Si NMR (79.5 MHz, $CDCl_3$, 25 °C) δ = -67.53, -67.84, -68.60; IR (cm^{-1}): 3118.39, 2951.56, 2925.73, 2867.66, 2625.33, 2625.33, 1463.32, 1365.63, 1331.36, 1288.82, 1168.24, 1088.81, 1051.23, 837.70. 740.20, 477.52; EA: Anal. calcd for $C_{37}H_{73}N_3O_{12}SSi_8$ (%): C, 44.05, H, 7.29; found: C, 44.09; H, 7.33.

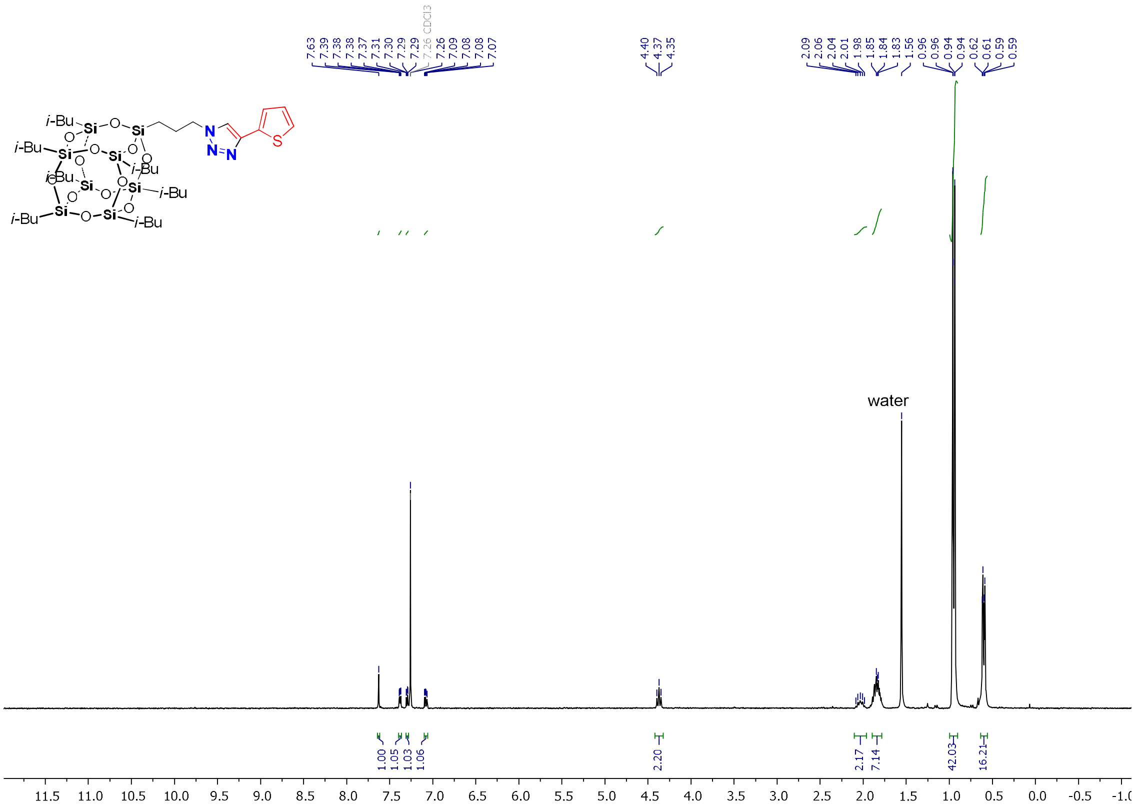

Figure S17 ^{1}H NMR spectra of iBuT$_8$-A7

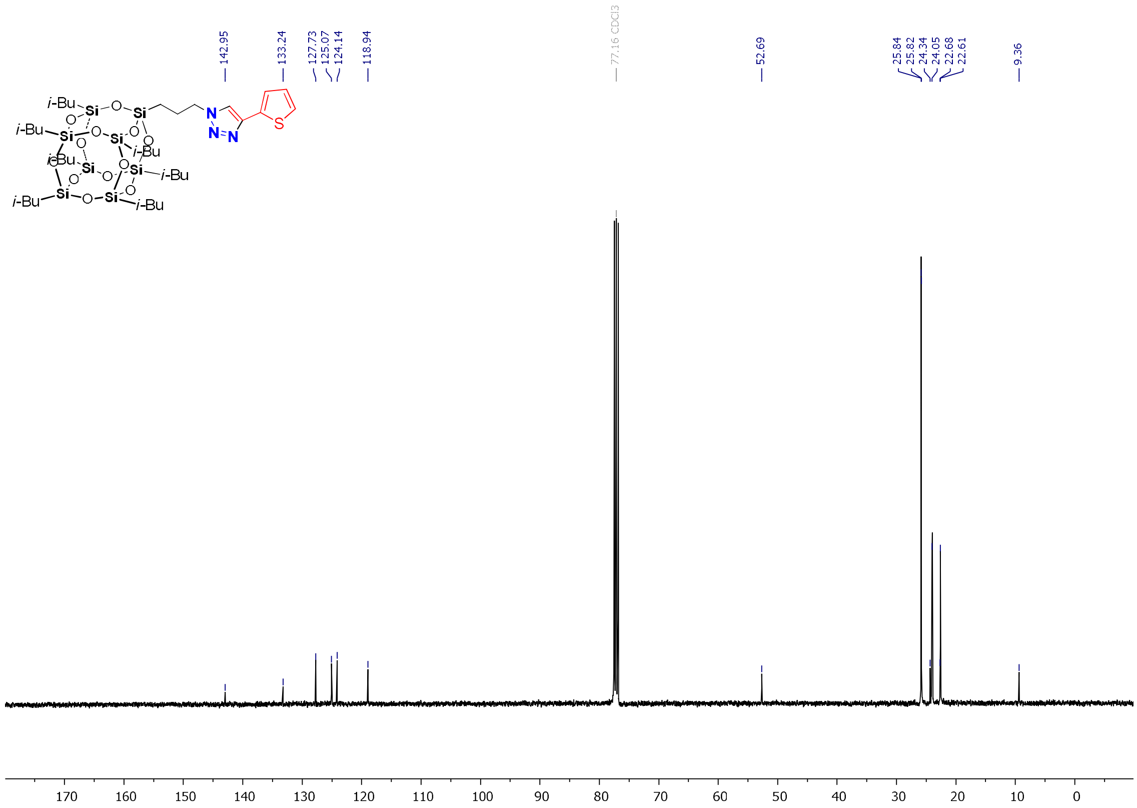

Figure S18 ^{13}C NMR spectra of iBuT$_8$-A7

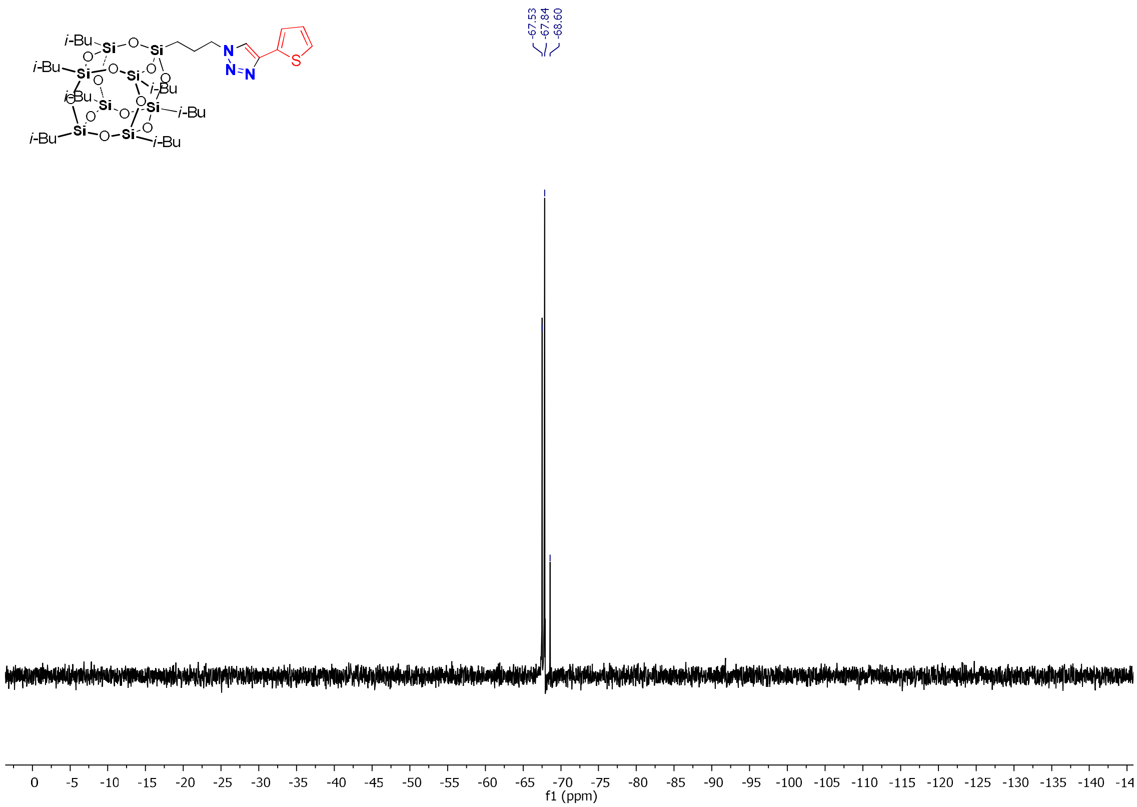

Figure S19 ^{29}Si NMR spectra of iBuT$_8$-A7

iBuT$_8$-A8

White solid, 84 %

^{1}H NMR (300 MHz, $CDCl_3$, 25 °C) δ = 7.58 (s, 1H, NCH), 4.34 (t, $J_{H\text{-}H}$ = 7.3 Hz, 2H, N-CH_2), 2.00 (quin, 2H, $CH_2CH_2CH_2$), 1.83-1.85 (m, 14H, $CH(CH_3)_2$), 0.96-0.94 (m, 82H, $CH(CH_3)_2$), 0.61 (overlapped, s, 4H, CH_2Si), 0.59 (s, 28H, $CH_2CH(CH_3)_2$), 0.45 (s, 6H, $(CH_3)_2Si$); ^{13}C NMR (101 MHz, $CDCl_3$, 25 °C) δ = 145.66, 129.41, 52.15, 25.85, 24.56, 24.03, 24.00, 23.95, 22.63, 22.59, 9.61, 0.82, -0.01; ^{29}Si NMR (79.5 MHz, $CDCl_3$, 25 °C) δ = -3.57, -66.74, -66.99, -67.55, -67.84, -67.87, -68.57, -109.66; IR (cm^{-1}): 2953.16, 2906.19, 2868.38, 1464.54, 1264.22, 1229.19, 1094.67, 737.09, 480.11; EA: Anal. calcd for $C_{63}H_{139}N_3O_{25}Si_{17}$ (%): C, 41.66, H, 7.71; found: C, 41.79; H, 7.87.

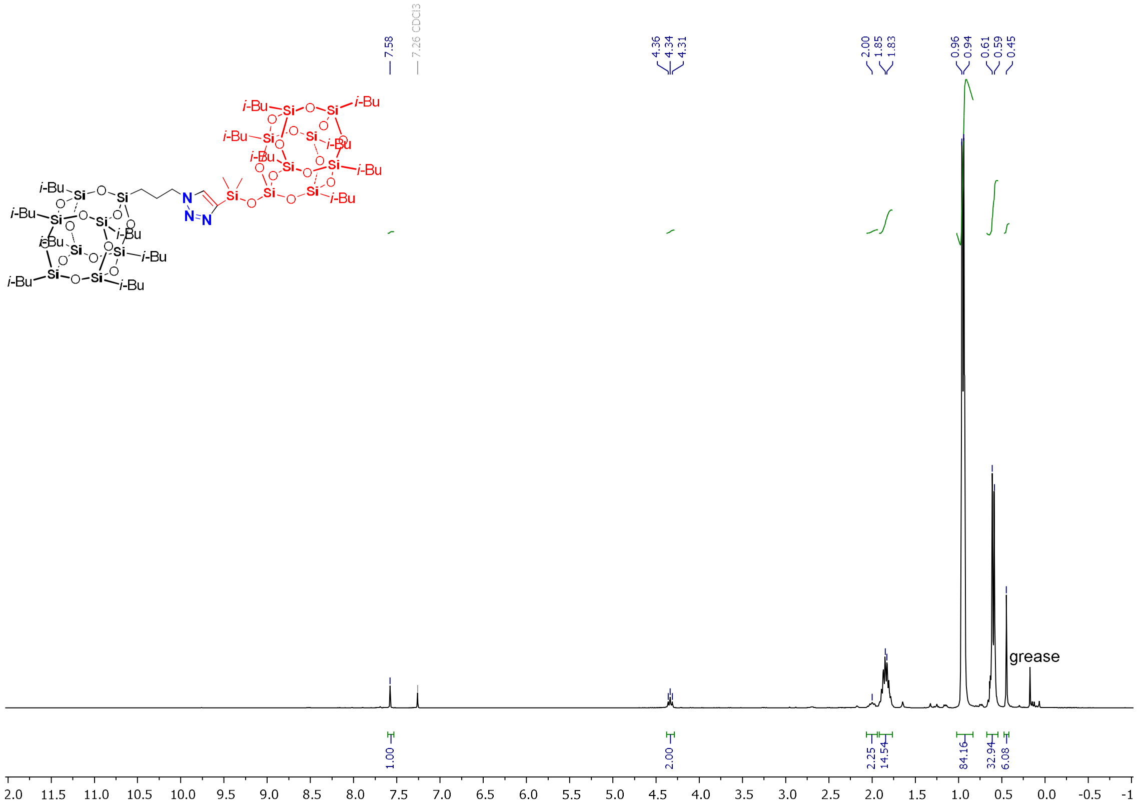

Figure S20 ^{1}H NMR spectra of iBuT$_8$-A8

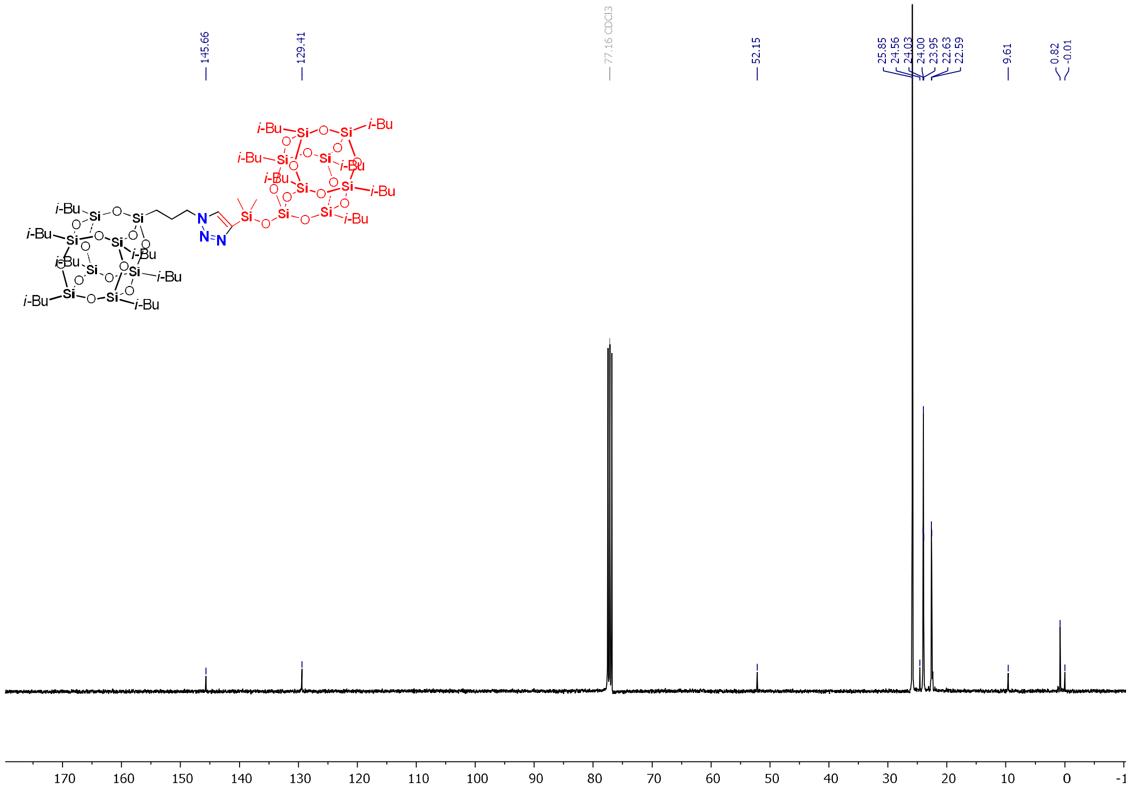

Figure S21 ^{13}C NMR spectra of iBuT$_8$-A8

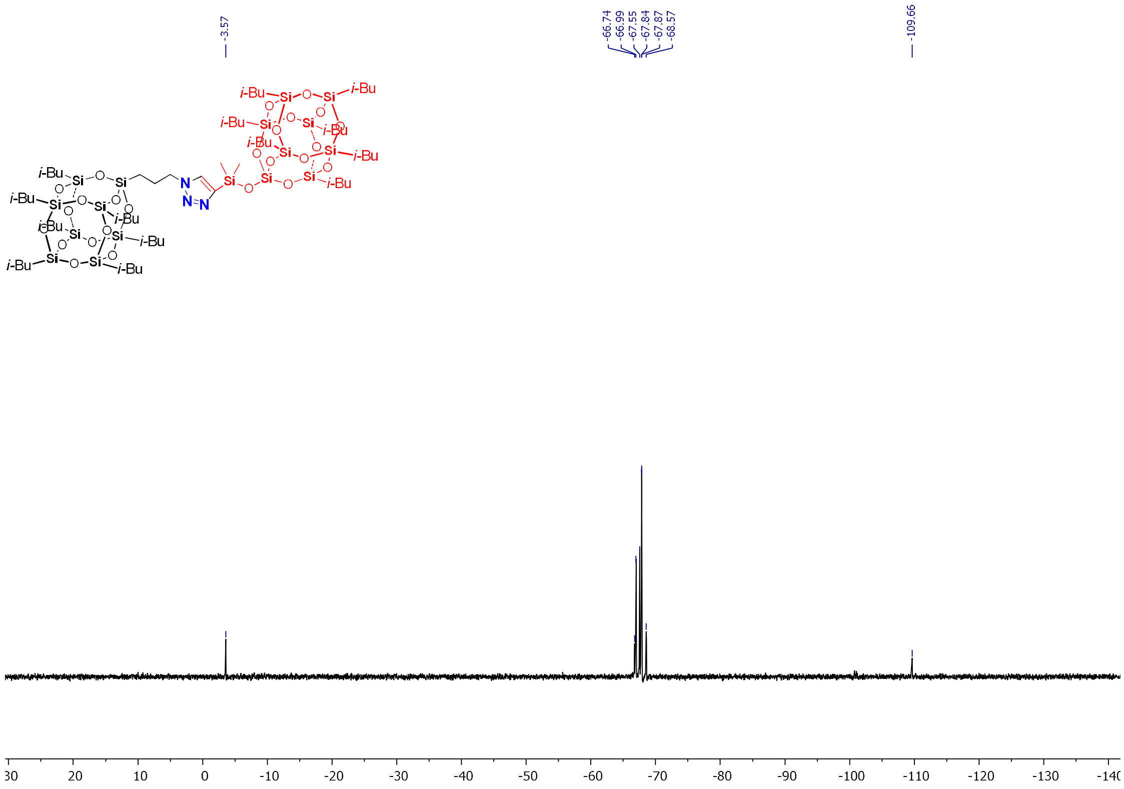

Figure S22 ^{29}Si NMR spectra of iBuT$_8$-A8

iBuT$_8$-A9

White solid, 70%

^{1}H NMR (300 MHz, $CDCl_3$, 25 °C) δ = 7.61-7.59 (m, 2H, PhH), 7.42-7.36 (m, 4H, PhH(3), NCH(1)), 4.34 (t, 2H, $J_{H\text{-}H}$ = 7.4 Hz, N-CH_2), 2.04-1.96 (m, 2H, $CH_2CH_2CH_2$), 1.91-1.80 (m, 7H, $CH(CH_3)_2$), 0.97-0.93 (m, 42H, $CH(CH_3)_2$), 0.63 (overlapped, 2H, CH_2Si), 0.61-0.58 (m, 14H, $CH_2CH(CH_3)_2$), 0.56 (overlapped, 6H, $CH(CH_3)_2$); ^{13}C NMR (101 MHz, $CDCl_3$, 25 °C) δ = 144.99, 137.40, 134.09, 133.13, 129.54, 128.06, 53.78, 25.84, 25.81, 24.01, 22.61, 22.56, 9.47, -2.21; ^{29}Si NMR (79.5 MHz, $CDCl_3$, 25 °C) δ = -14.75, -67.56, -67.85, -68.12, -68.51; IR (cm^{-1}): 2954.07, 2925.88, 2869.02, 2098.85, 1464.59, 1264.39, 1228.48, 1092.26, 735.14, 476.68; EA: Anal. calcd for $C_{41}H_{81}N_3O_{12}Si_9$ (%): C, 46.42, H, 7.70; found: C, 46.59; H, 7.83.

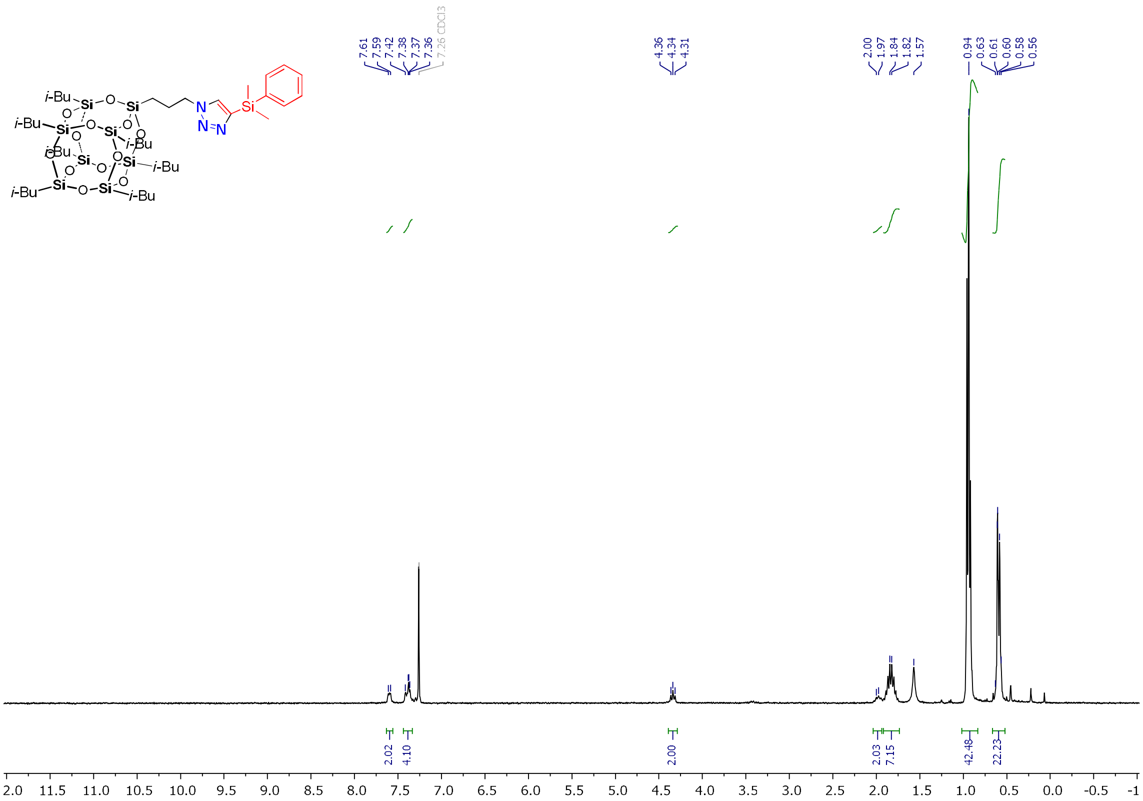

Figure S23 ^{1}H NMR spectra of iBuT$_8$-A9

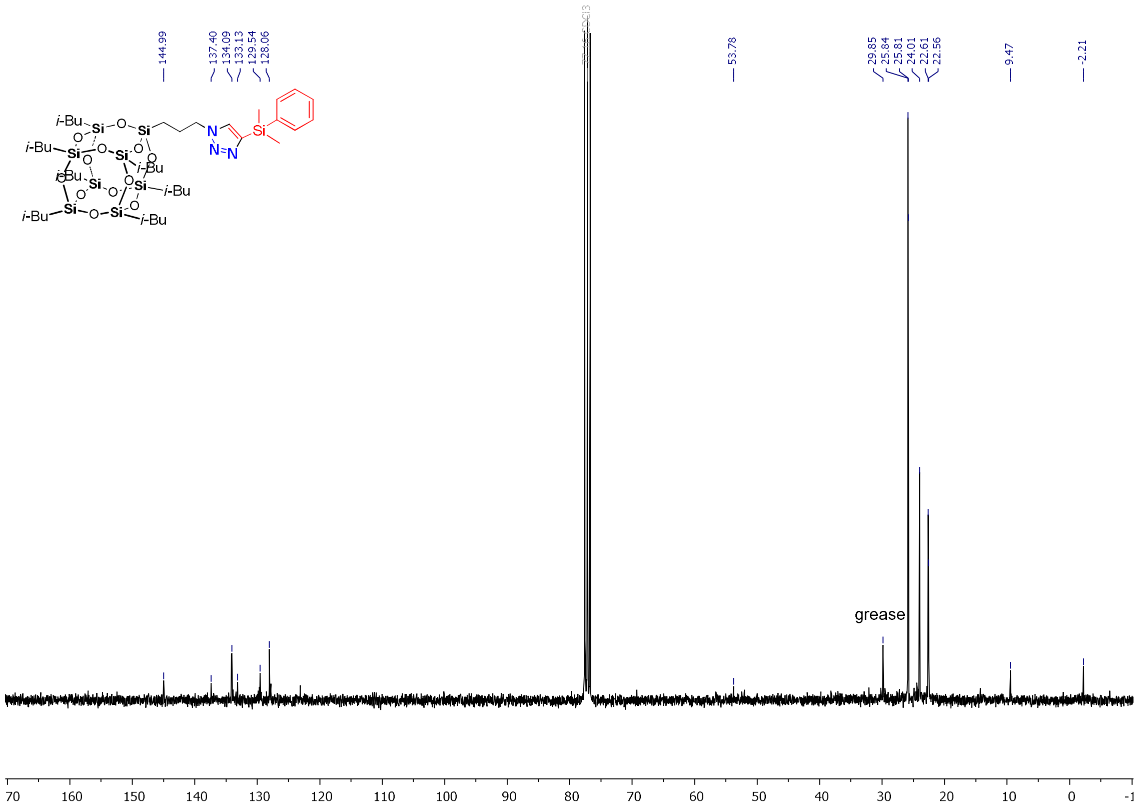

Figure S24 ^{13}C NMR spectra of iBuT$_8$-A9

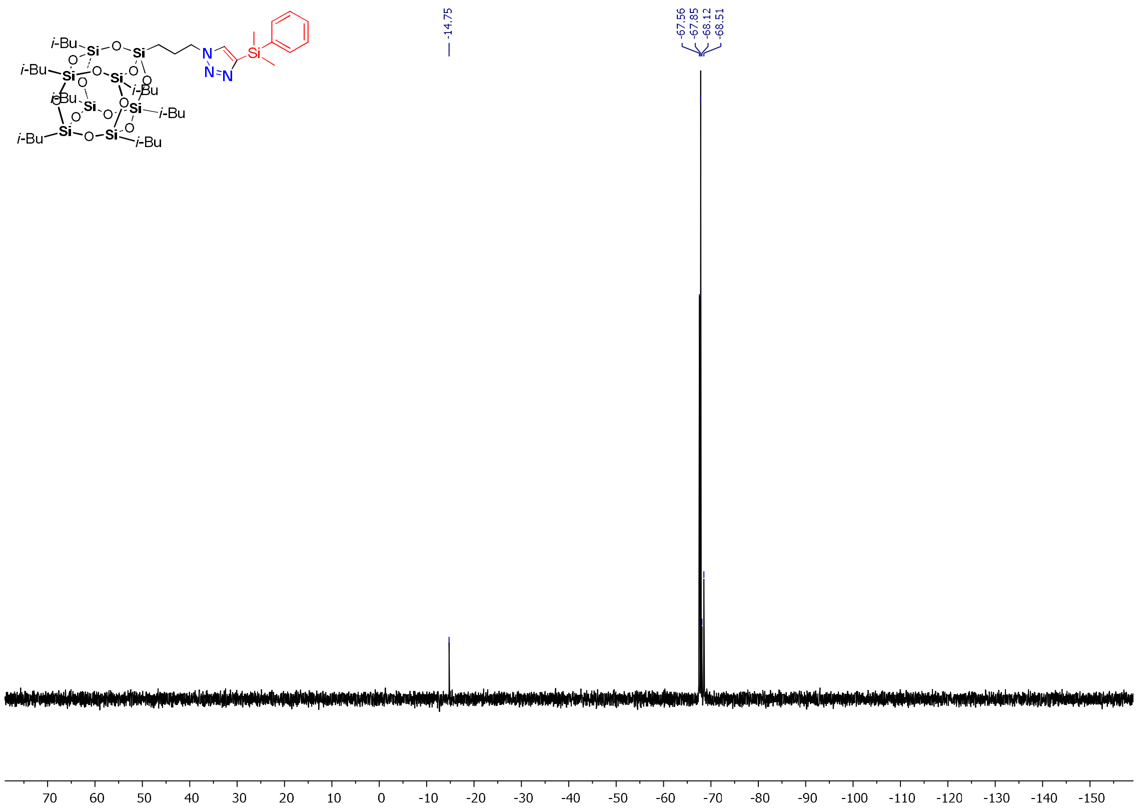

Figure S25 ^{29}Si NMR spectra of iBuT$_8$-A9

iBuT$_8$-A10

Yellow solid, 85%

^{1}H NMR (300 MHz, $CDCl_3$, 25 °C) δ = 7.42 (s, 1H, NCH), 4.3 (t, 2H, J_{H-H} = 7.4 Hz, N-CH_2), 2.04-1.96 (m, 2H, $CH_2CH_2CH_2$), 1.89-1.79 (m, 7H, $CH(CH_3)_2$)), 1.17-0.82 (m, 57H, $Ge(CH_2CH_3)_3$ (15), $CH(CH_3)_2$ (42)), 0.64 (overlapped, 2H, CH_2Si), 0.63-0.60 (m, 14H, $CH_2CH(CH_3)_2$); ^{13}C NMR (101 MHz, $CDCl_3$, 25 °C) δ = 128.30, 52.01, 25.83, 25.80, 24.46, 24.04, 23.99, 22.61, 22.57, 9.44, 9.07, 4.70; ^{29}Si NMR (79.5 MHz, $CDCl_3$, 25 °C) δ = -67.56, -67.85, -67.91, -68.43; IR (cm^{-1}): 2951.73, 2905.47, 2870.66, 1463.27, 1356.64, 1228.50, 1088.06, 837.21, 739.46, 474.09; EA: Anal. calcd for $C_{39}H_{85}GeN_3O_{12}Si_8$ (%): C, 43.16, H, 7.89; found: C, 43.29; H, 7.93.

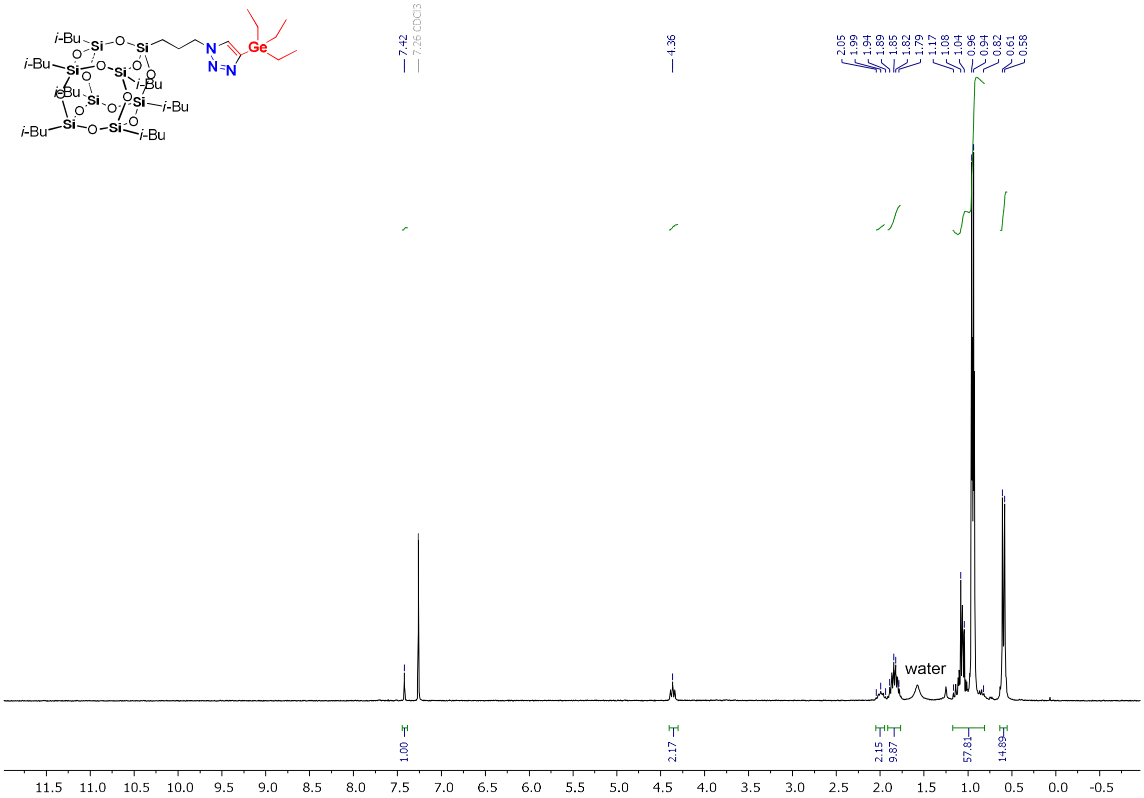

Figure S26 ^{1}H NMR spectra of iBuT$_8$-A10

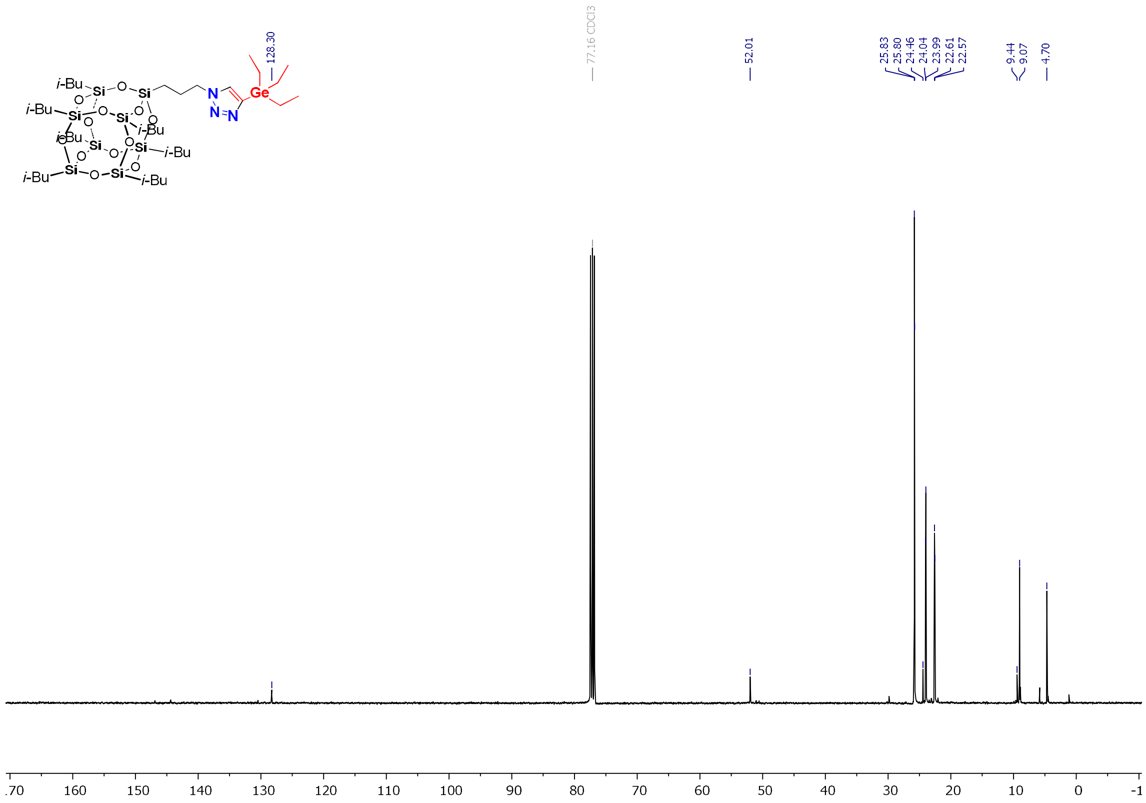

Figure S27 ^{13}C NMR spectra of iBuT$_8$-A10

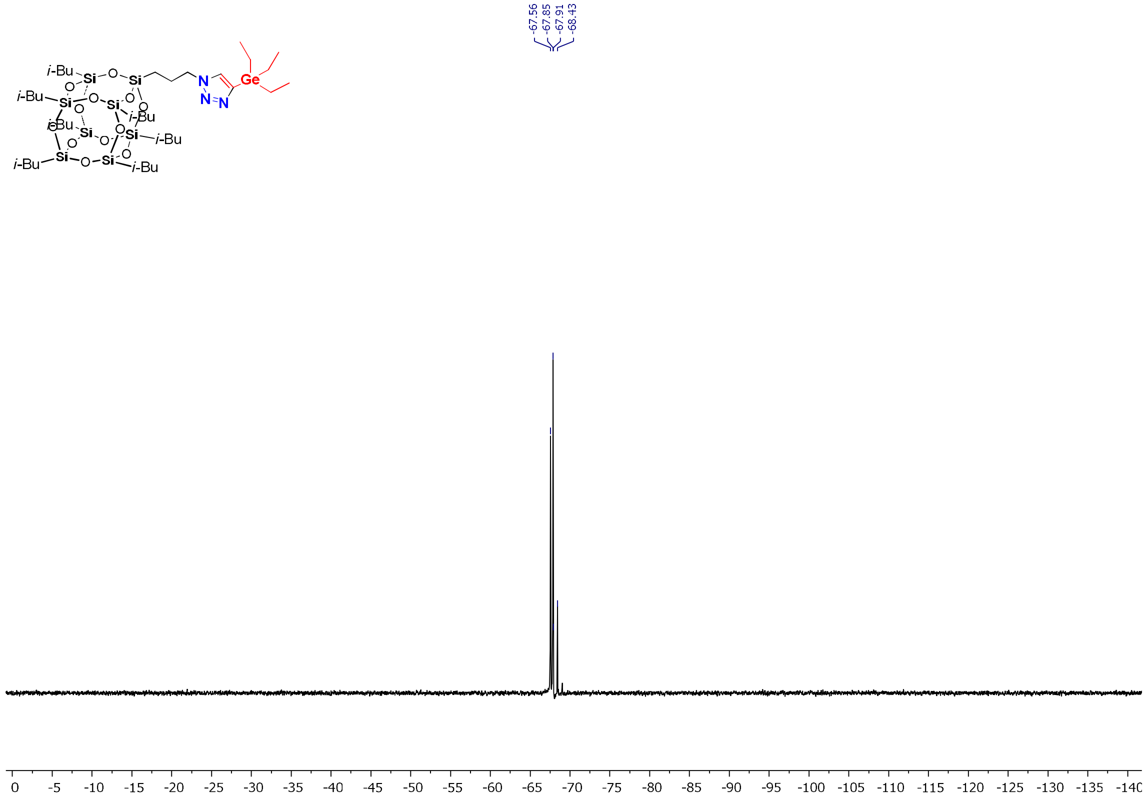

Figure S28 ^{29}Si NMR spectra of iBuT$_8$-A10

DDSQ-2A1

White solid, 85%

^{1}H NMR (300 MHz, $CDCl_3$, 25 °C) δ = 8.57-8.56 (m, 2H, PyH), 8.11 (d, 2H, J_{H-H} = 7.9 Hz, PyH), 7.84 (s, 2H, NCH), 7.75 (td, 2H, J_{H-H} = 7.1,1.8Hz, PyH), 7.50- 7.18 (m, 40H, Ph), 4.21 (t, 4H, J_{H-H} = 7.1Hz, N-CH_2), 1.95 (quin, 4H, $CH_2CH_2CH_2$), 0.75-0.70 (m, 4H, CH_2Si), 0.30 (s, 6H, $Si(CH_3)$); ^{13}C NMR (101 MHz, $CDCl_3$, 25 °C) δ = 150.52, 149.46, 148.37, 136.91, 134.09, 133.93, 131.64, 130.82, 130.69, 128.06, 127.99, 127.94, 127.85, 122.82, 120.28, 52.82, 24.11, 13.77, -0.78; ^{29}Si NMR (79.5 MHz, $CDCl_3$, 25°C) δ = -20.52, -80.22, -81.31;IR (cm^{-1}): 3071.85, 3050.34, 3025.77, 1429.81, 1082.52, 730.61, 696.69, 487.88; EA: Anal. calcd for $C_{70}H_{68}N_8O_{14}Si_{10}$ (%): C, 55.09, H, 4.49; found: C, 55.19; H, 4.53.

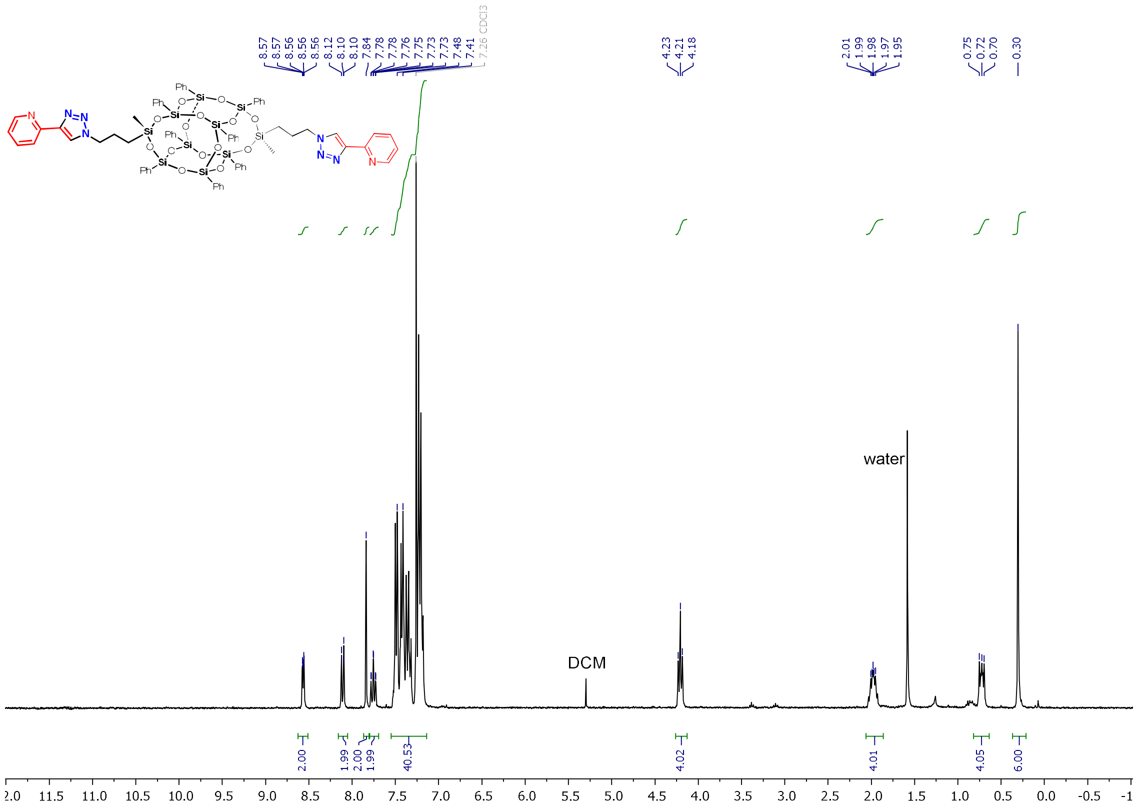

Figure S29 ^{1}H NMR spectra of DDSQ-2A1

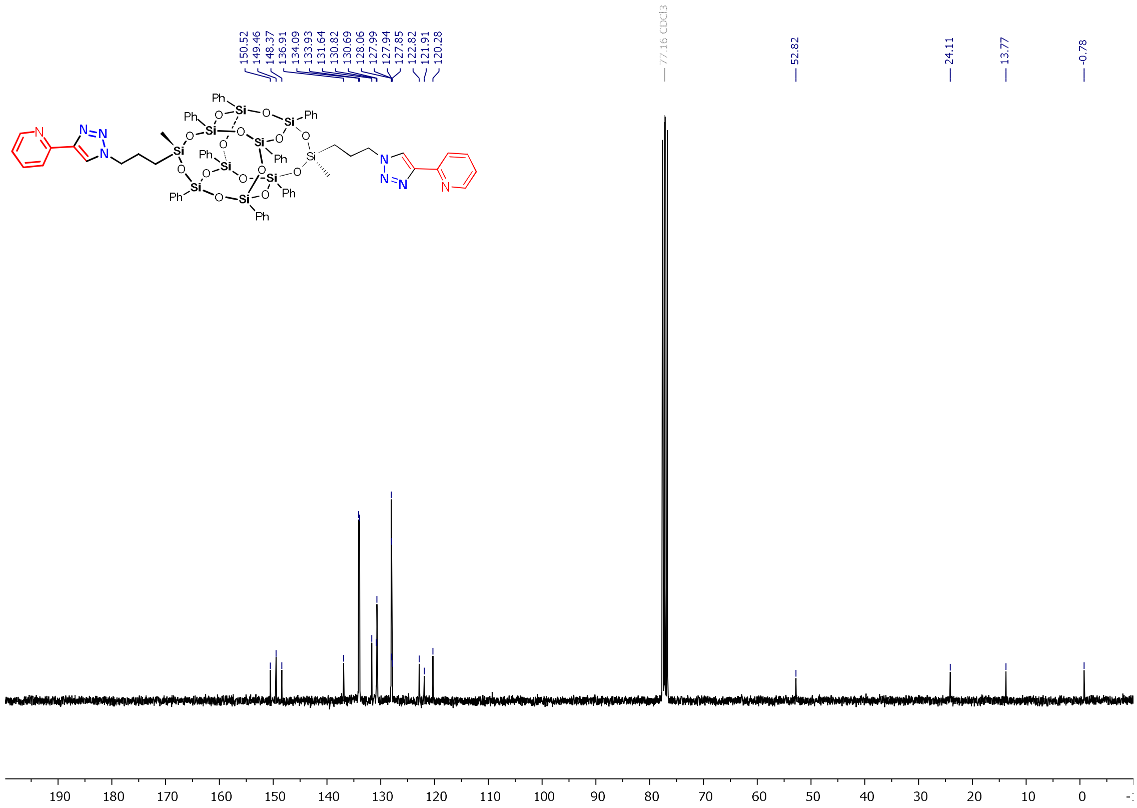

Figure S30 ^{13}C NMR spectra of DDSQ-2A1

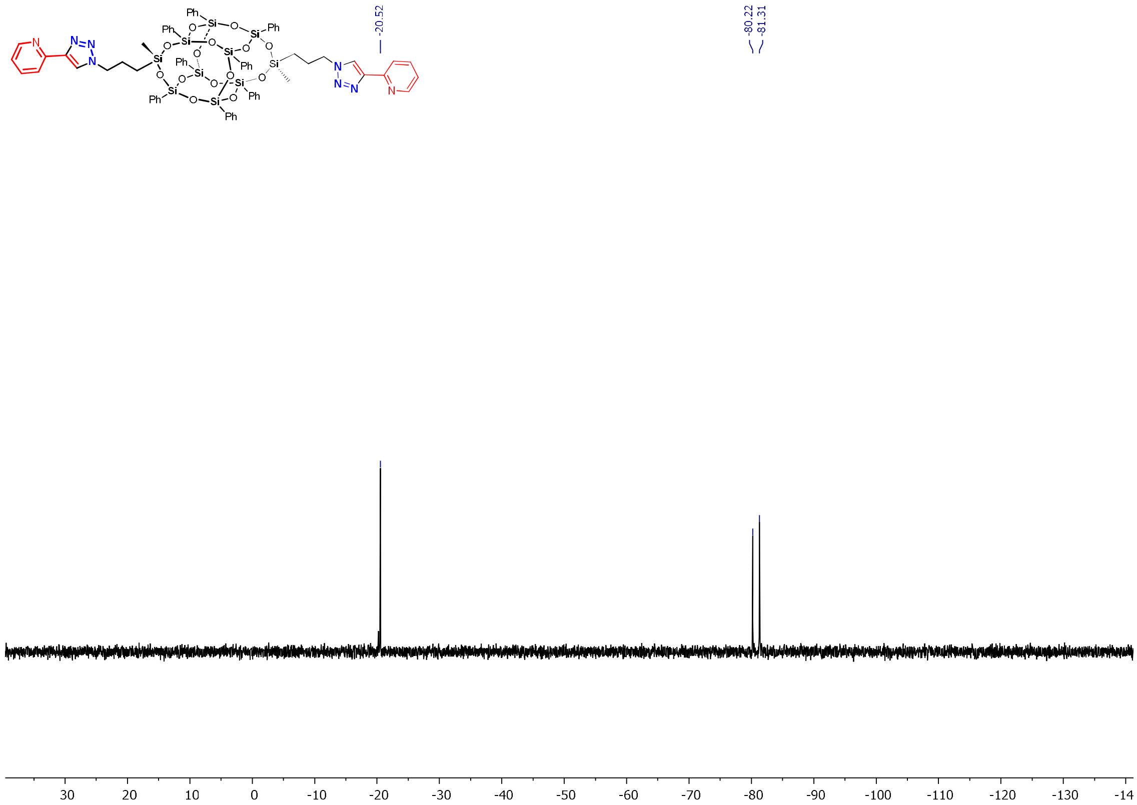

Figure S31 ^{29}Si NMR spectra of DDSQ-2A1

DDSQ-2A2

White solid, 60%

^{1}H NMR (300 MHz, $CDCl_3$, 25 °C) δ = 7.49-7.08 (m, 52H, Ph), 6.64 (s, 2H, NCH), 4.10 (t, 4H, J_{H-H} = 7.1 Hz, N-CH_2), 2.83 (s, 8H, CH_2CH_2Ph), 1.93-1.88 (m, 4H, $CH_2CH_2CH_2$), 0.68-0.62 (m, 4H, CH_2Si), 0.30 (s, 6H, $Si(CH_3)$); ^{13}C NMR (101 MHz, $CDCl_3$, 25 °C) δ = 141.34, 134.02, 133.96, 133.91, 131.61, 130.86, 130.79, 130.75, 128.51, 128.45, 128.07, 127.89, 127.96, 127.85, 126.14, 52.32, 35.66, 29.82, 27.53, 24.10, 13.58, -0.73; IR (cm^{-1}): 3071.87, 3026.15, 2923.83, 1429.73, 1076.50, 727.11, 695.38, 481.92.

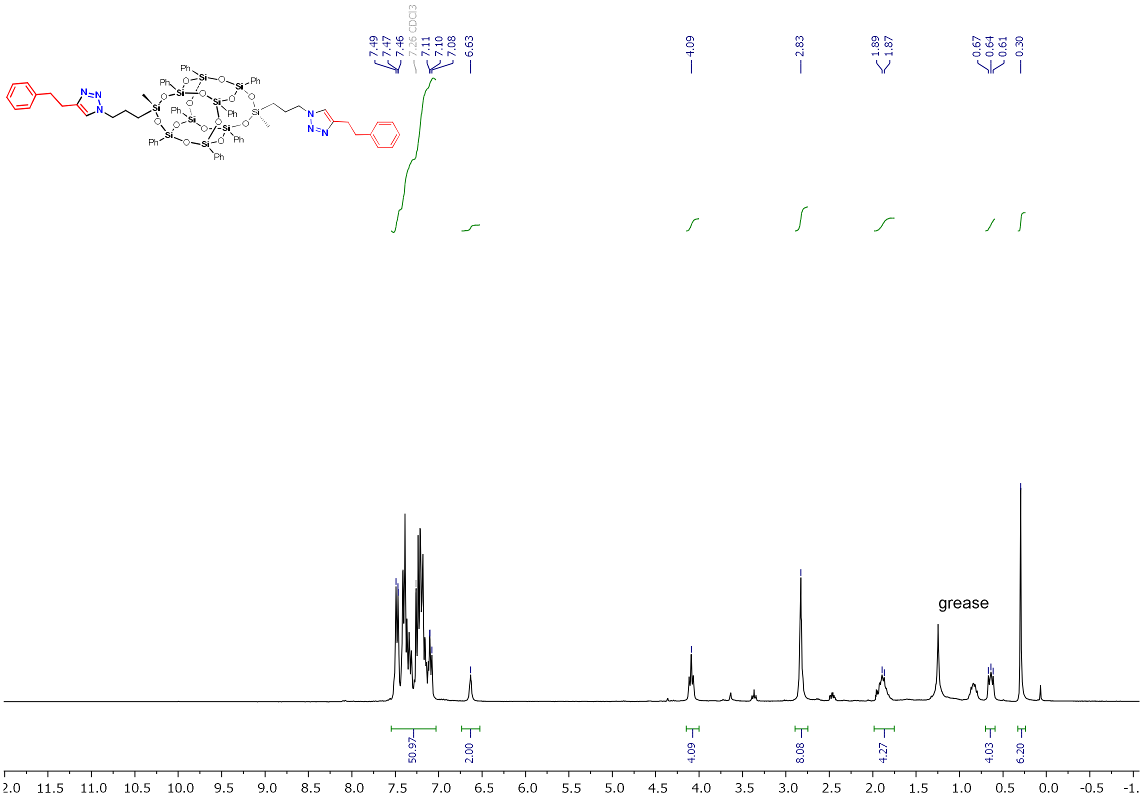

Figure S32 ^{1}H NMR spectra of DDSQ-2A2

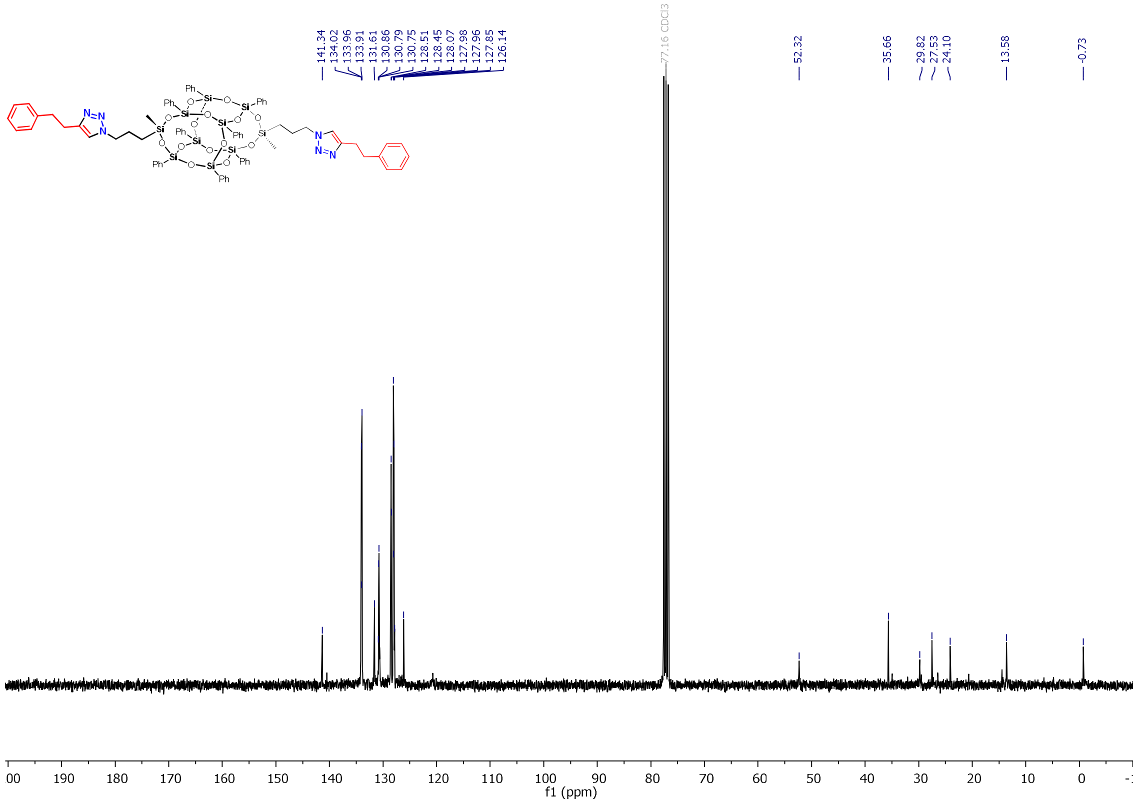

Figure S33 ^{13}C NMR spectra of DDSQ-2A2

DDSQ-2A3

White solid, 82%

^{1}H NMR (300 MHz, $CDCl_3$, 25 °C) δ = 7.60-7.17 (m, 52H, Ph (40), PhH (10), NCH (2)), 4.19 (t, 4H, J_{H-H} = 7.2 Hz, N-CH_2), 2.04-1.94 (m, 4H, $CH_2CH_2CH_2$), 0.74 (t, 4H, CH_2Si), 0.74-0.68 (m, 4H, CH_2Si), 0.30 (s, 6H, $Si(CH_3)$); ^{13}C NMR (101 MHz, $CDCl_3$, 25 °C) δ = 147.70, 134.04, 133.93, 131.59, 130.79, 128.80, 128.11, 128.01, 125.81, 119.33, 52.51, 24.20, 19.03, 13.63, -0.63; IR (cm^{-1}): 3051.67, 2926.43, 1430.03, 1264.20, 1084.28, 730.69, 696.38, 486.53.

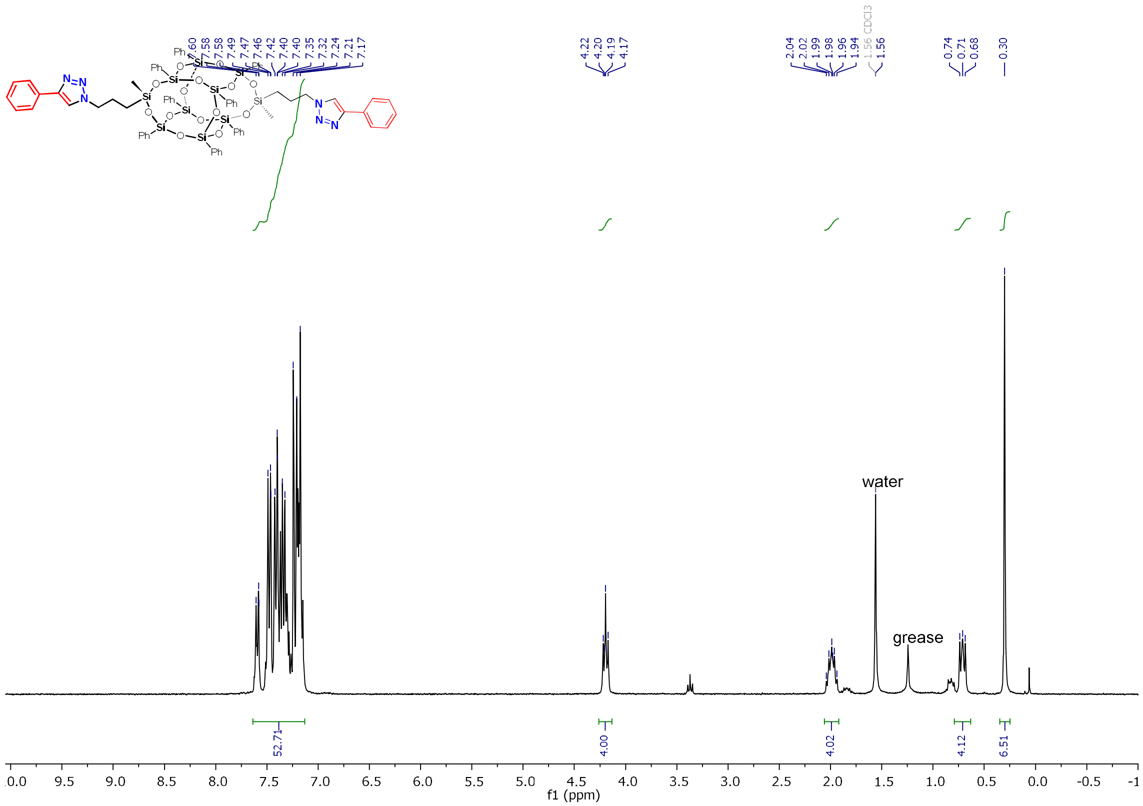

Figure S34 ^{1}H NMR spectra of DDSQ-2A3

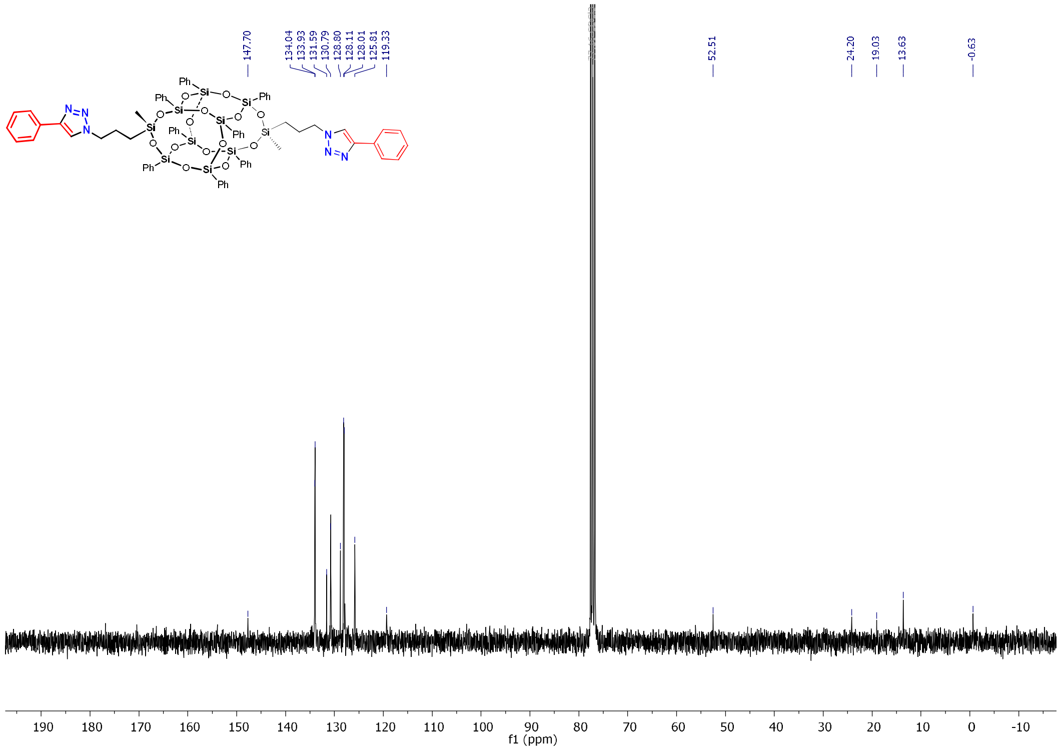

Figure S35 ^{13}C NMR spectra of DDSQ-2A3

DDSQ-2A4

White solid, 78%

^{1}H NMR (300 MHz, $CDCl_3$, 25 °C) δ = 7.50-7.22 (m, 40H, Ph), 6.75 (s, 2H, NCH), 4.15 (t, 4H, J_{H-H} = 7.3 Hz, N-CH_2), 2.61 (t, 4H, J_{H-H} = 7.4 Hz, $CH_2(CH_2)_2CN$), 2.28 (t, 4H, J_{H-H} = 7.1 Hz, CH_2CH_2CN), 2.00-1.86 (m, 4H, $CH_2CH_2CH_2$), 0.71-0.66 (m, 4H, CH_2Si), 0.32 (s, 6H, $Si(CH_3)$); ^{13}C NMR (101 MHz, $CDCl_3$, 25 °C) δ = 145.43, 134.02, 131.53, 130.82, 130.77, 128.11, 128.01, 120.92, 119.50, 52.34, 24.94, 24.22, 24.12, 16.59, 13.55, -0.67; IR (cm^{-1}): 3072.18, 2930.43, 1429.93, 1089.08, 729.48, 698.44, 491.

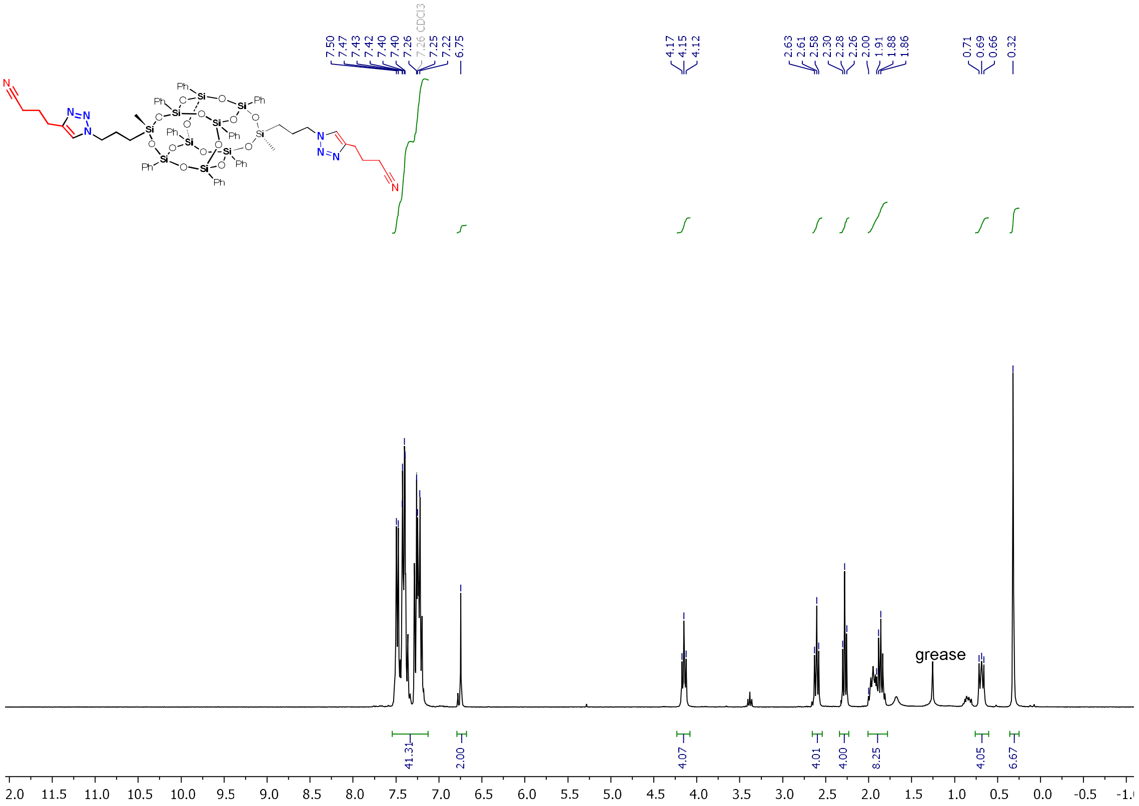

Figure S36 ^{1}H NMR spectra of DDSQ-2A4

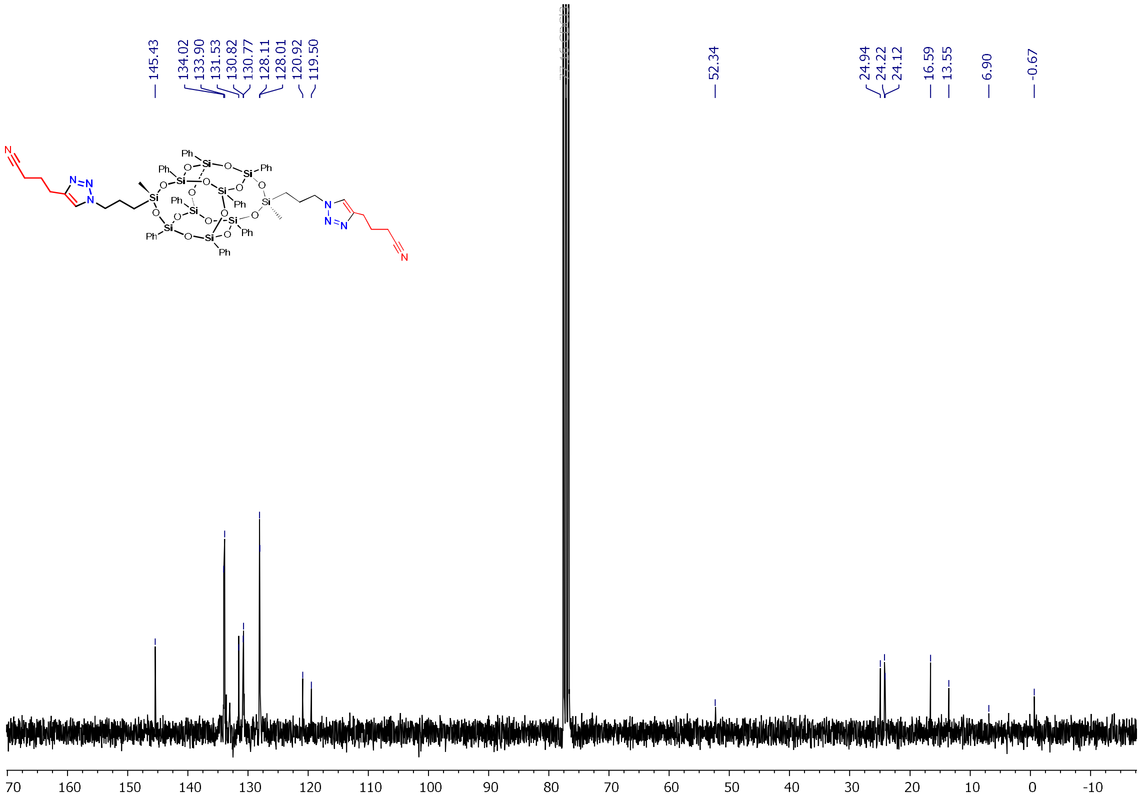

Figure S37 ^{13}C NMR spectra of DDSQ-2A4

DDSQ-2A11

White solid, 84%

^{1}H NMR (300 MHz, $CDCl_3$, 25 °C) δ = 7.51-7.19 (m, 40H, Ph), 6.73 (s, 2H, NCH), 4.13 (t, 4H, $J_{H\text{-}H}$ = 7.3Hz, N-CH_2), 2.55-2.50 (m, 4H, $CH_2(CH_2)_3CH_3$), 1.99-1.88 (m, 4H, $CH_2CH_2CH_2$), 1.52 (quin, 4H, $CH_2(CH_2)_3CH_3$), 1.29-1.26 (m, $CH_2CH_2CH_3$), 1.19 (sext, 4H, CH_2CH_3), 1.17 (quin, 4H, $CH_2CH_2CH_3$), 0.77 (t, 6H, CH_2CH_3), 0.61 (t, 4H, CH_2Si), 0.23 (s, 6H, $Si(CH_3)$); ^{13}C NMR (101 MHz, CDCl3, 25 °C) δ = 148.36, 134.03, 133.92, 131.62, 130.90, 130.73, 128.06, 127.96, 120.28, 52.29, 31.62, 29.28, 25.70, 24.15, 22.54, 14.18, 13.62; IR (cm^{-1}): 3072.28, 3050.43, 2926.54, 2856.12, 1429.88, 1085.16, 728.60, 697.24, 488.54.

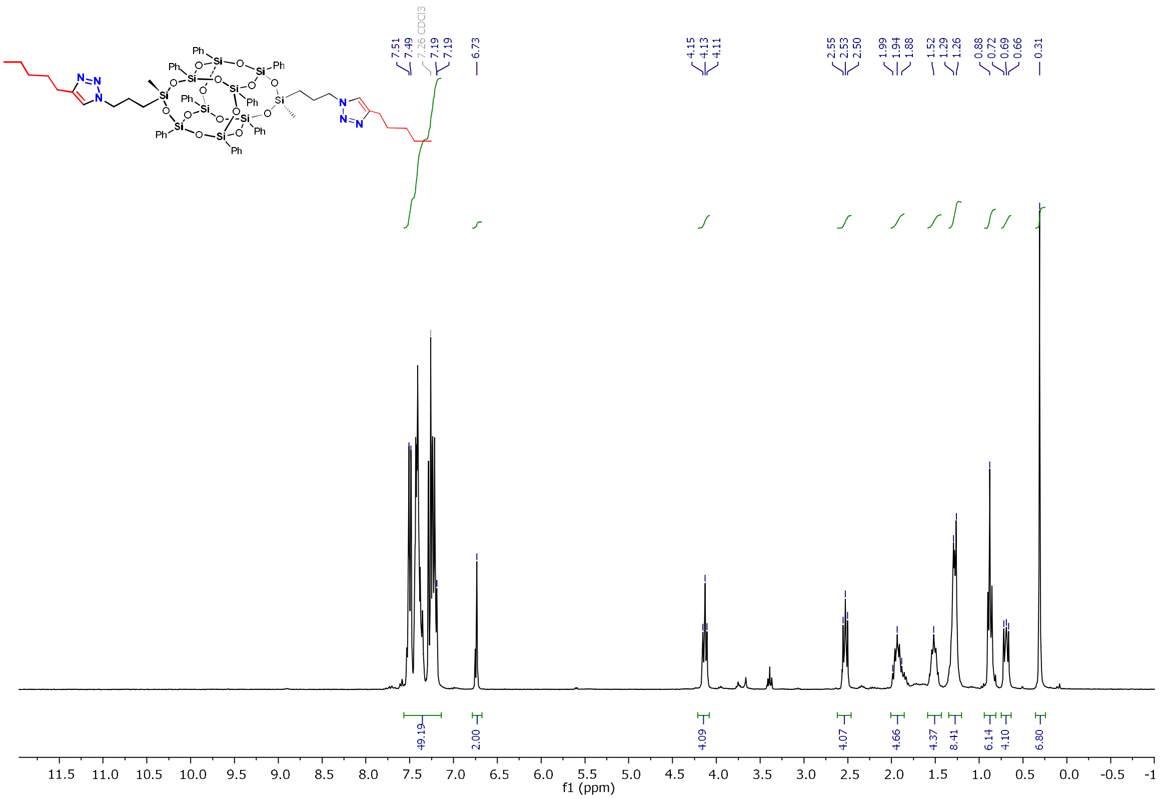

Figure S38 ^{1}H NMR spectra of DDSQ-2A11

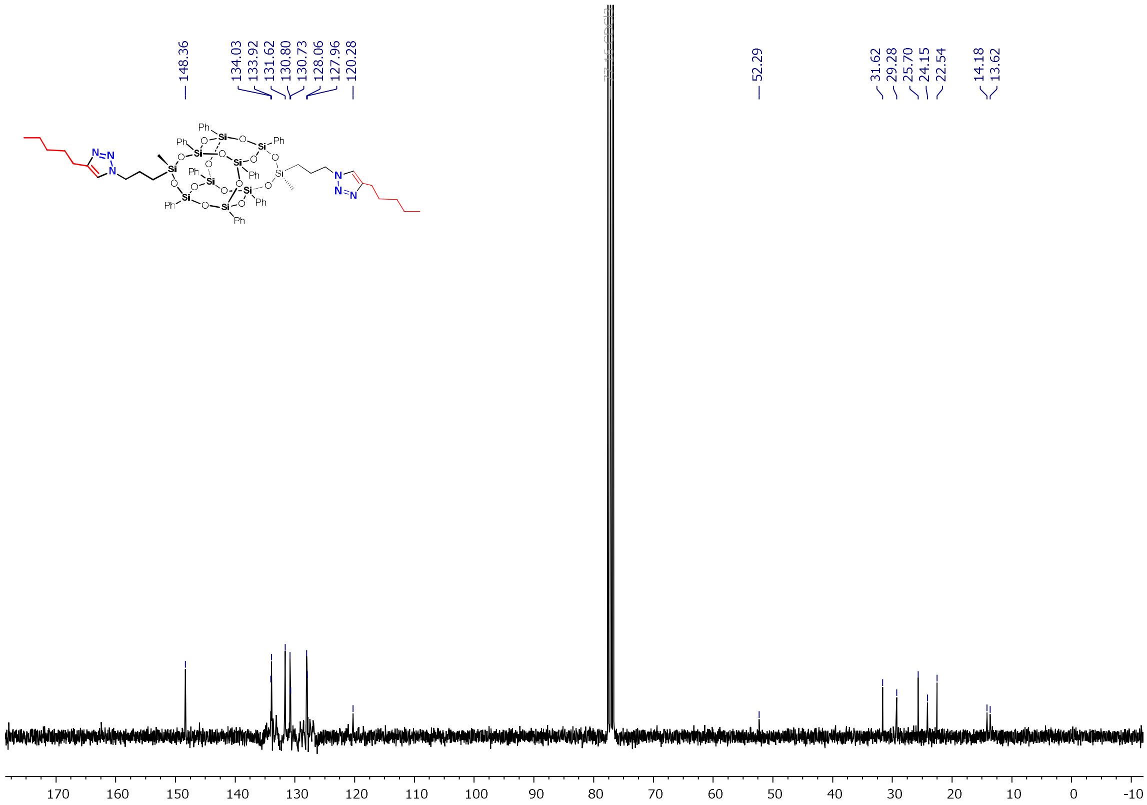

Figure S39 ^{13}C NMR spectra of DDSQ-2A11

Spectra of obtained complexes:

(iBuT$_8$-A1)$_2$-Rh(N^N)

Orange solid, 55%

^{1}H NMR (300 MHz, $CDCl_3$, 25 °C) δ = 10.47 (s, 2H, NCH), 8.93 (d, 2H, J = 8.0 Hz, PyH), 8.02 (s, 2H, PyH), 7.65 (s, 2H, PyH), 7.38 (s, 2H, PyH), 4.53 (t, 4H, N-CH_2), 2.11-2.08 (m, 4H, $CH_2CH_2CH_2$), 1.88-1.77 (m, 14H, $CH(CH_3)_2$), 0.94-0.92 (m, 84H, $CH(CH_3)_2$), 0.66-0.57 (m, 32H, CH_2Si(4), $Si(CH_3)$(28)); ^{13}C NMR (101 MHz, $CDCl_3$, 25 °C) δ = 150.33, 148.49, 147.40, 141.63, 128.79, 128.29, 125.31, 54.52, 31.33, 28.14, 25.86, 25.83, 25.79, 24.05, 24.01, 23.98, 23.96, 22.64, 22.59, 22.56, 22.53, 9.13; ^{29}Si NMR (79.5 MHz, $CDCl_3$, 25 °C) δ = -67.54, -67.89, -68.73; ESI-MS calcd for $C_{76}H_{149}Cl_2N_8O_{24}Rh_2Si_{16}^+$ [M + H]$^+$: 2281.4475, found 2281.6539; IR (cm^{-1}): 2952.93, 2924.64, 2869.19, 1464.05, 1331.71, 1104.40, 742.48, 483.52.

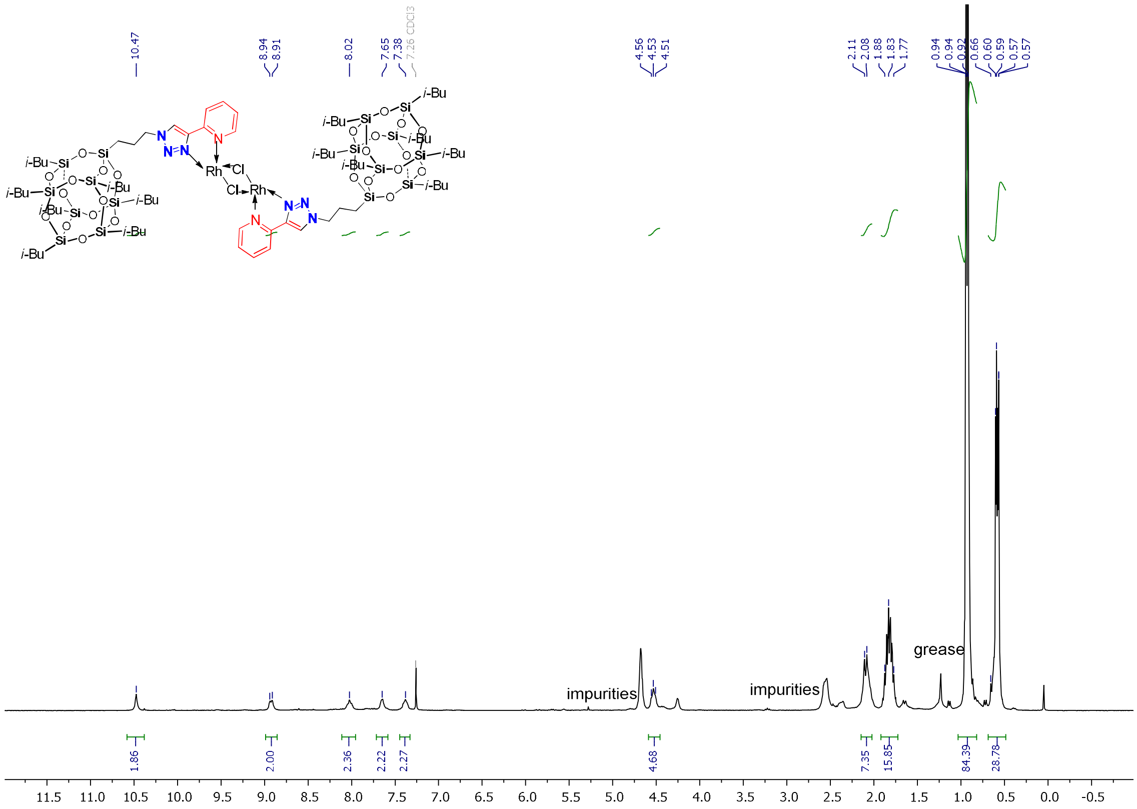

Figure S40 ^{1}H NMR spectra of (iBuT$_8$-A1)$_2$-Rh(N^N)

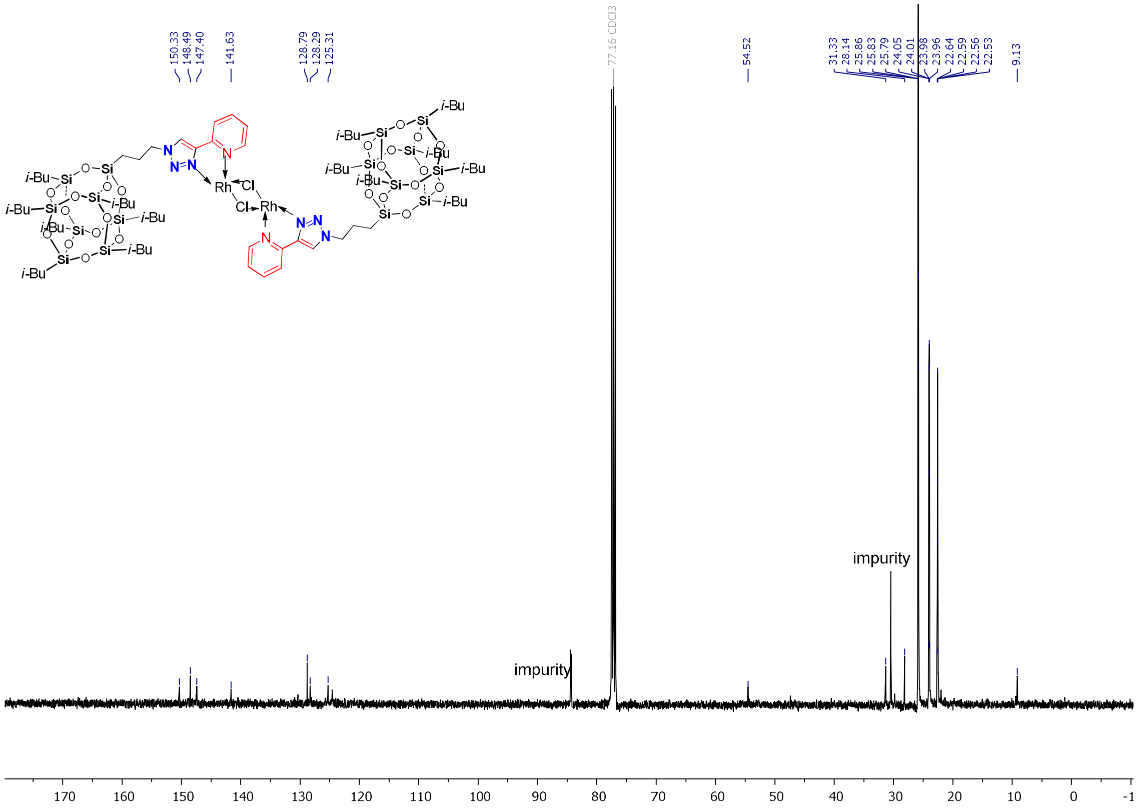

Figure S41 ^{13}C NMR spectra of (iBuT$_8$-A1)$_2$-Rh(N^N)

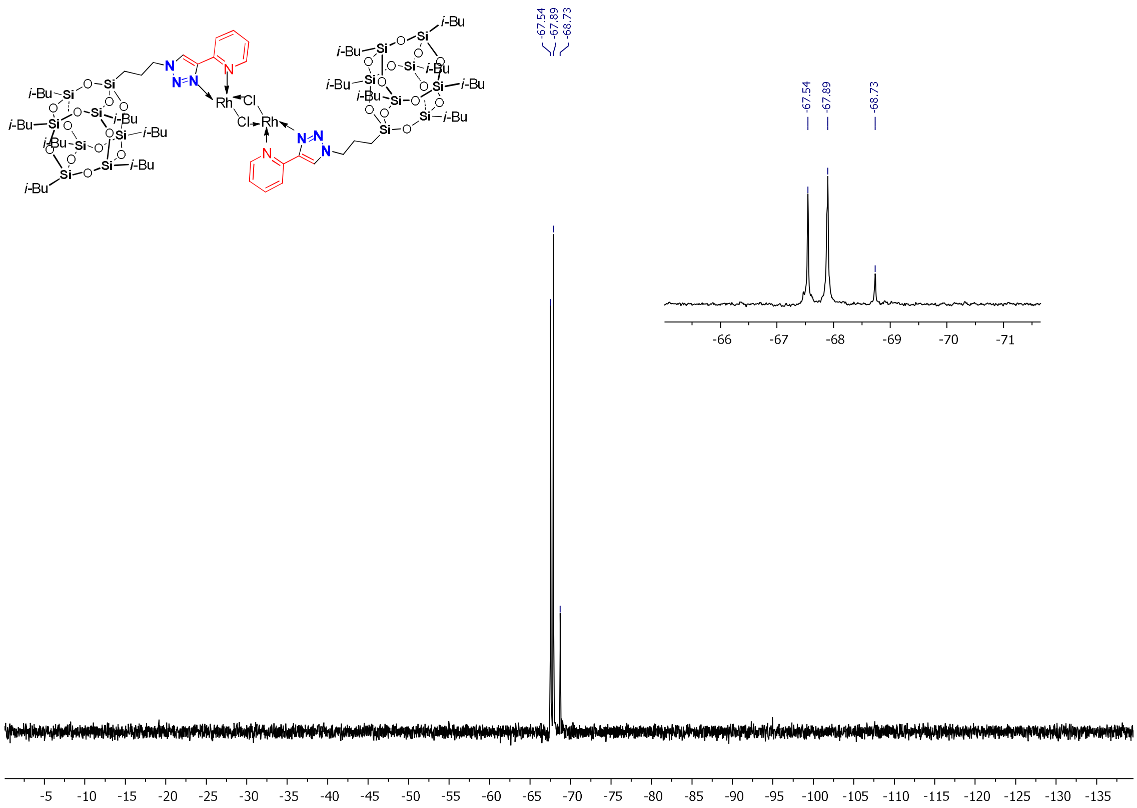

Figure S42 ^{29}Si NMR spectra of (iBuT$_8$-A1)$_2$-Rh(N^N)

iBuT*$_8$*-A1-Pt(N^N)

Yellow solid, 87%

^{1}H NMR (300 MHz, CD_2Cl_2, 25 °C) δ = 9.19 (d, J_{H-H} = 5.9 Hz,), 8.52 (s,), 8.12-7.98 (m,), 7.37-7.33 (m,), 4.56 (t, J_{H-H} = 7.2 Hz,), 2.09 (dt, J_{H-H} = 15.3, 7.4 Hz,), 1.86 (dd, J_{H-H} = 13.2, 9.2 Hz,), 0.95 (d, J_{H-H} = 6.6 Hz,), 0.63-0.59 (m,); ^{13}C NMR (101 MHz, CD_2Cl_2, 25 °C) δ = 150.02, 149.28, 149.03, 140.22, 137.27, 125.76, 125.70, 123.22, 122.85, 122.44, 120.32, 56.20, 26.05, 26.03, 24.49, 24.42, 24.26, 23.01, 22.95, 22.89, 9.76; ^{29}Si NMR (79.5 MHz, CD_2Cl_2, 25 °C) δ = -67.40, -67.79, -69.04; IR (cm^{-1}): 3092.64, 2952.61, 2905.71, 2869.24, 1625.06, 1464.15, 1365.69, 1331.72, 1228.25, 1167.93, 1095.88, 1037.28, 836.73, 741.60; EA: Anal. calcd for $C_{38}H_{74}Cl_2N_4O_{12}PtSi_8$ (%): C, 35.95, H, 5.87; found: C, 36.09; H, 5.93.

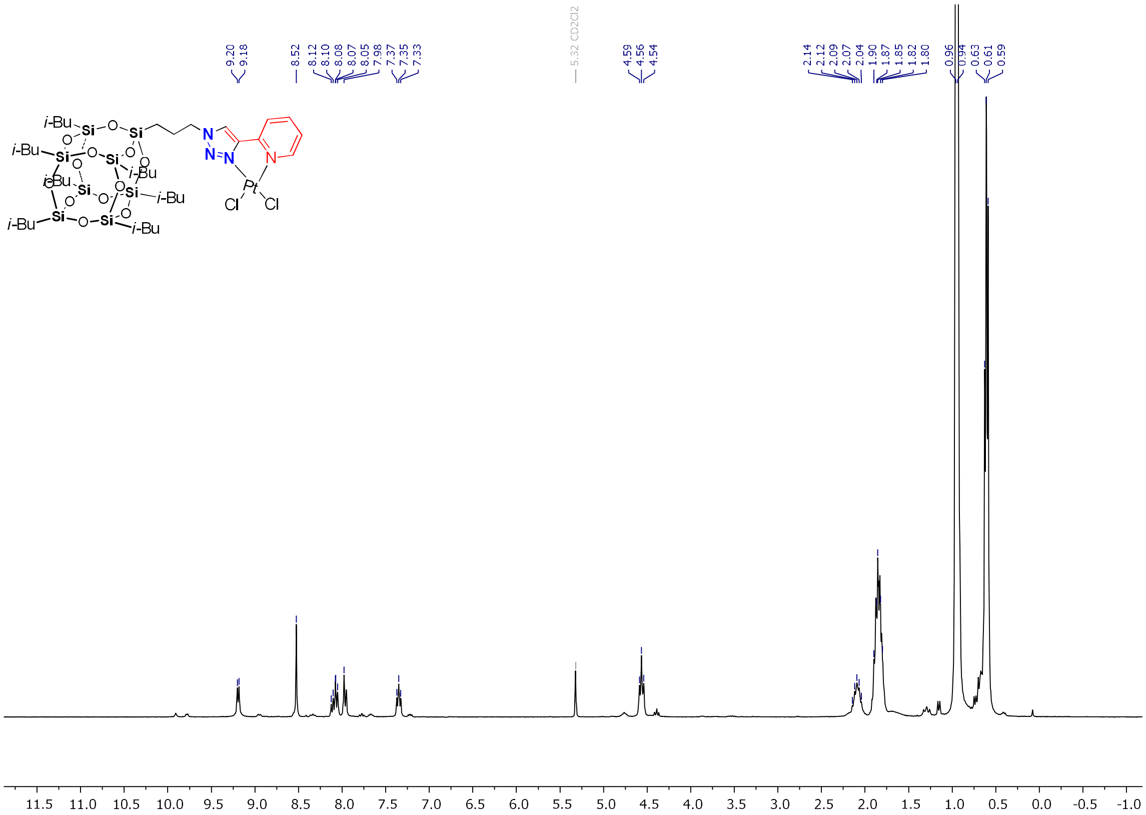

Figure S43 ^{1}H NMR spectra of iBuT$_8$-A1-Pt(N^N)

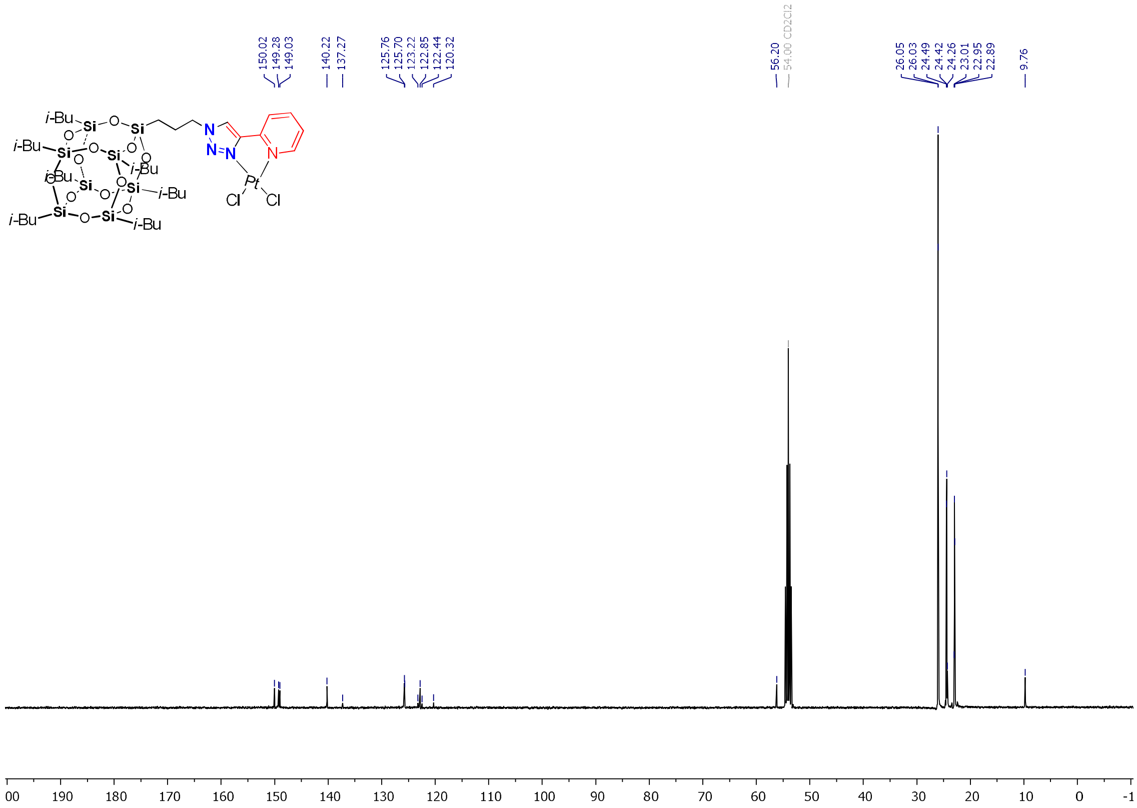

Figure S44 ^{13}C NMR spectra of iBuT$_8$-A1-Pt(N^N)

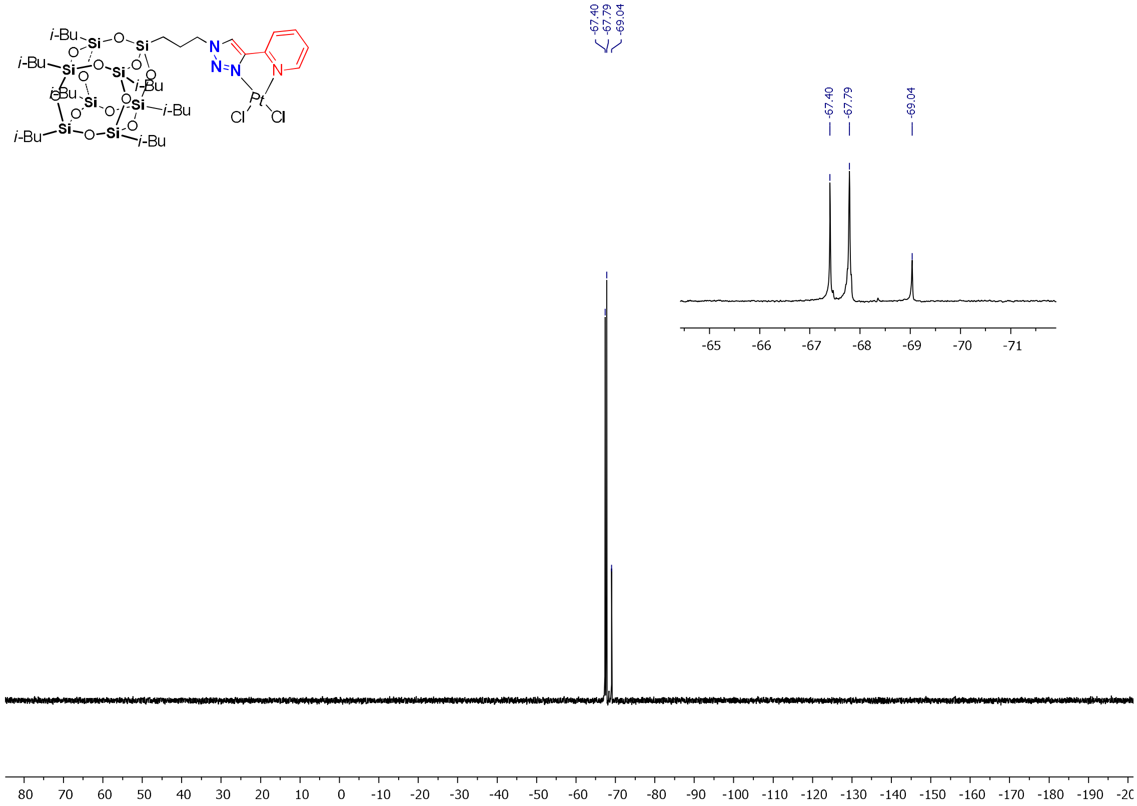

Figure S45 ^{29}Si NMR spectra of iBuT$_8$-A1-Pt(N^N)

iBuT$_8$-A7-Pd(N^S)

Yellow solid, 60%

^{1}H NMR (300 MHz, $CDCl_3$, 25 °C) δ = 8.33 (s, 1H, PyH), 7.65 (s, 1H, PyH), 7.53 (s, 1H, NCH), 7.51 (s, 1H, PyH), 7.22 (s, 1H), 4.41 (t, 2H, $J_{H\text{-}H}$ = 7.8 Hz, N-CH_2), 2.07 (q, 2H, CH_2-CH_2-CH_2-), 1.88-1.81 (m, 7H, $CH(CH_3)_2$, 0.98-0.94 (m, 42H, $(CH_3)_2CHCH_2Si$), 0.64-0.62 (m, 14H, $SiCH_2$); ^{13}C NMR (101 MHz, $CDCl_3$, 25 °C) δ = 143.97, 129.14, 128.56, 127.93, 127.72, 121.98, 54.32, 25.85, 25.82, 24.06, 24.04, 24.00, 23.97, 23.70, 23.62, 22.60, 2258, 9.42; ^{29}Si NMR (79.5 MHz, $CDCl_3$, 25 °C) δ = -67.45, -67.83, -67.86, -69.02; IR (cm^{-1}): 3074.10, 2952.38, 2925.98, 2869.07, 1464.33, 1331.82, 1228.06, 1091.63, 1037.85, 741.12, 479.99; EA: Anal. calcd for $C_{37}H_{73}Cl_2N_3O_{12}PdSSi_8$ (%): C, 37.47, H, 6.20; found: C, 37.49; H, 6.33.

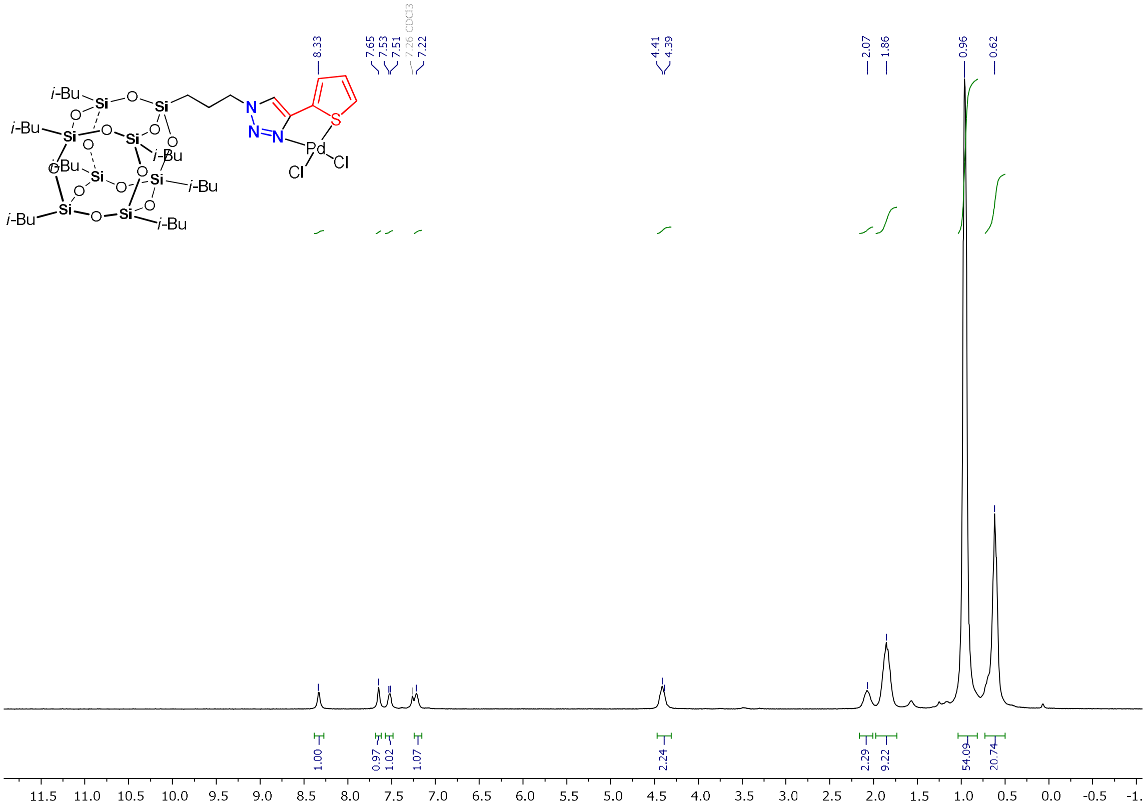

Figure S46 ^{1}H NMR spectra of iBuT$_8$-A7-Pd(N^S)

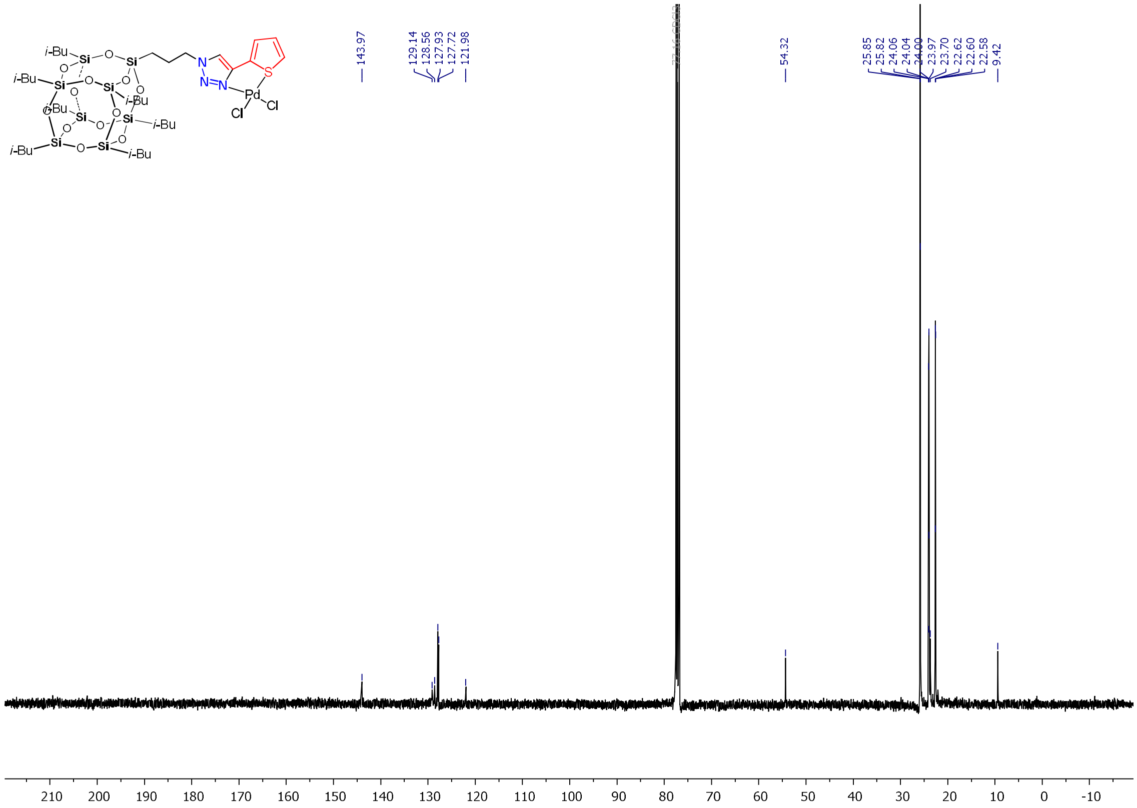

Figure S47 ^{13}C NMR spectra of iBuT$_8$-A7-Pd(N^S)

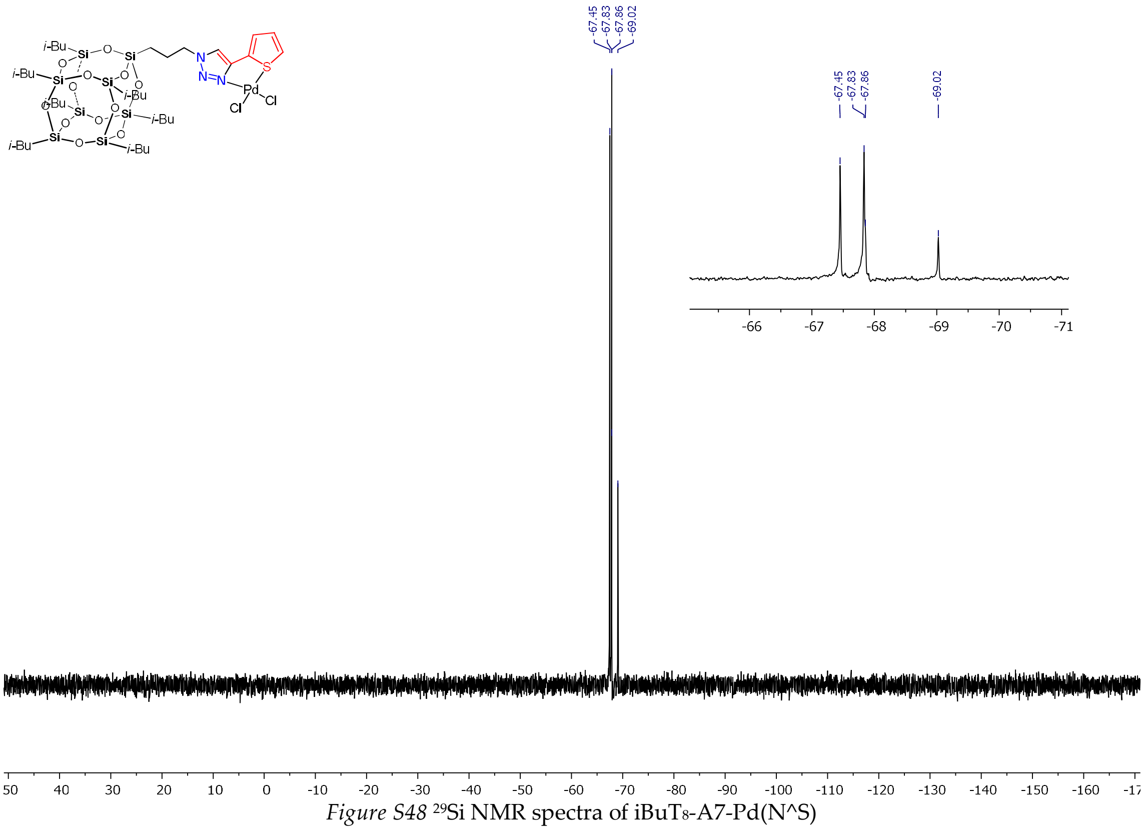

Figure S48 ^{29}Si NMR spectra of iBuT$_8$-A7-Pd(N^S)

DDSQ-A1-[Pd(N^N)]$_2$

Yellow solid, 93%

^{1}H NMR (300 MHz, DMF-d$_7$, 25 °C) δ = 9.14 (d, 2H, J=1.4Hz, NCH), 9.09 (dd, 2H, J = 5.8, 1.3 Hz, PyH), 8.31 (t, 2H, J = 7.7 Hz, PyH), 8.15 – 8.09 (m, 2H, PyH), 7.72 (m, 2H, PyH), 7.64 – 7.60 (m, 5H, Ph), 7.49–7.23 (m, 35H, Ph), 4.61 (t, 4H, J=7.1Hz, N-CH$_2$), 2.12 (d, 4H, J=8.8Hz, CH$_2$CH$_2$CH$_2$), 0.98–0.91 (m, 4H, CH$_2$Si), 0.43 (s, 6H, CH$_3$Si); ^{13}C NMR (101 MHz, DMF-d$_7$, 25 °C) δ = 150.82, 149.88, 148.84, 142.38, 134.85, 134.82, 134.79, 132.31, 132.21, 132.18, 131.45, 129.39, 129.33, 129.29, 129.21, 126.56, 126.50, 123.13, 55.80, 24.76, 14.11, -0.49; ^{29}Si NMR (79.5 MHz DMF-d$_7$, 25 °C) δ = -17.16, -77.78, -79.04; ESI-MS calcd for $C_{38}H_{74}N_4O_{12}Si_8PdCl_2K$ [M + H]+: 1876.9450, found 1877.0601; IR (cm^{-1}): 3072.03, 3048.83, 2921.62, 1429.57, 1264.09, 1078.01, 727.16, 696.17, 482.53; Anal. calcd for $C_{70}H_{68}Cl_4N_8O_{14}Pd_2Si_{10}$ (%):C, 44.70, H, 3.64; found: C, 44.79; H, 3.71.

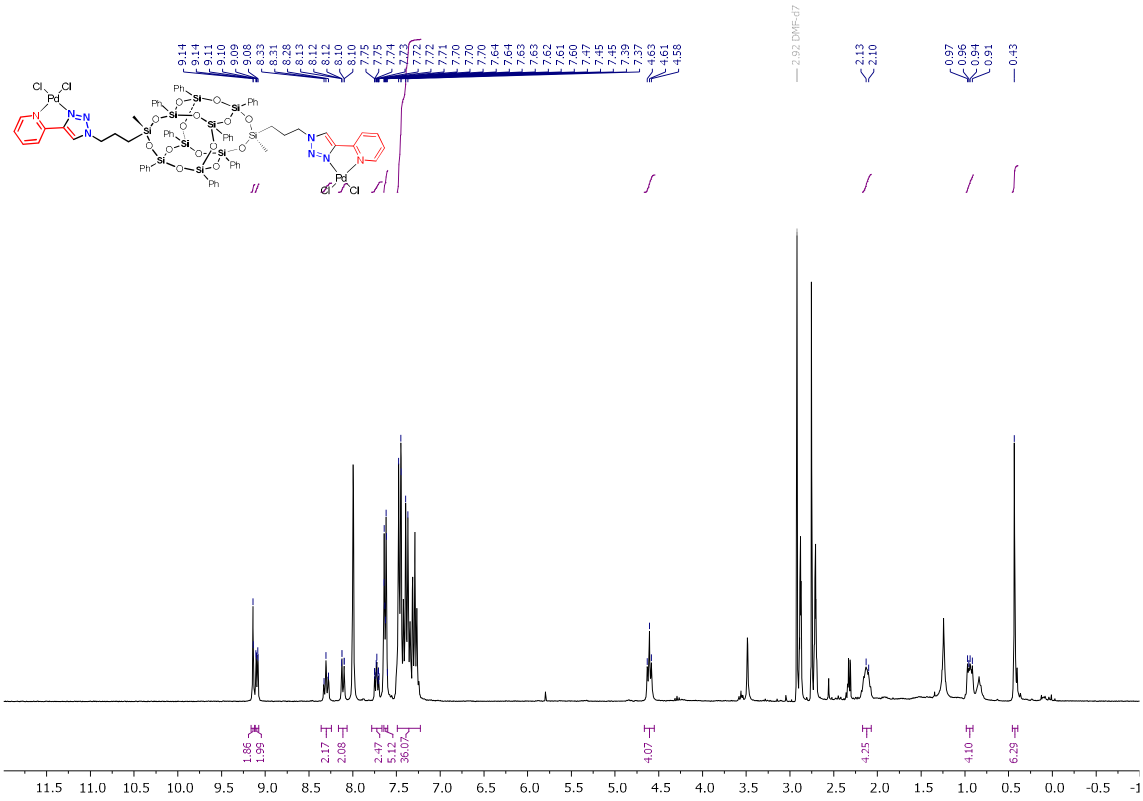

Figure S49 ^{1}H NMR spectra of DDSQ-A1-[Pd(N^N)]$_2$

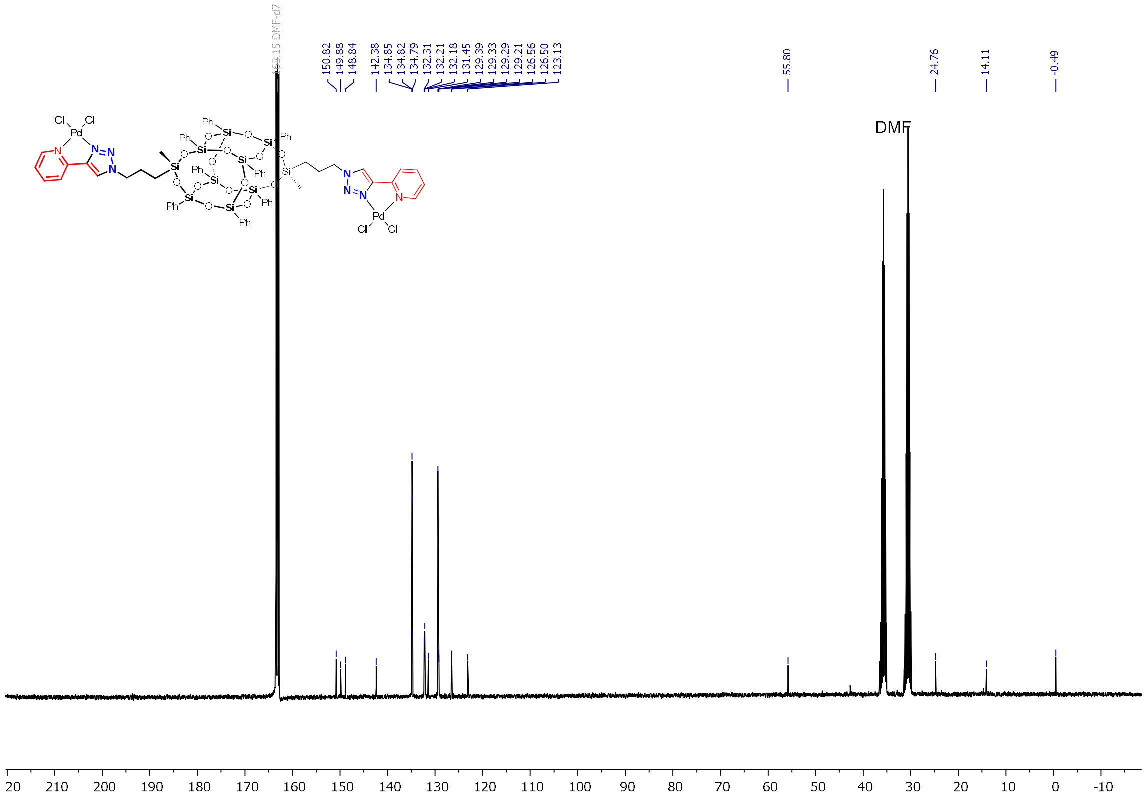

Figure S50 ^{13}C NMR spectra of DDSQ-A1-[Pd(N^N)]$_2$

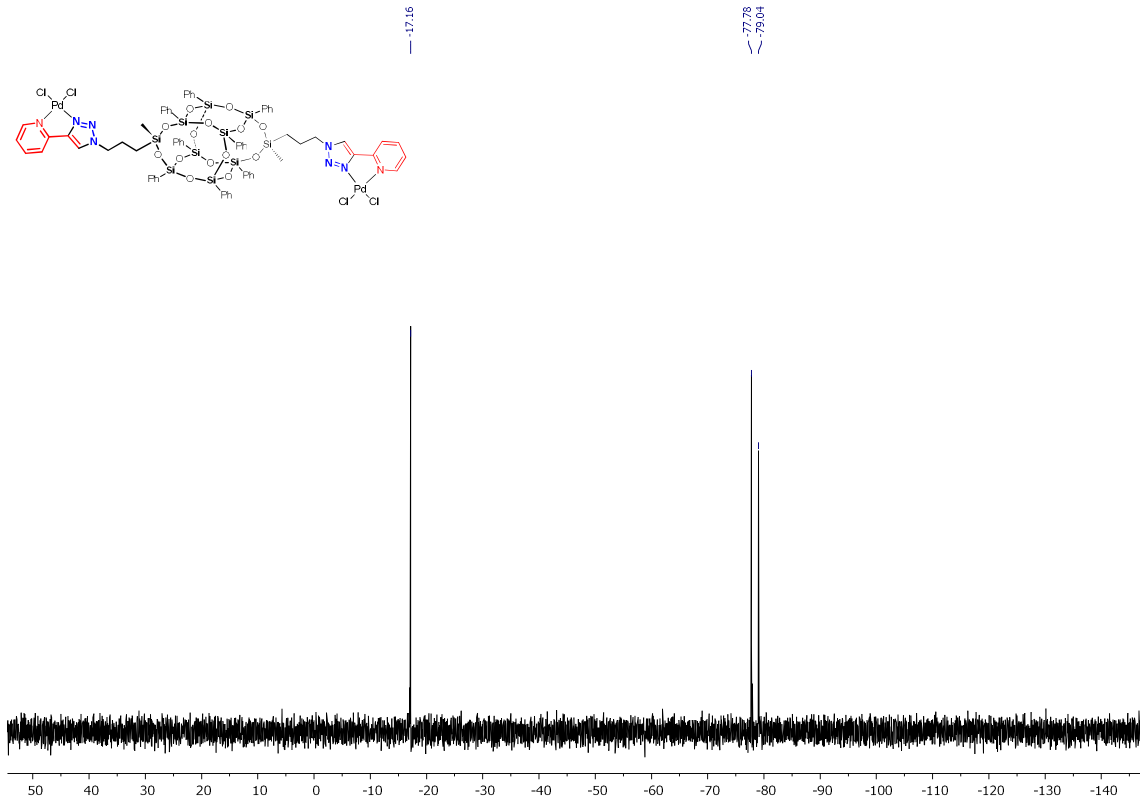

Figure S51 ^{29}Si NMR spectra of DDSQ-A1-[Pd(N^N)]$_2$

The respective comparison of the ^{1}H NMR stacked spectra of ligand iBuT$_8$-A1 and respective complex iBuT8-A1-Pt(N^N) are presented below:

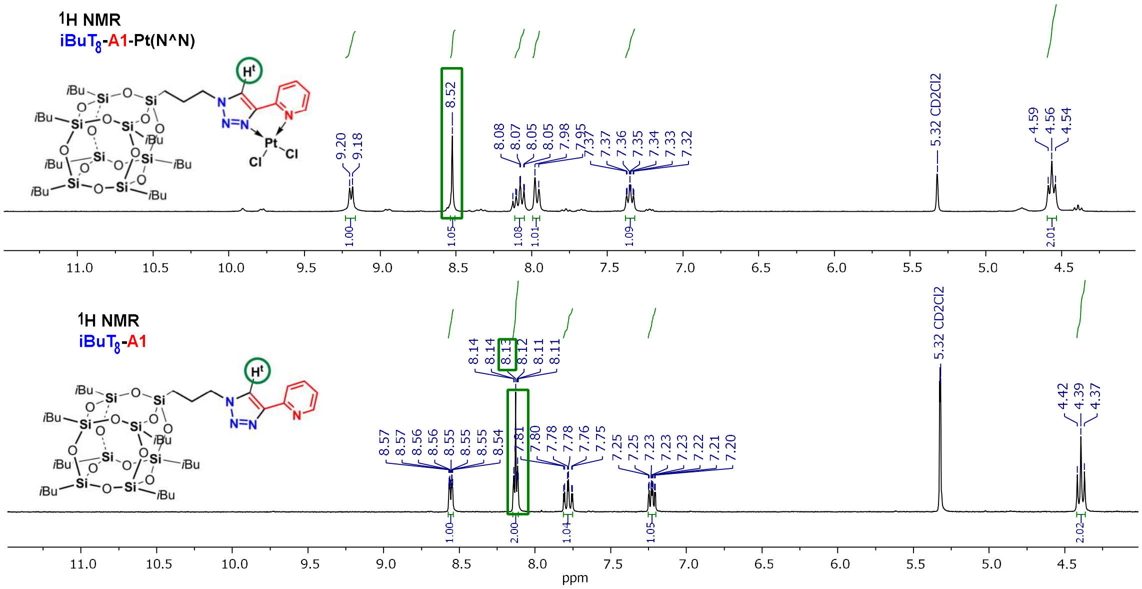

Figure S52 Stacked ^{1}H NMR spectra of ligand **iBuT$_8$-A1** and respective complex **iBuT$_8$-A1-Pt(N^N)**

The respective comparison of the ^{1}H NMR stacked spectra of ligand iBuT$_8$-A7 and respective complex iBuT$_8$-A7-Pd(N^S) are presented below:

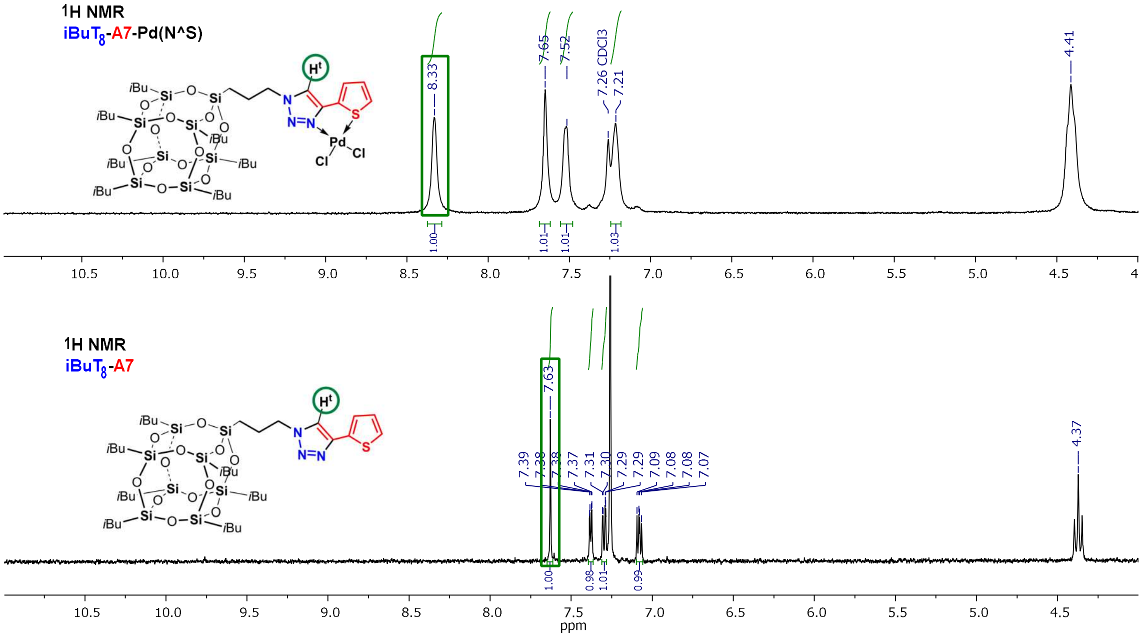

Figure S53 Stacked ^{1}H NMR spectra of ligand **iBuT$_8$-A7** and respective complex **iBuT$_8$-A7-Pt(N^S)**

References:

[1] Ervithayasuporn, V.; Kwanplod, K.; Boonmak, J.; Youngme, S,; Sangtrirutnugul, P.; Homogeneous and heterogeneous catalysts of organopalladium functionalized-polyhedral oligomeric silsesquioxanes for Suzuki–Miyaura reaction. *J. of Catal.* **2015**, *332*, 62-69.
